# Direct stimulation of de novo nucleotide synthesis by *O*-GlcNAcylation

**DOI:** 10.1038/s41589-023-01354-x

**Published:** 2023-06-12

**Authors:** Lulu Chen, Qi Zhou, Pingfeng Zhang, Wei Tan, Yingge Li, Ziwen Xu, Junfeng Ma, Gary M. Kupfer, Yanxin Pei, Qibin Song, Huadong Pei

**Affiliations:** 1 Cancer Center, Renmin Hospital of Wuhan University, Wuhan, China; 2 Department of Oncology, Georgetown Lombardi Comprehensive Cancer Center, Georgetown University Medical Center, Washington, DC USA; 3 Department of Lymphoma and Myeloma, The University of Texas MD Anderson Cancer Center, Houston, TX USA; 4 Center for Cancer and Immunology, Brain Tumor Institute, Children’s National Health System, Washington, DC USA

**Keywords:** Post-translational modifications, Glycobiology, Cancer therapy, DNA metabolism, Enzyme mechanisms

## Abstract

*O*-linked β-*N*-acetyl glucosamine (*O*-GlcNAc) is at the crossroads of cellular metabolism, including glucose and glutamine; its dysregulation leads to molecular and pathological alterations that cause diseases. Here we report that *O*-GlcNAc directly regulates de novo nucleotide synthesis and nicotinamide adenine dinucleotide (NAD) production upon abnormal metabolic states. Phosphoribosyl pyrophosphate synthetase 1 (PRPS1), the key enzyme of the de novo nucleotide synthesis pathway, is *O*-GlcNAcylated by *O*-GlcNAc transferase (OGT), which triggers PRPS1 hexamer formation and relieves nucleotide product-mediated feedback inhibition, thereby boosting PRPS1 activity. PRPS1 *O*-GlcNAcylation blocked AMPK binding and inhibited AMPK-mediated PRPS1 phosphorylation. OGT still regulates PRPS1 activity in AMPK-deficient cells. Elevated PRPS1 *O*-GlcNAcylation promotes tumorigenesis and confers resistance to chemoradiotherapy in lung cancer. Furthermore, Arts-syndrome-associated PRPS1 R196W mutant exhibits decreased PRPS1 *O*-GlcNAcylation and activity. Together, our findings establish a direct connection among *O*-GlcNAc signals, de novo nucleotide synthesis and human diseases, including cancer and Arts syndrome.

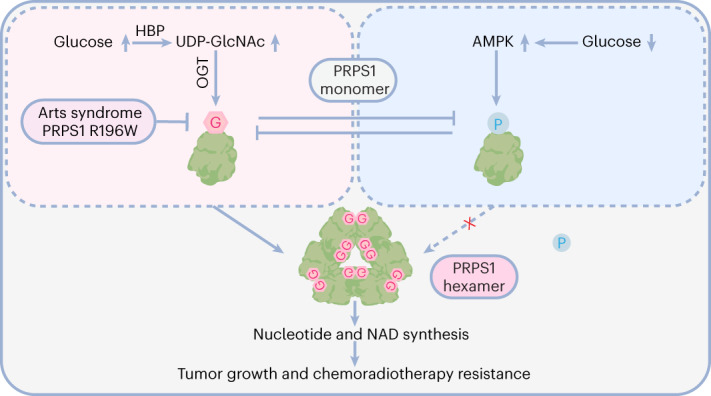

## Abstract (web summary)

Phosphoribosyl pyrophosphate synthetase (PRPS1), an enzyme that regulates de novo nucleotide synthesis, was found to be *O*-GlcNAcylated, which increases PRPS1 activity, and is associated with increased tumorigenesis and resistance to chemoradiotherapy.

## Main

**Fig. 1:**
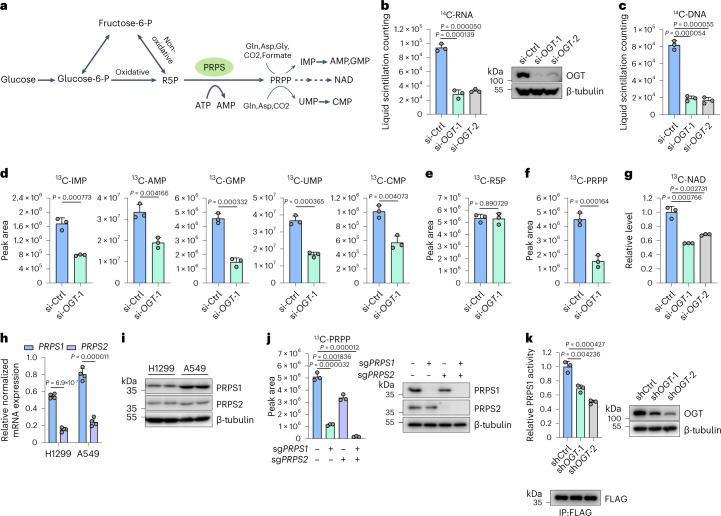
OGT regulates nucleotide synthesis and NAD production. **a**, The scheme of PRPS-catalyzed reaction showing the metabolic pathway of de novo synthesis of nucleotides and NAD production from glucose-derived pentose phosphate pathway. **b**,**c**, H1299 cells were transfected with the indicated siRNAs and cultured with 1 μCi of ^14^C-glucose. The de novo synthesized ^14^C-RNA (**b**) or ^14^C-DNA (**c**) was measured by liquid scintillation counting. Immunoblot with the indicated antibodies was performed to confirm the knockdown efficiency of siRNAs targeting *OGT* (*n* = 3). **d**–**g**, The indicated siRNAs were introduced into H1299 cells. Cells were incubated in medium containing ^13^C_6_-glucose. High-resolution LC–MS/MS was used to analyze ^13^C_6_-labeled nucleotide monophosphates (**d**), R5P (**e**), PRPP (**f**) and NAD (**g**) (*n* = 3). **h**,**i**, The mRNA levels (**h**) (*n* = 4) and protein levels (**i**) (*n* = 4) of PRPS1 and PRPS2 in H1299 and A549 cell lines were measured by qPCR and immunoblot analyses, respectively. **j**, The WT, PRPS1 knockout, PRPS2 knockout and double-knockout H1299 cells were incubated in medium containing ^13^C_6_-glucose. LC–MS/MS was conducted to measure ^13^C-PRPP levels (*n* = 3). The immunoblotting was conducted with indicated antibodies. **k**, FLAG-PRPS1 was purified from indicated shRNA-transduced H1299 cells and subjected to PRPS1 enzymatic activity assay. The intensity of FLAG-PRPS1 immunoblot was calculated to quantify PRPS1 activity (*n* = 3). Each error bar represents mean ± s.e.m. A two-tailed Student’s *t*-test was employed for statistical evaluation. Source data

As a distinguishing hallmark of cancer, metabolic reprogramming enhances the production of glycolytic intermediates to synthesize cellular building substances, such as nucleotides, lipids and amino acids^1,2^. A supply of nucleotides is essential for rapid cancer cell proliferation^3,4^. Phosphoribosyl pyrophosphate synthetase 1 (PRPS1) acts as the first rate-limiting enzyme to produce nucleic acid precursors by converting ribose 5-phosphate (R5P) into phosphoribosyl pyrophosphate (PRPP)^5,6^ (Fig. 1a). PRPP is a crucial intermediate in multiple cellular metabolic pathways, including the synthesis of purine and pyrimidine nucleotides, histidine, tryptophan, nicotinamide adenine dinucleotide (NAD) and nicotinamide adenine dinucleotide phosphate (NADP)^7^ (Fig. 1a).

In *Homo sapiens*, three distinct isoforms of PRPS, which share a high degree of similarity in their sequences, have been identified. PRPS1 and PRPS2 are expressed across a broad array of tissues, whereas PRPS3 expression appears exclusive to testis^8,9^. PRPS1 exists in dynamic monomer, dimer and hexamer forms, and the hexamers serve as active units^10^. PRPS1 uses ATP as an energy source to facilitate the transformation of R5P into PRPP, simultaneously generating AMP as a byproduct^10^. On the other hand, the PRPS reaction is subject to feedback inhibition by products of nucleotide biosynthesis, such as ADP, AMP and GDP^8,9^. Nutritional flux affects nucleotide synthesis directly or indirectly. For example, the energy sensor AMPK directly regulates de novo nucleotide synthesis under metabolic stress conditions. Mechanistically, glucose deprivation leads to PRPS1 serine 180 (S180) and PRPS2 serine 183 (S183) phosphorylation mediated by AMPK, inhibiting PRPS1 and PRPS2 activity as well as subsequent nucleic acid synthesis^11^.

PRPS1 dysregulation is associated with multiple diseases. Aberrant activation of PRPS1 has been observed in colorectal cancer^12,13^, hepatocellular carcinoma^14^, breast cancer^15^ and T cell acute lymphoblastic leukemia (ALL)^16^. PRPS1 uninhibitable activation also results in thiopurine resistance in relapsed childhood ALL^17^. Decreased PRPS1 activity is also linked to disorders such as Arts syndrome and retinal dystrophy^18^, whereas its superactivity is accountable for neurosensory deficits, hyperuricemia and gouty arthritis^19,20^.

*O*-linked β-*N*-acetylglucosamine (*O*-GlcNAc) represents a glycosylation event where a sugar molecule is attached to the hydroxyl groups of serine or threonine residues on specific target proteins. In this procedure, referred to as *O*-GlcNAcylation, UDP-GlcNAc serves as the sugar donor, originating from glucose through the hexosamine biosynthetic pathway (HBP)^21–23^. Apart from being a sensor of glucose, UDP-GlcNAc is also modulated by metabolisms of other cellular nutrients, including fatty acids (acetyl-CoA) and amino acids (glutamine), thereby signifying the energy state of cells. *O*-GlcNAcylation is catalyzed by *O*-GlcNAc transferase (OGT), whereas its reversal, de-*O*-GlcNAcylation, is facilitated by β-*N*-acetylglucosaminidase (OGA)^21,24–26^. *O*-GlcNAcylation plays important roles in physiologic and pathophysiologic events, such as transcription, proliferation, differentiation, metabolic homeostasis, inflammation, immunity, development and tumorigenesis^27–34^. However, the details of the functions and underlying mechanisms of *O*-GlcNAc in cancers remain mostly unclear.

In the current research, we revealed that *O*-GlcNAc directly regulates de novo nucleotide synthesis. Mechanistically, PRPS1 serine 83 (S83) and threonine 166 (T166) *O*-GlcNAcylation mediated by OGT leads to PRPS1 hexamer formation and reduction of feedback inhibition of PRPS1 activity, thereby promoting PRPS1 activation, nucleotide synthesis and NAD production. Moreover, PRPS1 *O*-GlcNAcylation promoted tumorigenesis and affected cancer cell response to chemoradiotherapy.

## Results

### OGT regulates nucleotide synthesis and NAD production

**Extended Data Fig. 1:**
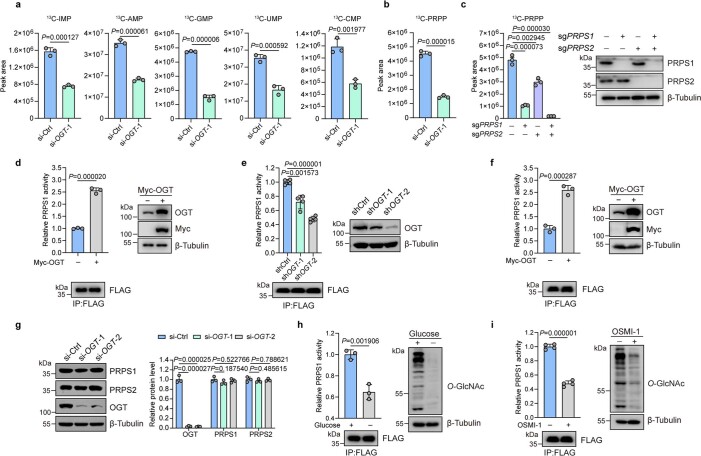
OGT regulates de novo nucleotide synthesis, not by affecting PRPS1 and PRPS2 protein levels. **a**,**b**, The indicated siRNAs were introduced into A549 cells. Cells were incubated in medium containing ^13^C_6_-glucose. LC/MS-MS analysis was performed to measure the levels of ^13^C_6_ labeled nucleotide monophosphates (**a**) and PRPP (**b**). (n = 3). **c**, The wild-type, PRPS1 knockout, PRPS2 knockout, and double knockout A549 cells were incubated in the medium containing ^13^C_6_-glucose. LC/MS-MS analysis was performed to measure the incorporation of ^13^C_6_-glucose into intracellular PRPP . The proteins purified from the cells were subject to immunoblot analyses with the indicated antibodies. (n = 3). **d**, The indicated plasmids were introduced into H1299 cells. FLAG-PRPS1 was purified and subjected to PRPS1 enzymatic activity assay. Immunoblotting was conducted with indicated antibodies. The intensity of FLAG-PRPS1 immunoblots was used to quantify PRPS1 activity. (n = 3). **e**,**f**, The indicated shRNAs were introduced into HEK293T cells (n = 3) (**e**). Myc-vector or Myc-*OGT* plasmids were introduced into HEK293T cells (n = 3) (**f**). The cells from all groups were transfected with FLAG-PRPS1 plasmids. FLAG-PRPS1 was purified and subjected to PRPS1 enzymatic activity assay. The intensity of FLAG-PRPS1 immunoblots was used to quantify PRPS1 activity. **g**, The indicated siRNAs were introduced into H1299 cells. Immunoblotting was conducted with indicated antibodies. The relative protein expression were quantified. (n = 3). **h**,**i**, The FLAG-PRPS1 plasmids were introduced into HEK293T cells. The cells were cultured with or without glucose for 24 h (n = 3) (**h**) or treated with or without 50 µM OSMI-1 for 24 h (n = 4) (**i**). FLAG-PRPS1 was purified and subjected to PRPS1 enzymatic activity assay. Immunoblotting was conducted with indicated antibodies. Intensities of FLAG-PRPS1 immunoblots were used to quantify the PRPS1 activity. Each error bar represents mean±SEM. Statistical analysis was done using a two-tailed Student’s t-test. Source data

Cancer cells exhibit abnormal glucose metabolism and aberrant *O*-GlcNAcylation level, implying that the flux through the HBP may be altered^21,23,26,35^. On the other hand, cancer cells have reprogrammed glucose metabolism to accelerate de novo nucleotide biosynthesis^4,36^. To explore the direct regulatory role of *O*-GlcNAcylation in de novo nucleotide synthesis, we used siRNAs to silence OGT in H1299 cells. Subsequently, we monitored de novo nucleotide synthesis in cells incubated with either ^14^C-glucose or ^13^C_6_-glucose. As illustrated in Fig. 1b,c, OGT knockdown substantially decreased ^14^C-labeled RNA and DNA levels derived from glucose. Both ^13^C-labeled purine intermediate (IMP, AMP and GMP) and pyrimidine intermediate (UMP and CMP) levels were reduced in OGT-deficient H1299 cells and A549 cells (Fig. 1d and Extended Data Fig. 1a). However, silencing OGT did not affect PPP-derived R5P production (Fig. 1e). PRPP levels decreased in OGT-deficient cells (Fig. 1f and Extended Data Fig. 1b). These findings illustrate that OGT regulates de novo nucleotide synthesis by controlling the conversion of R5P to PRPP.

In addition to its role in nucleotide synthesis, PRPP also serves as an intermediate in NAD production^7^. OGT knockdown decreased ^13^C-labeled NAD production (Fig. 1g), indicating that OGT also plays a role in regulating NAD biosynthesis.

The conversion of R5P to PRPP, catalyzed by PRPS, is a process that limits the rate of nucleotide synthesis. Two isoforms (PRPS1 and PRPS2) in lung cancer cells have a similar enzymatic function. PRPS1 mRNA level and protein level were higher than those of PRPS2 in H1299 cells and A549 cells (Fig.1h,i). We further knocked down PRPS1, PRPS2 or PRPS1 plus PRPS2 in these two cells, respectively, and checked cellular PRPP production. As shown in Fig. 1j and Extended Data Fig. 1c, both PRPS1 and PRPS2 promoted PRPP production, and PRPS1 contributed more than PRPS2. In line with the findings above, PRPS1 activity decreased or increased in OGT knockdown or OGT overexpressed cells, respectively (Fig. 1k and Extended Data Fig. 1d–f). However, protein levels did not change considerably (Extended Data Fig. 1g). Consistently, glucose starvation or OGT inhibitor treatment also inhibited PRPS1 activity (Extended Data Fig. 1h,i). These findings suggest that OGT promotes PRPS1 activity, leading to increased nucleotide synthesis.

### OGT interacts with and *O*-GlcNAcylates PRPS1

**Fig. 2:**
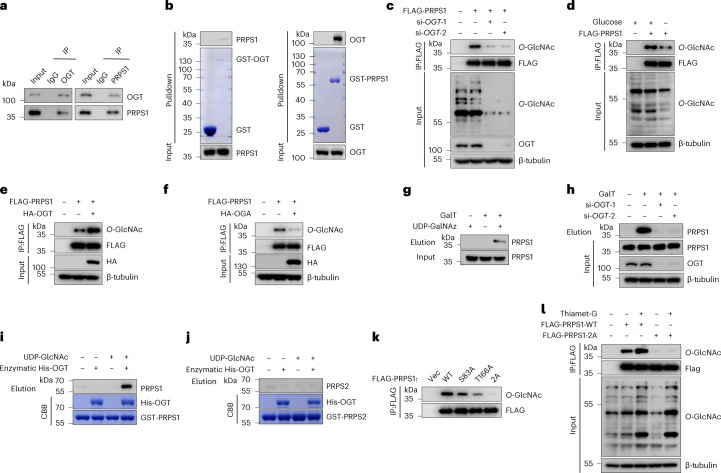
OGT interacts with and *O*-GlcNAcylates PRPS1. **a**, Reciprocal endogenous immunoprecipitation (IP) assay between OGT and PRPS1 in HEK293T cells (*n* = 3). **b**, GST, GST-OGT and GST-PRPS1 proteins were expressed and purified from *E. coli* and subjected to in vitro pulldown of endogenous PRPS1 or OGT from HEK293T cells (*n* = 3). **c**–**f**, The indicated siRNAs were introduced into H1299 cells (**c**); H1299 cells were then cultured with or without glucose for 24 h (**d**), transfected with or without HA-OGT plasmids (**e**) and transfected with or without HA-OGA plasmids (**f**). Cells from all groups were transfected with vector or FLAG-PRPS1 plasmids. IP and immunoblot analyses were conducted with anti-FLAG agarose beads and the indicated antibodies (*n* = 3). **g**,**h**, PRPS1 *O*-GlcNAcylation analysis using a chemoenzymatic labeling method in HEK293T cells. **g**, Immunoblot of *O*-GlcNAcylated PRPS1 in elution and total PRPS1 in input. **h**, The indicated siRNAs were introduced into HEK293T cells. Immunoblotting was conducted with indicated antibodies (*n* = 3). **i**,**j**, The recombinant proteins of GST-PRPS1 and GST-PRPS2 and enzymatic His-OGT domain (aa 323–1,041) were incubated in the in vitro *O*-GlcNAcylation assay reaction buffer. PRPS1 (**i**) and PRPS2 (**j**) *O*-GlcNAcylation was analyzed by the chemoenzymatic labeling method. Immunoblot analyses and Coomassie blue staining were performed (*n* = 3). **k**, The indicated plasmids were introduced into HEK293T cells. FLAG-PRPS1 was immunoprecipitated and subjected to immunoblot analyses with indicated antibodies (*n* = 4). **l**, HEK293T cells were genetically modified as indicated and treated with or without thiamet-G (2 μM) for 3 h. IP and immunoblotting were conducted with anti-FLAG agarose beads and indicated antibodies (*n* = 3). Source data

**Extended Data Fig. 2:**
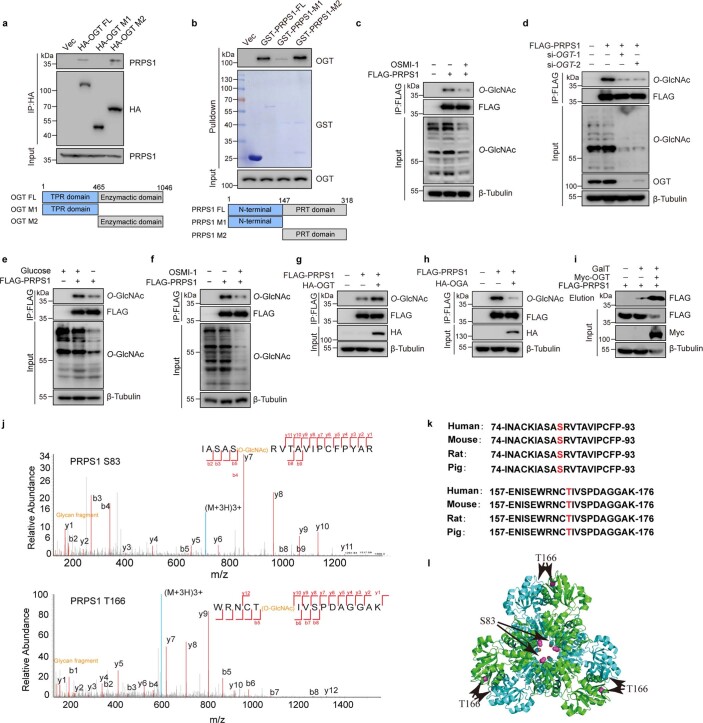
OGT interacts with PRPS1 and OGT *O*-GlcNAcylates PRPS1 at S83 and T166. **a**,**b**, The plasmids encoding full-length (FL) or truncated forms of OGT (M1 and M2) were introduced into HEK293T cells (**a**). GST, GST-PRPS1 full-length (FL), and truncated mutants (M1 and M2) proteins were purified from *E. coli* BL21 cells (**b**). Immunoprecipitation/ pull-down and immunoblot were performed. Schematics illustrating OGT and PRPS1 truncated mutants are presented. OGT TPR domain represents OGT tetratricopeptide repeat (TPR) domain; PRPS1 PRT domain represents PRPS1 phosphoribosyl transferase (PRT)-type I domain. (n = 3). **c**, The indicated plasmids were introduced into H1299 cells. Cells were treated with or with no OSMI-1 (50 µM) for 24 h. Immunoprecipitation and immunoblot analyses were performed with anti-FLAG beads and indicated antibodies. (n = 3). **d**-**h**, The indicated siRNAs were introduced into A549 cells (**d**); A549 cells were cultured with or without glucose for 24h (**e**); A549 cells were treated with or without OSMI-1 (50 µM) for 24h (**f**); A549 cells were transfected with or without HA-OGT plasmids (**g**); transfected with or without HA-OGA plasmids (**h**). The cells from all groups were transfected with FLAG-vector or FLAG-PRPS1 plasmids. Immunoprecipitation and immunoblot analyses were conducted with anti-FLAG beads and indicated antibodies. (n = 3). **i**, HEK293T cells were transfected with or without Myc-OGT plasmids. PRPS1 *O*-GlcNAcylation was analyzed using the chemoenzymatic labeling method. Immunoblotting was conducted with the indicated antibodies. (n = 3). **j**, Mass spectrometry analyses of PRPS1 *O*-GlcNAcylation sites. **k**, Alignment of protein sequences containing PRPS1 S83 (top) and T166 (bottom) sites from different species. **l**, Modeling of human PRPS1 hexamer structure (Protein Data Bank code: 2H06). The S83 and T166 residues are indicated. Source data

Based on these results, we hypothesized that OGT *O*-GlcNAcylates PRPS1 and enhances its activity. Consistent with this hypothesis, OGT interacted with PRPS1 in cells and in vitro (Fig. 2a,b). The C-terminal catalytic domain of OGT interacted with PRPS1, whereas the C-terminal of PRPS1 (amino acids (aa) 148–318) bound to OGT (Extended Data Fig. 2a,b). Furthermore, knockdown of OGT, glucose starvation or OGT inhibitor treatment considerably decreased PRPS1 *O*-GlcNAcylation (Fig. 2c,d and Extended Data Fig. 2c). Overexpressed OGT led to increased *O*-GlcNAcylation of PRPS1, whereas decreased *O*-GlcNAcylation of PRPS1 was observed upon overexpression of OGA. (Fig. 2e,f). Similar results were observed in A549 cells (Extended Data Fig. 2d–h).

To verify the *O*-GlcNAcylation of PRPS1 in cells, we conducted a chemoenzymatic labeling experiment as previously reported^37^. Immunoblotting analysis demonstrated detectable signals in the experimental group but not in the control groups, providing evidence that PRPS1 was indeed *O*-GlcNAcylated (Fig. 2g). As anticipated, knockdown or overexpression of OGT decreased or increased immunoblotting signals considerably (Fig. 2h and Extended Data Fig. 2i), indicating that OGT *O*-GlcNAcylated PRPS1 in cells. Furthermore, OGT directly *O*-GlcNAcylated PRPS1 but not PRPS2 in vitro (Fig. 2i,j), indicating the specificity of this modification.

To identify *O*-GlcNAcylation sites on PRPS1, we purified PRPS1 from HEK293T cells and identified the sites of *O*-GlcNAcylation on the protein through liquid chromatography with tandem mass spectrometry (LC–MS/MS) analysis. As illustrated in Extended Data Fig. 2j, S83 and T166 were potential *O*-GlcNAcylation sites on PRPS1. These two sites are conserved in mice, rats and other species (Extended Data Fig. 2k). S83 and T166 are located at the inner side and the outer side of PRPS1 hexamer, respectively (Extended Data Fig. 2l). Furthermore, we found that *O*-GlcNAcylation-deficient mutations of both S83 and T166, but not only one residue, markedly reduced *O*-GlcNAcylation signals, suggesting that S83 and T166 are the major *O*-GlcNAcylation sites (Fig. 2k,l).

### *O*-GlcNAcylation boosts PRPS1 activity

**Fig. 3:**
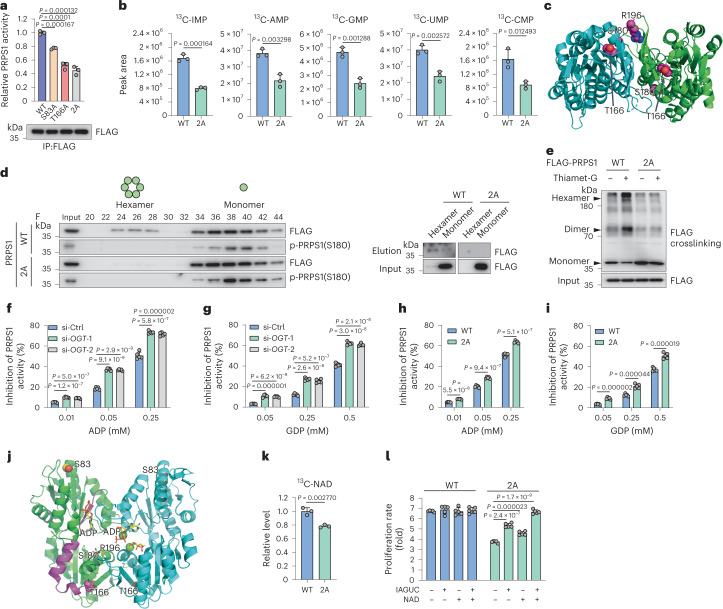
*O*-GlcNAcylation boosts PRPS1 activity. **a**, PRPS1 WT or *O*-GlcNAcylation-deficient mutants purified from HEK293T cells were subject to PRPS1 enzymatic activity assays. Intensities of FLAG-PRPS1 immunoblots were used to quantify PRPS1 activity (*n* = 3). **b**, PRPS1 WT or 2A plasmids were reintroduced into PRPS1-depleted H1299 cells. Cells were incubated in medium containing ^13^C_6_-glucose. LC–MS/MS analysis was performed to measure ^13^C_6_-labeled intracellular nucleotide monophosphates (*n* = 3). **c**, Human PRPS1 dimer structure modeling (PDB ID: 2H06). **d**, The indicated FLAG-PRPS1 plasmids were introduced into HEK293T cells. FLAG-PRPS1 was eluted in size-exclusion fractions on the same column in identical settings. Indicated fractions of cell lysates were used for immunoblot analyses. Due to the relatively low protein expression levels of PRPS1 hexamers, fractions 24–28 and fractions 34–44 were collected and combined to conduct the *O*-GlcNAcylation analysis for PRPS1 hexamers and monomers, respectively, using the chemoenzymatic labeling method (right) (*n* = 3). **e**, Oligomerization analysis of PRPS1 WT and 2A using the crosslinking method in HEK293T cells pre-treated with or without 2-μm thiamet-G for 3 h (*n* = 3). **f**–**i**, ADP and GDP feedback inhibition on PRPS1 enzymatic activity in HEK293T cells. Exogenous PRPS1 was purified from control or OGT knockdown cells (**f**,**g**) and PRPS1 WT or 2A mutant transfected cells (**h**,**i**) and subjected to enzymatic activity assays (*n* = 5). **j**, Structural analysis of *O*-GlcNAcylation-associated residues and ADP/GDP binding sites in human PRPS1 dimer (PDB ID: 8DBE, which contains ADP in both allosteric and catalytic sites). **k**, LC–MS/MS analysis of NAD production in WT or 2A PRPS1 rescued PRPS1-depleted H1299 cells. The incorporation of ^13^C_6_-glucose into NAD was analyzed (*n* = 3). **l**, The PRPS1 WT and 2A plasmids were reintroduced in PRPS1-depleted H1299 cells. Cells were incubated with or without inosine (I), adenosine (A), guanosine (G), uridine (U), cytidine (C) and NAD. The proliferation rates were calculated (*n* = 5). Each error bar represents mean ± s.e.m. A two-tailed Student’s *t*-test was employed for statistical evaluation. IP, immunoprecipitation. Source data

In vitro enzymatic experiments demonstrated that both T166 *O*-GlcNAcylation and S83 *O*-GlcNAcylation promoted PRPS1 enzyme activity, and T166 played a more important role than S83 (Fig. 3a). PRPS1 S83A/T166A mutant (2A mutant) showed about 60% less enzymatic activity than PRPS1 wild-type (WT) (Fig. 3a). Thus, S83/T166 *O*-GlcNAcylation activates PRPS1. Further supporting these results, PRPS1 2A mutant cells showed decreased de novo synthesis of purine intermediate (IMP/AMP/GMP) as well as pyrimidine intermediate (UMP/CMP) levels (Fig. 3b).

**Extended Data Fig. 3:**
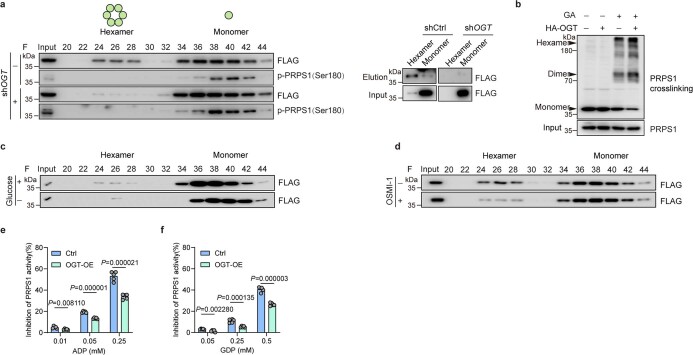
*O*-GlcNAcylation promotes PRPS1 hexamer formation and PRPS1 activity. **a**, The indicated shRNAs and FLAG-PRPS1 plasmids were introduced into HEK293T. FLAG-PRPS1 was eluted in size-exclusion fractions on the same column in identical settings. Indicated fractions of cell lysates were used for immunoblot analyses. Due to the relatively low protein expression levels of PRPS1 hexamers, Fractions 24-28 and Fractions 34-44 were collected and combined to conduct the *O*-GlcNAcylation analysis for PRPS1 hexamers and monomers respectively using the chemoenzymatic labeling method (right panel). (n = 3). **b**, The HA-vector or HA-OGT plasmids were introduced into HEK293T cells. 0.025% glutaraldehyde (GA) was added into indicated cells lysates to cross link proteins. Equal amounts of input and cross-linked protein samples were blotted with the indicated antibody. (n = 3). **c**,**d**, The FLAG-PRPS1 plasmids were introduced into HEK293T cells. The cells were cultured with or without glucose for 24 h (**c**) or treated with or with no 50 µM OSMI-1 for 24 h (**d**). FLAG-PRPS1 was eluted in fractions on the same size-exclusion column in identical settings. The indicated fractions of the cell lysates were utilized in the immunoblot analyses. (n = 3). **e**,**f**, HEK293T cells were transfected with or without HA-OGT plasmids. All the cells were transfected with FLAG-PRPS1 plasmids. FLAG-PRPS1 was purified and then subjected to the ADP (**e**) and GDP (**f**) feedback inhibition of PRPS1 activity reaction with indicated concentrations of ADP and GDP. (n = 5). Each error bar represents mean±SEM. Statistical analysis was done using two-tailed Student’s t-tests. Source data

PRPS1 exists in monomer or hexamer forms in cells, and the latter show much stronger activity. Structural analyses showed that T166 residue is situated in proximity to the interface of PRPS1 hexamer subunits (Fig. 3c). Based on these observations, we hypothesized that OGT-mediated *O*-GlcNAcylation of PRPS1 may regulate the hexamer formation of the protein. To investigate this hypothesis, we performed size-exclusion chromatography analyses as previously described^11^ as well as oligomerization assays using the crosslinking reagent. Consistent with the previous reports^8,11^, PRPS1 was present in both monomer and hexamer forms in cells (Fig. 3d). Knockdown of OGT promoted PRPS1 conversion from hexamers to monomers (Extended Data Fig. 3a). OGT overexpression promoted PRPS1 conversion from monomers to hexamers (Extended Data Fig. 3b). Glucose starvation or OGT inhibitor treatment showed similar effects (Extended Data Fig. 3c,d). Furthermore, the PRPS1 2A mutant exhibited much less hexamer formation (Fig. 3d,e). PRPS1 monomer showed low *O*-GlcNAcylation and high S180 phosphorylation. PRPS1 hexamer showed high *O*-GlcNAcylation and low S180 phosphorylation (Fig. 3d and Extended Data Fig. 3a). These findings reveal that OGT-mediated *O*-GlcNAcylation of PRPS1 promotes PRPS1 hexamer formation.

ADP and GDP inhibit PRPS1 activity through a feedback mechanism^8,9,17^. To assess the impact of byproduct feedback inhibition on PRPS1 activity, we conducted an enzymatic assay with increasing amounts of ADP or GDP. Knockdown or overexpression of OGT promoted or relieved ADP/GDP inhibition on PRPS1 activity, respectively (Fig. 3f,g and Extended Data Fig. 3e,f). The PRPS1 2A mutation also showed a much stronger feedback inhibitory effect (Fig. 3h,i), suggesting that reduced feedback inhibition may be the mechanism behind the activation of PRPS1 via *O*-GlcNAcylation. Structure analysis showed that T166 is close to ADP/GDP binding sites, and S83 is not proximal to ADP/GDP binding sites (Fig. 3j). T166 is located in the catalytic domain, adjacent to the outer surface of the hexamer, and is not directly exposed in the available structures. Insertion of *O*-GlcNAcylation at this site induces both local and global conformational changes. Given that it is connected to the helix that links the allosteric domain, which is further connected to the β-strand that binds ADP/GDP, we speculated that the opening of ADP/GDP binding site would relieve ADP/GDP-mediated feedback inhibition (Fig. 3j). Structure analysis also revealed that S83 is located in the allosteric domain and positioned at the inner circle of the hexamer, without any interactions with other protomers or ligands. However, *O*-GlcNAcylation at this site functions as a molecular adhesive, contributing to the stabilization of the hexamer (Extended Data Fig. 2l). Perhaps OGT-mediated T166 and/or S83 *O*-GlcNAcylation induced PRPS1 local and/or global conformational change, respectively, weakening or blocking ADP/GDP binding and relieving ADP/GDP-mediated feedback inhibition.

Similarly to OGT knockdown cells (Fig. 1g), PRPS1 2A mutant cells also showed lower production of ^13^C-labeled NAD (Fig. 3k). These findings show that *O*-GlcNAcylation of PRPS1 mediated by OGT enhances NAD biosynthesis. Both NAD and nucleotides are important for cancer cell proliferation. To distinguish whether the phenotype stemmed from the lack of nucleotides or the lack of NAD, we replenished NAD, nucleotides or NAD plus nucleotides to the medium of PRPS1 *O*-GlcNAcylation-deficient cells and examined cell proliferation. As shown in Fig. 3l, replenishment of NAD or nucleotides could partially rescue cancer cell proliferation, and replenishment of both NAD and nucleotides almost fully rescued cancer cell proliferation. These results indicate that both NAD and nucleotides are essential for the phenotype of PRPS1.

### PRPS1 *O*-GlcNAcylation inhibits its phosphorylation by AMPK

**Fig. 4:**
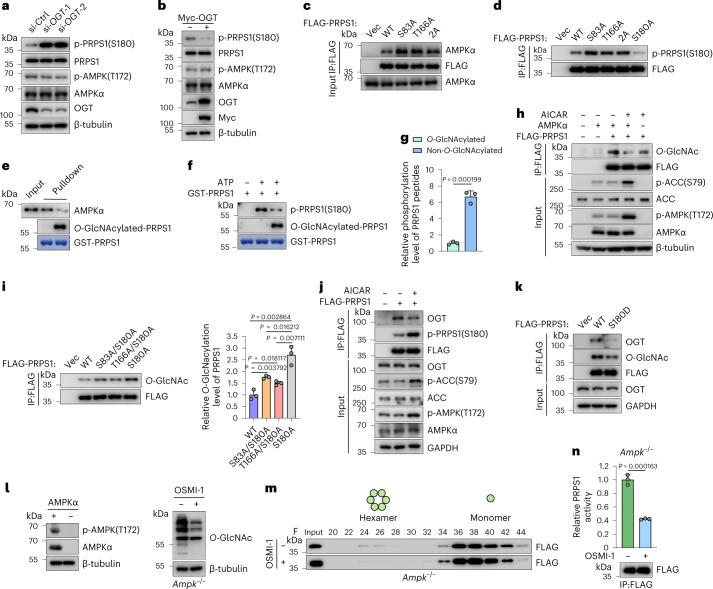
PRPS1 *O*-GlcNAcylation inhibits its phosphorylation by AMPK. **a**,**b**, The indicated siRNAs were introduced into H1299 cells (**a**); the Myc-vector or Myc-OGT plasmids were introduced into H1299 cells (**b**). Immunoblot analyses were conducted with indicated antibodies (*n* = 4). **c**,**d**, HEK293T cells were transfected by indicated plasmids. Subsequent immunoprecipitation (IP) and immunoblotting procedures were carried out to examine interaction with AMPK (**c**) (*n* = 3) and S180 phosphorylation levels (**d**) (*n* = 4) of PRPS1 WT, S83A, T166A, 2A or S180A. **e**,**f**, The recombinant *O*-GlcNAcylated and non-*O*-GlcNAcylated GST-PRPS1 proteins obtained from the in vitro *O*-GlcNAcylation assays were subjected to in vitro pulldown of AMPK (**e**) or AMPK in vitro kinase assays (**f**) (*n* = 3). **g**, The stoichiometry analysis of S180 phosphorylation on *O*-GlcNAcylated or non-*O*-GlcNAcylated peptides (aa 160–185) by AMPK through MS (*n* = 3). **h**, The FLAG-PRPS1 plasmids were introduced into *Ampkα1/α2* WT or depleted MEFs. Indicated cells were treated with or without 2 mM AICAR for 1 h. IP and immunoblot analyses were performed with anti-FLAG beads and indicated antibodies (*n* = 2). **i**, HEK293T cells were transfected with indicated plasmids. IP and immunoblot analyses were performed with anti-FLAG beads and indicated antibodies. Quantification of PRPS1 *O*-GlcNAcylation levels is shown on the right (*n* = 3). **j**,**k**, HEK293T cells were transfected with indicated plasmids and treated with or without 2 mM AICAR for 1 h (**j**); HEK293T cells were transfected with indicated plasmids, including PRPS1 S180 phosphorylation mimic mutant (S180D) (**k**). IP and immunoblot analyses were conducted with anti-FLAG beads and indicated antibodies (*n* = 3). **l**, Immunoblot analyses were performed with the indicated antibodies in *Ampk*α1/α2 WT or depleted MEFs (left); the *Ampkα1/α2-*depleted MEFs were treated with or without 50 µM OSMI-1 for 24 h (right) (*n* = 3). **m**,**n**, The FLAG-PRPS1 plasmids were introduced into *Ampkα1/α2*-depleted MEFs. Cells were treated with or without 50 µM OSMI-1 for 24 h; FLAG-PRPS1 was eluted in fractions on the size-exclusion column in the same settings. Indicated samples were subjected to immunoblot analyses (**m**). FLAG-PRPS1 was purified and subjected to PRPS1 enzymatic activity assays (**n**). FLAG-PRPS1 protein levels in reactions were examined by western blot (lower panel) (*n* = 3). Every error bar signifies mean ± s.e.m. A two-tailed Student’s *t*-test was employed for statistical evaluation. Source data

**Extended Data Fig. 4:**
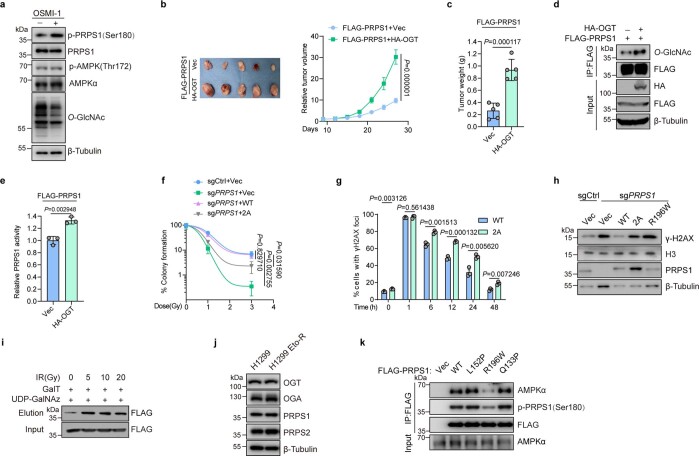
PRPS1 *O*-GlcNAcylation promotes tumor growth. **a**, H1299 cells were treated with or without 50 µM OSMI-1 for 24h. Immunoblot analyses were conducted with indicated antibodies. (n = 3). **b**-**e**, The indicated plasmids were introduced into H1299 cells. The cells were subcutaneously injected into mice flank regions. Xenograft volumes were measured on the indicated days (**b**). By the end of the experiment, tumors were harvested and weighed after euthanization of mice (n = 5) (**c**). The lysate of homogenized xenografts was subjected to immunoprecipitation and immunoblotting with anti-FLAG agarose beads and indicated antibodies (**d**). FLAG-PRPS1 was purified from the xenograft lysates and subjected to PRPS1 enzymatic activity assays (n = 3) (**e**). **f**, The indicated PRPS1 plasmids were reintroduced in PRPS1-deleted H1299 cells. The cells were irradiated with a series of doses of radiation. The colony forming efficiencies were analyzed. (n = 3). **g**, The PRPS1 WT/2A plasmids were reintroduced in PRPS1-deleted H1299 cells. γ-H2AX focus positive cell ratios at the indicated time points after 2Gy irradiation were analyzed. (n = 3). **h**, The indicated PRPS1 plasmids were reintroduced in PRPS1-deleted H1299 cells. Cells were exposed to 50 µM etoposide for 2 h. Immunoblotting was conducted with indicated antibodies. (n = 2). **i**, H1299 cells were exposed to indicated doses of radiation. PRPS1 *O*-GlcNAcylation was analyzed using the chemoenzymatic labeling method. Immunoblot analyses were conducted with the indicated antibodies. (n = 3). **j**, Immunoblotting was conducted with indicated antibodies in H1299 parental and etoposide-resistant (Eto-R) cells. (n = 3). **k**, The indicated plasmids were introduced into HEK293T cells. Immunoprecipitation and immunoblot analyses were performed using anti-FLAG agarose beads and indicated antibodies. (n = 3). Each error bar represents mean±SEM. Two-tailed Student’s t-tests were employed to do statistical analysis. Source data

PRPS1 is directly phosphorylated at S180 by the energy sensor AMPK, resulting in the PRPS1 transformation from hexamers to monomers as well as subsequent PRPS1 activity inhibition^11^. Furthermore, PRPS1 monomers showed low *O*-GlcNAcylation and high S180 phosphorylation. PRPS1 hexamers showed high *O*-GlcNAcylation and low S180 phosphorylation (Fig. 3d and Extended Data Fig. 3a). Given these observations, we sought to explore the possibility of crosstalk between OGT-mediated PRPS1 *O*-GlcNAcylation and AMPK-dependent phosphorylation. Knockdown of OGT or OGT inhibitor treatment substantially increased PRPS1 S180 phosphorylation but had no effect on AMPK activation (AMPK T172 phosphorylation) (Fig. 4a and Extended Data Fig. 4a), and overexpression of OGT decreased PRPS1 S180 phosphorylation (Fig. 4b) in H1299 cells. These findings demonstrated that OGT-mediated *O*-GlcNAcylation of PRPS1 blocked AMPK-mediated phosphorylation of PRPS1. Consistently, the PRPS1 *O*-GlcNAcylation-deficient mutants (S83A, T166A and 2A) boosted AMPK binding and showed higher phosphorylation levels in HEK293T cells (Fig. 4c,d). Moreover, we produced *O*-GlcNAcylated PRPS1 protein in vitro and confirmed that *O*-GlcNAcylated PRPS1 bound less AMPK and *O*-GlcNAcylation blocked AMPK-mediated PRPS1 phosphorylation in vitro (Fig. 4e,f). We also synthesized *O*-GlcNAcylated or unmodified peptides of PRPS1 from aa 160–185. By employing AMPK in vitro kinase assays and MS, we further confirmed that T166 *O*-GlcNAcylation blocked AMPK-mediated S180 phosphorylation (Fig. 4g).

On the other hand, AICAR (an AMPK activator) treatment considerably inhibited PRPS1 *O*-GlcNAcylation in *Ampkα1/α2* WT mouse embryonic fibroblasts (MEFs) (Fig. 4h) . This depends (at least partially) on AMPK, because AICAR treatment had no considerable effect on PRPS1 *O*-GlcNAcylation in *Ampkα1/α2*-depleted MEFs (Fig. 4h). The PRPS1 S180 phosphorylation-deficient mutant (S180A) showed increased *O*-GlcNAcylation level (Fig. 4i). We also generated S83A/S180A and T166A/S180A double mutants and found that S180A increased both S83 *O*-GlcNAcylation (T166A/S180A mutant) and T166 *O*-GlcNAcylation (S83A/S180A mutant) (Fig. 4i). Moreover, PRPS1 S180 phosphorylation induced by AICAR or S180D mutant markedly decreased PRPS1 binding to OGT (Fig. 4j,k), resulting in decreased PRPS1 *O*-GlcNAcylation at S83 and T166. PRPS1 binds OGT through its C-terminal region (aa 148–318) (Extended Data Fig. 2b). Perhaps AMPK-mediated phosphorylation of PRPS1 at S180 induced PRPS1 conformational change, which blocked PRPS1–OGT interaction. All these findings show that OGT-mediated *O*-GlcNAcylation of PRPS1 and AMPK-mediated PRPS1 S180 phosphorylation inhibited each other, perhaps through inducing global conformational change and blocking protein binding. We also found that AMPK is not essential for *O*-GlcNAcylation on PRPS1, because OGT inhibitor treatment still promoted PRPS1 conversion from hexamers to monomers (Fig. 4l,m) and inhibited PRPS1 activity in *Ampkα1/α2*-depleted MEFs (Fig. 4n).

### PRPS1 *O*-GlcNAcylation promotes tumor growth

**Fig. 5:**
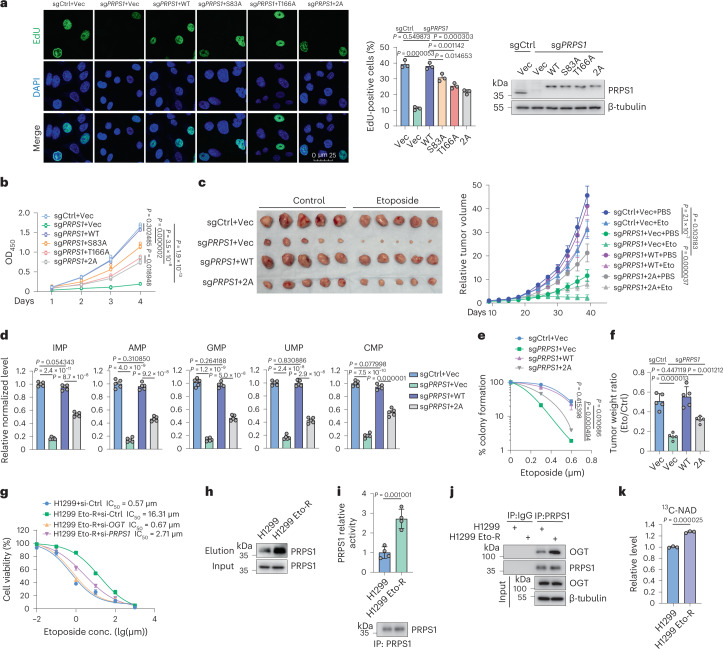
PRPS1 *O*-GlcNAcylation promotes tumor growth. **a**,**b**, The indicated plasmids were reintroduced into PRPS1-deleted H1299 cells. Cells were labeled with 10 µM EdU for 30 min, and EdU-positive cells were examined by immunostaining. The quantification is shown in the middle panel. Protein levels were examined by western blot in the right panel (**a**) (*n* = 3). Cell growth was analyzed by measuring OD_450_ in cell viability assays. The box-and-whisker plots show all the points from the minimum to maximum values (**b**) (*n* = 5). **c**,**d**, H1299 cells were transfected with the indicated constructs and injected subcutaneously into flank regions of nude mice. Mice were treated with PBS or etoposide (20 mg kg^−1^, twice a week for 3 weeks), and xenograft volumes were measured on the indicated days (**c**). At the end of the experiment, mice were euthanized, and tumors were weighed. The xenografts were subjected to label-free LC–MS/MS analysis for nucleotide monophosphate levels (**d**) (*n* = 5). **e**, The indicated H1299 cells were seeded and treated with indicated concentrations of etoposide. The colony-forming efficiencies were analyzed (*n* = 3). **f**, The tumor weight ratios between control and etoposide-treated mice from **c** were calculated (*n* = 5). **g**, The indicated siRNAs were introduced into H1299 parental and Eto-R cells. Cells were exposed to indicated concentrations of etoposide for 48 h, and cell viability assays were performed (*n* = 4). The IC_50_ of etoposide was calculated for each group. **h**, The PRPS1 *O*-GlcNAcylation levels of H1299 parental and Eto-R cells were analyzed using the chemoenzymatic labeling method. Immunoblotting was conducted with the anti-PRPS1 antibody (*n* = 3). **i**, Endogenous PRPS1 purified from H1299 parental and Eto-R cells was subjected to PRPS1 enzymatic activity assays. Intensities of immunoblots were used to quantify PRPS1 activity (*n* = 4). **j**, Immunoprecipitation (IP) and immunoblotting were conducted with indicated antibodies in H1299 parental and Eto-R cells with indicated antibodies (*n* = 3). **k**, H1299 parental and Eto-R cells were incubated in the medium containing ^13^C_6_-glucose. LC–MS/MS was performed to measure the level of ^13^C_6_-labeled NAD (*n* = 3). Each error bar represents mean ± s.e.m unless indicated otherwise. A two-tailed Student’s *t*-test was employed for statistical analysis. conc., concentration. Source data

To assess physiological significance about PRPS1 *O*-GlcNAcylation, we investigated its impacts on cancer cell proliferation and growth. *PRPS1* knockout in H1299 cells reduced EdU incorporation rate and cell proliferation, which were restored by the introduction of WT PRPS1 but not by the *O*-GlcNAcylation-deficient mutants (Fig. 5a,b). Both PRPS1 S83 *O*-GlcNAcylation and T166 *O*-GlcNAcylation promoted EdU incorporation and cell proliferation, and T166 played a more important role than S83 (Fig. 5a,b).

To examine the applicability of these findings in vivo, we performed animal studies by implanting H1299 cells into nude mice. As shown in Extended Data Fig. 4b–e, overexpressed OGT substantially promoted tumor growth in vivo, with elevated PRPS1 *O*-GlcNAcylation and activity. Moreover, PRPS1 knockdown substantially reduced tumor growth (Fig. 5c). The introduction of PRPS1 WT rescued tumor growth, whereas the PRPS1 2A mutant failed to produce the same effect. (Fig. 5c), indicating that PRPS1 *O*-GlcNAcylation promotes tumor growth. We also analyzed the nucleotide levels in the tumor tissues. As shown in Fig. 5d, PRPS1 *O*-GlcNAcylation promotes nucleotide synthesis in xenografts.

Elevated nucleotide synthesis confers cancer cell resistance to chemoradiotherapy^38,39^. Similarly to PRPS1 knockdown, the PRPS1 2A mutant also sensitized H1299 cells to etoposide and irradiation (Fig. 5e and Extended Data Fig. 4f). The PRPS1 2A mutant affected DNA repair kinetics and genomic stability (Extended Data Fig. 4g,h), and DNA damage induced PRPS1 *O*-GlcNAcylation (Extended Data Fig. 4i). Consistently, PRPS1 2A mutant tumors were more sensitive to etoposide than PRPS1 WT tumors, just as in the PRPS1-deficient group (Fig. 5c,f), indicating that PRPS1 *O*-GlcNAcylation confers lung cancer cell resistance to chemoradiotherapy.

Next, we generated etoposide-resistant lung cancer cells using procedures described previously^40^. Cell viability analyses revealed that the half-maximal inhibitory concentration (IC_50_) of etoposide for etoposide-resistant (Eto-R) H1299 cells increased by 30-fold (16.31 μM to 0.57 μM) (Fig. 5g). Both PRPS1 *O*-GlcNAcylation and activity were increased in Eto-R cells (Fig. 5h,i). However, OGT, OGA and PRPS1 protein levels did not change considerably (Extended Data Fig. 4j). Interactions between PRPS1 and OGT substantially increased in Eto-R cells, partially explaining the increased PRPS1 *O*-GlcNAcylation and activity in those cells (Fig. 5j). Furthermore, OGT or PRPS1 knockdown resensitized Eto-R cells to etoposide (Fig. 5g), indicating that elevated PRPS1 activity is at least one cause of etoposide resistance. H1299 Eto-R cells also produced more NAD than H1299 parental cells (Fig. 5k), which would facilitate therapy resistance of cancer cells as well.

**Extended Data Fig. 5:**
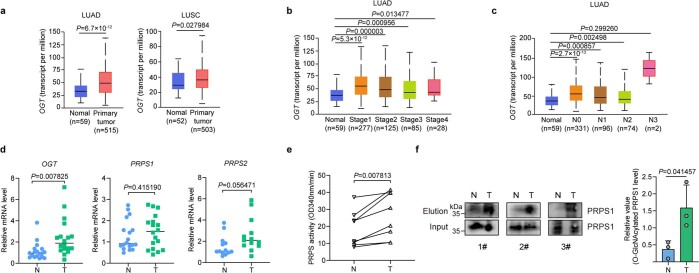
OGT-mediated PRPS1 *O*-GlcNAcylation is associated with tumorigenesis and metastasis in lung cancer patients. **a**-**c**, *OGT* mRNA levels in lung cancer and normal tissues, and the correlation with lung cancer malignancy and metastasis based on data analyses through ULCAN platform. Relative *OGT* mRNA levels in subgroups of lung adenocarcinoma and lung squamous cell carcinoma samples and in normal tissues (**a**); Relative *OGT* mRNA levels in the normal samples and patient samples of Stages 1-4 lung adenocarcinoma (**b**); Relative *OGT* mRNA levels according to nodal metastasis status in patients with lung adenocarcinoma (**c**). The box-whisker plots show interquartile ranges, namely minimum, first quartile, median, third quartile, and maximum values; Two-tailed Welch’s T-test. **d**, *OGT*, *PRPS1*, and *PRPS2* mRNA levels in normal adjacent tissue (N) and tumor tissue (T) from lung cancer patients. Each error bar represents mean±SEM. A two-tailed Student’s t-test was employed for statistical analysis. **e**, PRPS enzymatic activity in lung tumor tissue (T) compared with normal adjacent tissues (N) from lung cancer patients (n = 8). Two-tailed Wilcoxon test was performed for the paired samples. **f**, PRPS1 *O*-GlcNAcylation analysis in lung tumor tissue (T) and normal adjacent tissues (N) using chemoenzymatic labeling method. *O*-GlcNAcylated PRPS1 was blotted in elution and total PRPS1 in input (left panel); PRPS1 *O*-GlcNAcylation levels were calculated (right panel). (n = 3) Each error bar represents mean±SEM. Two-tailed Student’s t-tests were utilized for statistical analysis. Source data

To assess the clinical significance of OGT-mediated PRPS1 *O*-GlcNAcylation, we conducted a data analysis using the ULCAN platform to determine OGT levels in lung tumor tissue^41^. As shown in Extended Data Fig. 5a–c, we observed enhanced mRNA level of *OGT* in cases of lung cancer and its positive correlation with both malignancy and metastasis. We also measured OGT and PRPS1 *O*-GlcNAcylation levels in fresh frozen samples of lung cancer tissues obtained during surgery. Consistently, *OGT* mRNA level was higher in lung tumor tissue than that in normal lung tissue (Extended Data Fig. 5d). *PRPS1/2* mRNA levels did not change considerably (Extended Data Fig. 5d). Notably, PRPS activity and PRPS1 *O*-GlcNAcylation were much higher in tumor tissues (Extended Data Fig. 5e,f), implying that PRPS1 *O*-GlcNAcylation is involved in lung cancer tumorigenesis.

### R196W decreases PRPS1 *O*-GlcNAcylation and activity

**Fig. 6:**
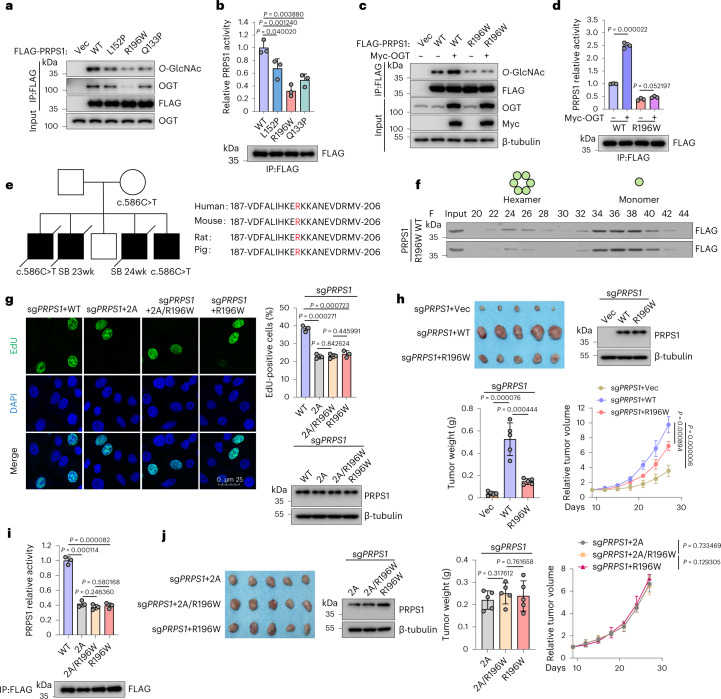
R196W decreases PRPS1 *O*-GlcNAcylation and activity. **a**,**b**, HEK293T cells were transfected by indicated plasmids. Interactions between OGT and WT/mutant FLAG-PRPS1 (L152P, R196W and Q133P) were analyzed by co-immunoprecipitation (IP). PRPS1 *O*-GlcNAcylation in each group was examined (**a**) (*n* = 3). PRPS1 enzymatic activity from **a** is shown (**b**) (*n* = 3). **c**,**d**, Myc-OGT was transfected into WT or R196W mutant HEK293T cells, and PRPS1 *O*-GlcNAcylation level (**c**) and enzymatic activity (**d**) were examined (*n* = 3). **e**, PRPS1 R196W mutation inheritance and family phenotype and conservation of PRPS1 R196 residue across species. The stillbirth (SB) statuses with the gestational week are indicated in the pedigree chart. **f**, WT or PRPS1 R196W mutant from HEK293T cells was eluted in fractions on the size-exclusion column in the same settings. Indicated fraction samples were used for immunoblotting (*n* = 3). **g**, WT or PRPS1 R196W plasmids were replenished into PRPS1 knockout H1299 cells. EdU incorporation assays were performed as in Fig. 5a. PRPS1 protein levels are shown in the lower-right panel (*n* = 3). **h**, The indicated cells were implanted into mice by subcutaneous injection. Xenograft volumes were measured on the indicated days. By the end of the experiment, tumors were harvested and weighed and subjected to immunoblotting after euthanization of mice (*n* = 5). **i**, WT or mutated FLAG-PRPS1 was overexpressed and immunoprecipitated from HEK293T cells and subjected to PRPS1 enzymatic activity assays (*n* = 3). PRPS1 protein level in each group is shown in the bottom panel. **j**, The indicated H1299 cells were implanted into nude mice by subcutaneous injection. Xenograft volumes were measured on the indicated days. By the end of the experiment, tumors were harvested and weighed and subjected to immunoblotting after euthanization of mice (*n* = 5). Every error bar signifies mean ± s.e.m. Two-tailed Student’s *t*-tests were employed for statistical evaluation. wk, weeks. Source data

PRPS1 gene mutations have been implicated in several diseases, such as Arts syndrome, an X-chromosome-linked genetic disorder distinguished by cognitive impairment, delayed motor development and hearing impairment^19,20,42–44^. The presence of PRPS1 loss-of-function mutations in patients with this disease is thought to contribute to deficient purine biosynthesis proved by the absence of detectable urine hypoxanthine and decreased levels of uric acid in serum^18,20,44^. We generated Arts-syndrome-associated PRPS1 mutations. The R196W mutant showed low PRPS1 *O*-GlcNAcylation modification and less activity (Fig. 6a,b). We further examined R196W–OGT interaction and found that R196W mutant considerably decreased PRPS1–OGT interaction (Fig. 6a). Perhaps the weak binding between R196W and OGT could not result in enough *O*-GlcNAcylation, and activation of PRPS1, even overexpressed of OGT, had an insignificant impact on *O*-GlcNAcylation and activation of R196W (Fig. 6c,d). Interestingly, R196W mutant also decreased AMPK binding and S180 phosphorylation (Extended Data Fig. 4k). We speculate that R196 is important for PRPS1 structure. R196W induces PRPS1 conformational change and weakens PRPS1 binding to other proteins, such as OGT and AMPK, resulting in decreased both *O*-GlcNAcylation and phosphorylation. This result also further supports that *O*-GlcNAcylation is essential for PRPS1 enzyme activity.

R196W is an evolutionarily conserved germline mutation in Arts syndrome that was found in the PRPS1 pedigree^18^ (Fig. 6e). As expected, the R196W mutant converted PRPS1 from a hexamer to a monomer structure (Fig. 6f). Furthermore, structural analyses revealed that the PRPS1 R196 residue is situated in close proximity to the subunit interface of the hexamers (Fig. 3c). The R196W mutant also inhibited cell proliferation (Fig. 6g) and tumor cell growth in vivo (Fig. 6h) and had much lower PRPS1 activity (Fig. 6i). We also generated an S83A/T166A/R196W triple mutant. As shown in Fig. 6g,i,j, the triple mutant showed functions similar to the 2A mutant or the R196W mutant, implying that the R196W mutant acts, at least in part, through PRPS1 *O*-GlcNAcylation.

## Discussion

**Extended Data Fig. 6:**
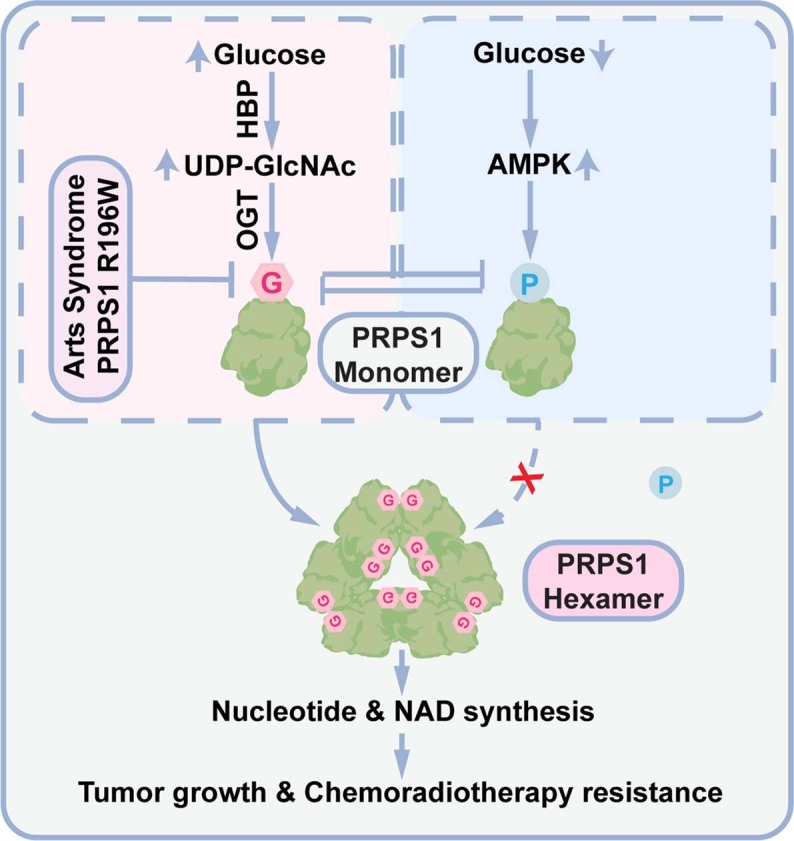
Schematic model of how OGT-mediated nucleotides and NAD synthesis under metabolic stress regulate tumor growth and chemoradiotherapy resistance. In the presence of sufficient nutrients, OGT catalyzes the transfer of UDP-GlcNAc to PRPS1, promoting its hexamer assembly and functional activity. This process enhances *de novo* nucleotide synthesis and NAD production, thereby accelerating tumor growth and resistance to chemoradiotherapy. A mutation associated with Arts Syndrome, PRPS1 R196W, exhibits decreased *O*-GlcNAcylation levels and activity of PRPS1. Under conditions of energy stress, such as glucose deprivation, AMPK phosphorylates PRPS1, leading to a reduction in its hexamer assembly and activity. The OGT-mediated *O*-GlcNAcylation of PRPS1 and AMPK-mediated phosphorylation antagonize each other. The *O*-GlcNAcylation of PRPS1 at S83 and T166 by OGT results in structural alteration, reducing the binding and phosphorylation capacity of AMPK. In contrast, AMPK-mediated phosphorylation of PRPS1 at S180 triggers a conformational change, inhibiting the PRPS1-OGT interaction and lowering PRPS1 *O*-GlcNAcylation. The intricate interplay between OGT and AMPK pathways, responding to distinct nutrient signals, establishes a complex regulatory network for PRPS1.

The HBP serves as a crucial metabolic switch in cells that regulates glucose and lipid metabolism in reaction to changes of intracellular energy levels and nutrient availability^30,45^. Cancer cells usually grow quickly and require more glucose than normal cells to reprogram their metabolism and, thus, support rapid cell proliferation. Here we demonstrated that glucose-induced PRPS1 *O*-GlcNAcylation promotes de novo nucleotide synthesis and NAD production to support tumor growth. PRPS1 O-GlcNAcylation converted PRPS1 monomers to hexamers, relieved nucleotide synthesis products (including ADP and GDP) mediated feedback inhibition, and promoted activity of PRPS1 and synthesis of nucleotides and NAD (Extended Data Fig. 6).

To sustain homeostasis, dividing cells need to synthesize enough nucleotides to catch up with the cell division rate^4^. Thus, de novo nucleotide synthesis is strictly regulated at different levels in cancer. PRPS1 serves as a crucial enzyme in de novo nucleotide synthesis networks. The energy sensor AMPK phosphorylates PRPS1 at S180 to inhibit nucleotide biosynthesis^11^. Here we show that OGT-mediated S83 and T166 *O*-GlcNAcylation promoted PRPS1 activation, de novo nucleotide synthesis and NAD production. Both NAD and nucleotides are essential for PRPS1 functions in cancer cell proliferation (Fig. 3n). The two different post-translational modifications antagonized each other. Mechanistically, AMPK-mediated phosphorylation of PRPS1 at S180 induced PRPS1 conformational change, which blocked PRPS1–OGT interaction, resulting in decreased PRPS1 *O*-GlcNAcylation. Vice versa, OGT-mediated PRPS1 *O*-GlcNAcylation also induced PRPS1 structural alteration, which resulted in decreased AMPK binding and phosphorylation. The AMPK and *O*-GlcNAc pathways represent the principal pathways responding to nutrients that are regulated by cellular energy and nutrient availability^26,46^. These pathways are highly interconnected, although they integrate nutrient information in distinct ways. AMPK monitors the ratio of AMP/ATP, whereas glucose, acetyl-coenzyme A and glutamine are involved in HBP activation and protein *O*-GlcNAcylation^47,48^. Thus, crosstalk between OGT and AMPK in de novo nucleotide synthesis allows cells to sense a variety of environmental cues to control de novo nucleotide synthesis. However, OGT still regulates PRPS1 activity and de novo nucleotide synthesis in AMPK-deficient MEFs (Fig. 4m–o). That said, inhibition of AMPK-mediated PRPS1 phosphorylation is not essential for PRPS1 activation by OGT, indicating the complexity and importance of OGT-PRPS1 cascade to sense environmental cues and regulate de novo nucleotide synthesis.

PRPS1 mutations have been linked to enhanced DNA damage response and chemoradiotherapy resistance^38,49^. Consistent with these findings, we report here that elevated *O*-GlcNAcylation caused activation of PRPS1, which is one cause of resistance to etoposide and irradiation treatments in lung cancer. Thus, reducing PRPS1 activity by silencing OGT could resensitize lung cancer cells to chemoradiotherapy. Our findings suggest that PRPS1 *O*-GlcNAcylation could be used as a biomarker to predict etoposide or radiation resistance and as a treatment target in patients with lung cancer. It is expected that the development of T166 and S83 site-specific *O*-GlcNAcylation antibodies will open up new avenues of PRPS1 *O*-GlcNAcylation in the prediction of clinical outcome.

PRPS1 mutations have been identified as the causative factor of Arts syndrome, which is characterized by cognitive impairment, hearing impairment, optic atrophy, early-onset hypotonia, ataxia and delayed motor development^18–20,42,43,50^. Despite these correlations, the exact mechanisms by which these mutations lead to the clinical phenotype are not yet fully understood. We found that Arts-syndrome-associated PRPS1 R196W mutation decreased OGT binding, PRPS1 *O*-GlcNAcylation and activity. R196W also markedly decreased AMPK binding and PRPS1 S180 phosphorylation. Perhaps R196W mutation induced PRPS1 conformational change and weakened PRPS1 binding to other proteins, such as OGT and AMPK, resulting in both decreased *O*-GlcNAcylation and phosphorylation. The R196W mutant partially functions through PRPS1 *O*-GlcNAcylation, which results in impaired nucleotide biosynthesis. Previous reports described undetectable levels of urine hypoxanthine and decreased serum uric acid in patients with Arts syndrome^18,20,44^. Interestingly, a clinical trial showed that replenishing purines and S-adenosylmethionine administration had curative effects against Arts syndrome^44^. Given these findings, it is possible that OGT-mediated PRPS1 *O*-GlcNAcylation may be involved in the etiology of PRPS1 R196W mutation and Arts syndrome.

Collectively, our results highlight a central role for glucose sensor *O*-GlcNAcylation in de novo nucleotide synthesis. Rescuing the homeostasis of PRPS1 activity by controlling OGT-mediated *O*-GlcNAcylation or AMPK-mediated phosphorylation is a potential clinical prevention strategy for PRPS1 dysregulation-associated diseases, such as Arts syndrome, or for tumorigenesis and resistance to chemoradiotherapy in lung cancer.

## Methods

### Mice

Experiments involving animals adhered to a protocol with approval from Institutional Animal Care and Use Committee (IACUC) at Georgetown University. The animals were accommodated in well-ventilated cages with regulated temperature between 20 °C and 26 °C, with humidity levels within the range of 30–70%, with a 12-h light/dark cycle and having unrestricted access to food and water. No more than five mice lived in one cage. Subcutaneous injections of cells were performed in 6–8-week-old male nude mice. This investigation adhered to all applicable ethical guidelines concerning the use of animals in research.

### Cell lines

H1299 cells (American Type Culture Collection (ATCC), CRL-5803) (isolated from the lung of a 43-year-old male patient with carcinoma); A549 cells (ATCC, CCL-185) (isolated from the lung tissue of a 58-year-old male patient with lung cancer); and HEK293T cells (ATCC, CRL-11268) (isolated from human embryo kidney tissue) were purchased from ATCC. *Ampk*^+/+^ and *Ampk*^−/−^ MEF cells were kindly provided by Eduardo Chini (Mayo Clinic). These cells were cultured in DMEM (Cytiva, SH30243.01) supplemented with 10% FBS (HyClone, SH30910.03) and 1% penicillin–streptomycin (Corning, 30-002-CI) unless otherwise indicated. All cells were maintained at 37 °C and 5% CO_2_.

### Microbe strains

The strain of DH5α *Escherichia coli* (New England Biolabs, C2527) was cultured at 37 °C in Luria–Bertani (LB) broth (Fisher BioReagents, BP1427) to amplify plasmids. The strain of BL21 *E. coli* (New England Biolabs, C2987) was cultured at 16 °C in LB broth (Fisher BioReagents, BP1427) with 0.2–0.5 µM isopropyl β-d-1-thiogalactopyranoside (IPTG) (Sigma-Aldrich, I6758) for protein purification.

### Human specimens

Lung tumor samples and adjacent tissues from the same individuals were collected from surgically resected lung tissues at Renmin Hospital of Wuhan University (Supplementary Table 1). The specimens were diagnosed by pathologists. The study’s protocol was approved by the Ethics Committee of Renmin Hospital of Wuhan University. All participants provided informed consent for the study.

### Plasmids, shRNA, siRNA and transfection or infection

*PRPS1* WT, O-GlcNAcylation-deficient mutants (S83/Thr166 **→** Ala: S83A, T166A and 2A), S180 phosphorylation-deficient mutant (S180 **→** Ala: S180A), S180 phosphorylation mimic mutant (S180 **→** Asp: S180D) or disease-associated mutants (Leu152 **→** Pro: L152P; Arg196 **→** Trp: R196W; Gln133 **→** Pro: Q133P) were cloned into pEFF-FLAG or pCDH-FLAG. Cell transfection was performed using polyethyleneimine (PEI) (Polysciences, 23966) or Lipofectamine 3000 (Thermo Fisher Scientific, L3000) in accordance with the manufacturersʼ instructions.

The shRNA oligonucleotides targeting human *OGT* (Supplementary Table 2) were cloned into a pLKO.1 vector (Addgene, 10878). Lentivirus particles were produced to transduce cells to express shRNA or *PRPS1* cDNA (pCDH-FLAG plasmids). The indicated plasmids, PEI (Polysciences, 23966) and packaging vector pMD2.G (Addgene, 12259) and psPAX2 (Addgene, 12260) were used to transfect HEK293T cells (ATCC, CRL11268) to produce lentivirus particles. The medium containing virus was collected and filtered at 48 h and 72 h after transfection. Cells were infected by the virus with 8 µg ml^−1^ polybrene (Millipore, TR1003G). Medium containing 2 µg ml^−1^ puromycin (Sigma-Aldrich, 9620) was used for selection.

Lipofectamine RNAiMAX (Thermo Fisher Scientific, 13778) was used to deliver siRNA oligonucleotides into cells based on the manufacturer’s protocol. The siRNA sequences targeting human *OGT* and *PRPS1* are summarized in Supplementary Table 2.

### Gene deletion by CRISPR–Cas9

The sgRNA oligonucleotide targeting human *PRPS1* (Supplementary Table 2) was cloned into a lentiCRISPRv2 vector according to Feng Zhang’s protocol^51,52^. The sgRNA oligonucleotide targeting human *PRPS2* (Addgene, 78005)^53^ was obtained from Addgene. Lentiviruses were produced as above to infect cells. Puromycin selection was conducted, and single clone cells were picked and amplified to test knockout efficiency by immunoblotting. For the PRPS1 expression rescue study, PRPS1 cDNAs carrying knockout-resistant synonymous mutations (CATGGTACTAGTAGGCGACG) against sg*PRPS1* were introduced into *PRPS1* knockout cells.

### Real-time PCR analysis

Total RNA was extracted using a Total RNA Miniprep Super Kit (BIO BASIC, BS784). RNA was used to do reverse transcription with the iScript Reverse Transcription Supermix (Bio-Rad, 1708840) in preparation for real-time PCR analysis. SYBR Green Supermix (Bio-Rad, 1725270) was used to do real-time PCR with the Bio-Rad CFX96 device. Reactions were run in triplicate using primers targeting human genes (Supplementary Table 2).

### Protein purification, immunoprecipitation and immunoblotting

Cells were harvested and resuspended in NETN buffer supplemented with protease inhibitor cocktail (Roche, 4693159001) or phosphatase inhibitor cocktail (Roche, 4693159001). The cells were lysed by rotation at 4 °C for 10–20 min and then centrifuged at 13,000*g* for 15 min at 4 °C after short sonication. The supernatant was collected, and protein concentrations were quantified with a BCA Protein Assay Kit (Tiangen, PA115).

For immunoprecipitation, the lysate was incubated with indicated primary antibodies and protein A/G agarose beads (Santa Cruz Biotechnology, sc-2003) or with anti-FLAG beads (Sigma-Aldrich, A2220) at 4 °C overnight. Normal IgG (Cell Signaling Technology, 2729) was used as control in endogenous immunoprecipitation. The beads were washed five times with NETN buffer, and the precipitated proteins were subjected to immunoblot analyses or other functional assays.

For immunoblotting, equal amounts of protein were mixed with loading buffer (Bio-Rad, 1610747) and boiled for 5–10 min. The protein samples were loaded and separated in an SDS-PAGE gel, followed by transfer onto a PVDF membrane (Thermo Fisher Scientific, 88518). The membranes were incubated in indicated primary antibody solutions at 4 °C for 12–16 h after being blocked in 5% milk TBST solution. The following primary antibodies were used for immunoblotting: PRPS1 (Proteintech, 15549-1-AP); phospho-PRPS1 (S180) (Thermo Fisher Scientific, PA5-106230); PRPS2 (Novus, NBP1-31435); OGT (Cell Signaling Technology, 24083); OGA (Proteintech, 14711-1-AP); AMPKα (Cell Signaling Technology, 2532); phospho-AMPKα (Thr172) (Cell Signaling Technology, 2535); ACC (Cell Signaling Technology, 3662); phospho-ACC (S79) (Cell Signaling Technology, 3661); β-tubulin (Proteintech, 66240-1-Ig); β-actin (Proteintech, 60008-1-Ig); γ-H2AX (S139) (Cell Signaling Technology, 9718); histone H3 (Millipore, 06-755); *O*-GlcNAc (RL2) (Abcam, ab2739); *O*-GlcNAc (CTD110.6) (Cell Signaling Technology, 9875); *O*-GlcNAc (18B10.C7) (Thermo Fisher Scientific, MA1-038); FLAG (Sigma-Aldrich, F3165); HA (BioLegend, MMS-101P, clone 16B12); HA (Cell Signaling Technology, 3724); and Myc (Santa Cruz Biotechnology, sc-40). The membranes were washed with TBST buffer before secondary antibody (Jackson ImmunoResearch, 115-035-003 and 111-035-003) incubation. The secondary antibodies were washed off, and blots were developed with chemiluminescent substrate (Thermo Fisher Scientific, 34580) by an AI600 imager (GE Healthcare).

### Click-iT *O*-GlcNAc enzymatic labeling and stoichiometric analysis of *O*-GlcNAcylated PRPS1

*O*-GlcNAcylation of PRPS1 using the enzymatic labeling method was carried out as described previously^37,54–56^. In brief, 400 μg of total cell or tissue lysate was labeled according to the Invitrogen Click-iT *O*-GlcNAc enzymatic labeling protocol (Thermo Fisher Scientific, C33368). Enzymatic labeled proteins were conjugated with an alkyne-biotin compound based on Invitrogen Click-iT protein analysis detection kit protocol (Thermo Fisher Scientific, C33372). The biotinylated proteins were precipitated by streptavidin resin (Thermo Fisher Scientific, 20353) and then eluted in a loading buffer containing 20 mM biotin (Sigma-Aldrich, B4501) by boiling. The immunoblot intensities of PRPS1 in elution and in input were measured by ImageJ (National Institutes of Health, https://imagej.nih.gov/ij/) to quantify the PRPS1 *O*-GlcNAcylation level for each sample.

### *O*-GlcNAcylation in vitro assay

The *O*-GlcNAcylation in vitro assay was reported previously^30,33^. In brief, about 2 μg of GST-PRPS1 or GST-PRPS2 protein was incubated with 1 μg of enzymatic His-OGT domain (aa 323–1,041) in 100 μl of 50 mM Tris-HCl (pH 7.5) containing 12.5 mM MgCl_2_, 2 mM UDP-GlcNAc and 1 mM dithiothreitol at 37 °C for 2–5 h. The proteins were subjected to immunoblot analyses or other functional assays.

### Cell growth and cell colony formation assays

For cell growth assays, cells were seeded into a 96-well plate. A reagent (Dojindo, CK04-11) was added into each well to measure cell numbers by reading optical density at 450 nm (OD_450_) values using a plate reader based on the protocol from the manufacturer. For cell colony-forming assays, 500–800 cells were seeded in tissue culture plates and grown for 1 week. Cell colonies were fixed in 4% formaldehyde (Sigma-Aldrich, 47606) and then stained with 1% crystal violet (Sigma-Aldrich, C6158) for counting.

For cell proliferation rate analysis after replenishment of IAGUC and NAD, inosine (I4125), adenosine (A4036), guanosine (G6264), uridine (U3003), cytidine (C4654) and β-nicotinamide adenine dinucleotide (N7004) were obtained from Sigma-Aldrich. Cells were seeded in a 96-well plate (3,000 cells per well) and incubated in the medium with or without 0.5 mM inosine, adenosine, guanosine, uridine, cytidine and β-nicotinamide adenine dinucleotide from Sigma-Aldrich for 72 h from day 1 to day 4. The OD_450_ was measured, and the proliferation rate was calculated by dividing the OD_450_ on day 4 by that on day 1 for each group.

### GST pulldown assays

*PRPS1*, *PRPS1* truncated mutants and *OGT* were cloned into a pGEX-4T-2 vector. GST, GST-OGT, GST-PRPS1 and GST-PRPS1 truncated mutant fusion proteins were expressed and purified from *E. coli* BL21 cells (New England Biolabs, C2527) and captured by glutathione sepharose gels (Cytiva, 17075601) and then rotated with HEK293T cell lysate at 4 °C overnight. The recombinant *O*-GlcNAcylated and non-*O*-GlcNAcylated GST-PRPS1 was obtained from the in vitro *O*-GlcNAcylation assays as previously described, and, after being washed three times, the recombinant GST-PRPS1 was then incubated with the purified AMPK (Upstate Biotechnology, 14-840) at 4 °C overnight. The beads were washed five times and boiled in a loading buffer (Bio-Rad, 1610747). The samples were subjected to immunoblot analyses and Coomassie blue (Sigma-Aldrich, B0149) staining.

### Immunostaining experiments

Cells were cultured on glass coverslips in 3.5-cm dishes. After indicated treatments, cells were washed in PBS, fixed in 4% formaldehyde for 15 min, permeabilized in 0.5% Triton X-100 for 5 min and blocked in 5% BSA for 1 h at room temperature. The coverslips were incubated in a diluted γ-H2AX primary antibody (Cell Signaling Technology, 9718) solution for 1 h at room temperature. After being rinsed twice in PBS, the cells were incubated in a diluted secondary antibody (Jackson ImmunoResearch, 111-585-045) solution for 30 min at room temperature. The coverslips (Globe, 1404) were then washed in PBS. A mounting medium with DAPI (Thermo Fisher Scientific, 00-4959-52) was used to mount the coverslips on a glass slide (Globe, 1354). Images were captured by Leica SP8 microscopy, and γ-H2AX focus-positive (≥10 foci per cell) cell ratios were counted and analyzed.

### EdU incorporation assays

A Click-iT EdU Cell Proliferation Kit for Imaging (Thermo Fisher Scientific, C10337) was used to perform EdU incorporation assays according to the manufacturer’s instructions. In brief, cells were seeded on coverslips (Globe, 1404) and labeled in 10 µM EdU medium for 30 min. After fixation and permeabilization, cells were treated with the reaction cocktail mixture for about 30 min in the dark at room temperature. The coverslips were washed twice in PBS and mounted with a mounting medium with DAPI (Thermo Fisher Scientific, 00-4959-52) on a glass slide (Globe, 1354). Images were captured by Leica SP8 microscopy, and EdU-positive cells were counted and analyzed.

### Size-exclusion chromatography

Size-exclusion chromatography was performed using a Superdex 200 Increase 10/300 GL column (GE Healthcare, 28-9909-44) and a GE ÄKTA pure machine. Then, 500 µl of cell lysate was loaded onto the column and fractionated at a flow rate of 0.5 ml min^−1^. A protein standard mix of 15–600 kDa (Supelco, 69385) was used as a calibration marker. The sample fractions were subjected to immunoblot analyses.

### PRPS1 oligomerization assays

PRPS1 oligomerization assays were conducted as previously reported^57^. Then, 20 mM HEPES (pH 7.5) was used to lyse cells, and cells were then centrifuged at 13,000*g* for 10 min at 4 °C. The crosslinking reagent 0.025% glutaraldehyde (Sigma-Aldrich, G5882) was added to the supernatant, and the mixture was incubated at 37 °C for 5 min. The reaction was ended by adding Tris-HCl (pH 8.0, 100 mM). The samples were mixed with loading buffer (Bio-Rad, 1610747) and subjected to immunoblot analyses.

### PRPS1 structure modeling

The cryo-electron microscopy (cryo-EM) structures of human PRPS1 (Protein Data Bank (PDB) ID: 2H06 (ref. ^8^) and PDB ID: 8DBE (ref. ^58^), which contains ADP in both allosteric and catalytic sites) were used to show PRPS1 hexamer, dimer and the residue locations. Pymol (Schrödinger, https://pymol.org/2/) was applied to perform structural analysis and generate the structural figures.

### Enzymatic activity measurement

PRPS1 activity was measured as previously described^11^. Endogenous or recombinant PRPS1 was purified from cells and incubated in 100 µl of freshly prepared reaction buffer containing 50 mM Tris-HCl (pH 7.5), 4.7 mM ribose 5-phosphate (Sigma-Aldrich, 83875), 0.4 mM NADH (Sigma-Aldrich, N8129), 3.2 mM ATP (Sigma-Aldrich, A9187), 1.8 mM phosphoenolpyruvate (Sigma-Aldrich, 860077), 6 mM MgCl_2_ (Thermo Fisher Scientific, 021315), 31 mM NaHCO_3_ (Thermo Fisher Scientific, S5761), 7 U pyruvate kinase (Roche, 56105724), 10 U lactic dehydrogenase (Sigma-Aldrich, L1006) and 10 U myokinase (Sigma-Aldrich, M3003) in a 96-well plate (CELLTREAT Scientific Products, 229196) at 25 °C. Absorbance at 340 nm was read for 15 min by a microplate reader (Molecular Devices, SpectraMax iD3 Multi-Mode Microplate Reader). The changes in the OD value and the intensities of PRPS1 immunoblots were used to quantify the PRPS1 activity.

PRPS activity from human tissues was detected using a PRPP-S Assay Kit (NovoCIB, K0709-04-2) following the protocol from the manufacturer.

ADP and GDP feedback inhibition on PRPS1 activity was detected as previously described^17^ with some modifications. In brief, recombinant PRPS1 was purified from HEK293T cells and incubated in 10 µl of 50 mM Tris-HCl (pH 7.5) buffer containing 2 mM ATP (Sigma-Aldrich, A9187), 2 mM ribose 5-phosphate (Sigma-Aldrich, 83875), 10 mM MgCl_2_ (Thermo Fisher Scientific, 021315) and 1 mM DTT (Roche, 10197777001) and indicated concentrations of ADP (Sigma-Aldrich, 01905) or GDP (Sigma-Aldrich, G7127) for 1 h at 37 °C in a 384-well white board plate (Greiner Bio-One, 784075). ATP was consumed to different degrees during the reaction based on the PRPS1 activity. Reactions were ended with 10 µl of Kinase-Glo reagent (Promega, V6072) being added to each well to terminate the reaction. Luminescence representing the residual ATP amount was detected by a microplate reader (Molecular Devices, SpectraMax iD3 Multi-Mode Microplate Reader). The luminescence signal values were used to calculate the inhibition rate of PRPS1 activity.

### Influx of glucose into RNA and DNA

H1299 cells were cultured with ^14^C-glucose (PerkinElmer, NEC042V) (1 μCi) for about 30 s. Qiagen RNA and DNA kits (74104 and 69504) were used to purify RNA and DNA. The de novo synthesized ^14^C-RNA or ^14^C-DNA was measured using a scintillation counter (Beckman Coulter, LS 6500 Multi-Purpose Scintillation Counter).

### AMPK in vitro kinase assays

The in vitro kinase assays were performed as previously described^34,59^. The PRPS1 *O*-GlcNAcyated peptide (SEWRNCT(O-GlcNAc) IVSPDAGGAKRVTSIADRL) and the corresponding non-*O*-GlcNAcylated peptide were ordered from Chinese Peptide Company. The recombinant *O*-GlcNAcylated and non-*O*-GlcNAcylated GST-PRPS1 proteins were obtained from the in vitro *O*-GlcNAcylation assays as previously described. The purified AMPK (Upstate Biotechnology, 14-840) was incubated with the peptides or the GST-PRPS1 proteins in the kinase reaction buffer (15 mM HEPES (pH 7.0), 450 µM DTT (Roche, 10197777001), 18.75 mM MgCl_2_ (Thermo Fisher Scientific, 021315), 6.25 mM β-glycerophosphate (Sigma-Aldrich, G9422), 1.25 mM EGTA (Sigma-Aldrich, 324626) and 125 µM ATP (Sigma-Aldrich, A9187) with or without 150 µM AMP (Sigma-Aldrich, 01930)) at 30 °C for 15 min. For PRPS1 peptides, S180 phosphorylation was analyzed by MS. For GST-PRPS1 proteins, S180 phosphorylation was analyzed by immunoblotting.

### Metabolite analysis by LC–MS/MS

For cell-based ^13^C_6_ metabolite labeling analysis, around 90% confluent H1299 cells were washed by glucose-free culture medium (Gibco, 11966-025) and cultured in medium containing ^13^C_6_-glucose (10 mM) (Cambridge Isotope Laboratories, CLM-1396) for 30 min. High-resolution LC–MS/MS was used for analyzing the levels of ^13^C_6_-labeled intracellular metabolites^11^.

For label-free xenograft metabolite analysis, the xenograft tissue samples were homogenized in 150 μl of extraction solution (half methanol and half water) with 250 ng ml^−1^ 4-nitrobenzoic acid and debrisoquine as internal standard for negative and positive modes, respectively, followed by adding 150 μl of acetonitrile. After incubation for 30 min at −20 °C, the samples were centrifuged at a speed of 16,000*g* for 30 min at 4 °C. The standards—namely inosine 5′-monophosphate disodium salt (IMP, I4625), adenosine 5′-monophosphate disodium salt (AMP, 01930), guanosine 5′-monophosphate disodium salt (GMP, G8377), uridine 5′-monophosphate disodium salt (UMP, U6375) and cytidine 5′-monophosphate disodium salt (CMP, C1131)—were obtained from Sigma-Aldrich. The samples were transferred to MS vials for LC–MS analysis by QTRAP 7500 System (SCIEX). Data were acquired and normalized to the internal standards and then analyzed with SCIEX OS software.

### MS analysis of PRPS1 O-GlcNAcylation sites

The FLAG-PRPS1 protein isolated from HEK293T cells was digested with sequencing-grade trypsin (Promega, VA9000) at a 50:1 protein-to-trypsin ratio. The incubation process was carried out at 37 °C for a duration of 16 h. Subsequently, the samples were passed through Zip-Tips (Millipore, ZTC18). The LC–MS/MS was conducted using the Easy nLC-1000 system in combination with the Orbitrap Fusion Lumos Tribrid mass spectrometer (Thermo Fisher Scientific)^30,33^.

### Animal experiments

Experiments involving animals adhered to the rules and standards established by the Georgetown University IACUC. The indicated 5 × 10^6^ H1299 cells were introduced into the flank area of male nude mice (Jackson Laboratory, 002019) by subcutaneous injection. Tumor volume was calculated by measuring the width and length of tumors based on the following formula: ${vo}{lume}=[{{length}{\rm{\times }}({width})}^{2}]/2$.

Next, 20 mg kg^−1^ etoposide (Cayman Chemical Company, 12092) was administered to indicated groups intraperitoneally in 200 µl of PBS twice a week for 3 weeks (PBS for controls).

### Statistics and reproducibility

Experiments were repeated independently by indicated biological replicates in the figure legends. Statistical analyses were performed using Prism 8 (GraphPad Software, https://www.graphpad.com). The statistical methods are indicated in corresponding figure legends. Two-tailed Student’s *t*-tests were applied to analyze data from two unpaired groups. Two-tailed Wilcoxon tests were applied to analyze data for two paired groups. The clinical data of human lung cancer subtypes were analyzed by Welch’s *t*-test. *P* values for comparisons are indicated in the figures. The sample size was estimated based on the variations and mean values without employing pre-established statistical techniques.

### Reporting summary

Further information on research design is available in the Nature Portfolio Reporting Summary linked to this article.

## Online content

Any methods, additional references, Nature Portfolio reporting summaries, source data, extended data, supplementary information, acknowledgements, peer review information; details of author contributions and competing interests; and statements of data and code availability are available at 10.1038/s41589-023-01354-x.

### Supplementary information

Supplementary Information Supplementary Tables 1 and 2

Reporting Summary

### Source data

Source Data Fig. 1 Unprocessed western blots

Source Data Fig. 1 Statistical Source Data

Source Data Fig. 2 Unprocessed western blots

Source Data Fig. 3 Unprocessed western blots

Source Data Fig. 3 Statistical Source Data

Source Data Fig. 4 Unprocessed western blots

Source Data Fig. 4 Statistical Source Data

Source Data Fig. 5 Unprocessed western blots

Source Data Fig. 5 Statistical Source Data

Source Data Fig. 6 Unprocessed western blots

Source Data Fig. 6 Statistical Source Data

Source Data Extended Data Fig. 1 Unprocessed western blots

Source Data Extended Data Fig. 1 Statistical Source Data

Source Data Extended Data Fig. 2 Unprocessed western blots

Source Data Extended Data Fig. 3 Unprocessed western blots

Source Data Extended Data Fig. 3 Statistical Source Data

Source Data Extended Data Fig. 4 Unprocessed western blots

Source Data Extended Data Fig. 4 Statistical Source Data

Source Data Extended Data Fig. 5 Unprocessed western blots

Source Data Extended Data Fig. 5 Statistical Source Data

## Data Availability

The source data files related to this study are supplied with this paper. The protein structures of PRPS1 were obtained from the Protein Data Bank (PDB) database (https://www.rcsb.org) with PBD IDs 2H06 and 8DBE. The clinical data of human lung cancer subtypes are accessible from the ULCAN platform (http://ualcan.path.uab.edu/) with *OGT* gene symbol and lung cancer pathological classification. Additional data or details about the paper can be obtained from the lead contact upon reasonable request. Materials produced in this research can also be accessed by reaching out to the lead contact. Source data are provided with this paper.

## Code availability

This study did not generate any original code.
