# Supplementary material for: Direct stimulation of de novo nucleotide synthesis by O-GlcNAcylation
Source: Nat Chem Biol. 2023 Jun 12;20(1):19–29. doi: 10.1038/s41589-023-01354-x (PMC10746546; doi:10.1038/s41589-023-01354-x)
Supplement: Supplementary file 1 — Supplementary Tables 1 and 2 [file 41589_2023_1354_MOESM1_ESM.pdf]

nature chemical biology

Article https://doi.org/10.1038/s41589-023-01354-x

Direct stimulation of de novo nucleotide synthesis by *O*-GlcNAcylation

In the format provided by the authors and unedited

Supplementary Table 1. Human specimen characteristics

Tissue Type	Specimen ID	Gender	Age (Years)
Tumor adjacent normal	hN1	Male	55
	hN2	Male	50
	hN3	Male	58
	hN4	Male	61
	hN5	Male	67
	hN6	Female	65
	hN7	Male	70
	hN8	Male	73
	hN9	Female	80
	hN10	Female	64
	hN11	Male	72
	hN12	Male	64
	hN13	Female	53
	hN14	Male	71
	hN15	Male	73
	hN16	Male	72
	hN17	Male	70
	hN18	Female	64
Tumor	hT1	Male	55
	hT2	Male	50
	hT3	Male	58
	hT4	Male	61
	hT5	Male	67
	hT6	Female	65
	hT7	Male	70
	hT8	Male	73
	hT9	Female	80
	hT10	Female	64
	hT11	Male	72
	hT12	Male	64
	hT13	Female	53
	hT14	Male	71
	hT15	Male	73
	hT16	Male	72
	hT17	Male	70
	hT18	Female	64

Supplementary Table 2. Oligonucleotide sequences

Oligonucleotide	Sequence
sh*OGT*-1	CGACCTGTCTAGACGAGTGAAA
sh*OGT*-2	TTTAGCACTCTGGCAATTAAAC
si-*OGT*-1	GAUUAAGCCUGUUGAAGUC
si-*OGT*-2	GCUUGCAAUUCAUCACUUU
si-*PRPS1*	CGAAAUCAAUGACAAUUUA
sg*PRPS1*	CATGGTGCTTGTGGGAGATG
ACTIN-F	TGGCACCCAGCACAATGAA
ACTIN-R	CTAAGTCATAGTCCGCCTAGAAGCA
OGT-F	TCCTGATTTGTACTGTGTTCGC
OGT-R	AAGCTACTGCAAAGTTCGGTT
PRPS1-F	ATCTTCTCCGGTCCTGCTATT
PRPS1-R	TGGTGACTACTACTGCCTCAAA
PRPS2-F	AGCTCGCATCAGGACCTGT
PRPS2-R	ACGCTTTCACCAATCTCCACG
