# Supplementary material for: Direct stimulation of de novo nucleotide synthesis by O-GlcNAcylation
Source: Nat Chem Biol. 2023 Jun 12;20(1):19–29. doi: 10.1038/s41589-023-01354-x (PMC10746546; doi:10.1038/s41589-023-01354-x)
Supplement: Supplementary file 2 — Reporting Summary [file 41589_2023_1354_MOESM2_ESM.pdf]

nature portfolio

Corresponding author(s): Huadong Pei

Last updated by author(s): Apr 18, 2023

Reporting Summary

Nature Portfolio wishes to improve the reproducibility of the work that we publish. This form provides structure for consistency and transparency in reporting. For further information on Nature Portfolio policies, see our Editorial Policies and the Editorial Policy Checklist.

Statistics

For all statistical analyses, confirm that the following items are present in the figure legend, table legend, main text, or Methods section.

n/a	Confirmed	
☐	☒	The exact sample size (*n*) for each experimental group/condition, given as a discrete number and unit of measurement
☐	☒	A statement on whether measurements were taken from distinct samples or whether the same sample was measured repeatedly
☐	☒	The statistical test(s) used AND whether they are one- or two-sided<br>*Only common tests should be described solely by name; describe more complex techniques in the Methods section.*
☒	☐	A description of all covariates tested
☒	☐	A description of any assumptions or corrections, such as tests of normality and adjustment for multiple comparisons
☐	☒	A full description of the statistical parameters including central tendency (e.g. means) or other basic estimates (e.g. regression coefficient) AND variation (e.g. standard deviation) or associated estimates of uncertainty (e.g. confidence intervals)
☐	☒	For null hypothesis testing, the test statistic (e.g. *F*, *t*, *r*) with confidence intervals, effect sizes, degrees of freedom and *P* value noted<br>*Give P values as exact values whenever suitable.*
☒	☐	For Bayesian analysis, information on the choice of priors and Markov chain Monte Carlo settings
☒	☐	For hierarchical and complex designs, identification of the appropriate level for tests and full reporting of outcomes
☒	☐	Estimates of effect sizes (e.g. Cohen's *d*, Pearson's *r*), indicating how they were calculated

Our web collection on statistics for biologists contains articles on many of the points above.

Software and code

Policy information about availability of computer code

Data collection	Western Blot: GE AI600 imager<br>qPCR:Bio-Rad CFX96 device<br>Immunofluorescence: LEICA SP8 microscope<br>Size-exclusion chromatography:GE AKTA pure<br>Protein structure: Pymol 2.5<br>Plate reader: SpectraMax iD3 Multi-Mode Microplate Readers<br>Scintillation counter: Beckman Coulter, LS 6500 Multi-Purpose Scintillation Counter<br>MS:Thermo Scientific Easy nLC-1000 system coupled with Orbitrap Fusion Lumos Tribrid Mass Spectrometer/QTRAP 7500
Data analysis	Statistical analysis: GraphPad Prism 8.0 version<br>Western blot analysis: Image J 1.53k<br>MS:Sciex OS software 3.0 version

For manuscripts utilizing custom algorithms or software that are central to the research but not yet described in published literature, software must be made available to editors and reviewers. We strongly encourage code deposition in a community repository (e.g. GitHub). See the Nature Portfolio guidelines for submitting code & software for further information.

Data

Policy information about availability of data

All manuscripts must include a data availability statement. This statement should provide the following information, where applicable:

- Accession codes, unique identifiers, or web links for publicly available datasets
- A description of any restrictions on data availability
- For clinical datasets or third party data, please ensure that the statement adheres to our policy

The source data files related to this study are supplied with this paper. The protein structures of PRPS1 were obtained from PDB database (https://www.rcsb.org) with PBD ID: 2H06 and 8DBE. The clinical data of human lung cancer subtypes were accessible from ULCAN platform (http://ualcan.path.uab.edu/) with OGT gene symbol and lung cancer pathological classification. And any further data or details needed about the paper can be obtained from the lead contact upon request. Materials produced in this research can also be accessed by reaching out to the lead contact.

Human research participants

Policy information about studies involving human research participants and Sex and Gender in Research.

Reporting on sex and gender	The lung cancer and adjacent tissues were from 18 lung cancer patients of which there were 13 males and 5 females according to sex. The sex and age data were provided in the extended data table. The data were derived from the medical records of the patients and consent has been obtained for sharing of individual-level data. Sex- and gender-based analyses were not performed considering the small size of the sample.
Population characteristics	The sex and age data were provided in the extended data table.
Recruitment	The involved participants were randomly chosen from the lung cancer patients who underwent surgery in Renmin Hospital of Wuhan University. The random list of patient case numbers was generated with Microsoft Excel software. The selection bias might originate from the patient populations. Because the tissue samples were from patients after surgery which means the patients in advanced stages or having surgical contraindications with no operation chance would be excluded. Therefore, the findings might not largely apply to the inoperable lung cancer patients considering the nature of the selection bias.
Ethics oversight	Ethics Committee of Renmin Hospital of Wuhan University

Note that full information on the approval of the study protocol must also be provided in the manuscript.

Field-specific reporting

Please select the one below that is the best fit for your research. If you are not sure, read the appropriate sections before making your selection.

☒ Life sciences ☐ Behavioural & social sciences ☐ Ecological, evolutionary & environmental sciences

For a reference copy of the document with all sections, see nature.com/documents/nr-reporting-summary-flat.pdf

Life sciences study design

All studies must disclose on these points even when the disclosure is negative.

Sample size	No sample size calculation was performed. The sample size was determined considering the variations and mean values of the samples, or based on previous observations or a standard protocol in the field. Sample size for each experiment is indicated in the figures or corresponding figure legends, or in the "Statistics and reproducibility" part in the manuscript.
Data exclusions	No data was excluded from analyses.
Replication	All biological replicates are obtained from biologically independent experiments. All attempts at replication were successful. The experiment numbers are indicated in the figure legends or "Statistics and reproducibility" part.
Randomization	Samples/mice/participants were randomized allocated into control and experimental groups.
Blinding	The investigators were blinded to group allocation during data collection.

Reporting for specific materials, systems and methods

We require information from authors about some types of materials, experimental systems and methods used in many studies. Here, indicate whether each material, system or method listed is relevant to your study. If you are not sure if a list item applies to your research, read the appropriate section before selecting a response.

Materials & experimental systems

n/a	Involved in the study
☐	☒ Antibodies
☐	☒ Eukaryotic cell lines
☒	☐ Palaeontology and archaeology
☐	☒ Animals and other organisms
☒	☐ Clinical data
☒	☐ Dual use research of concern

Methods

n/a	Involved in the study
☒	☐ ChIP-seq
☒	☐ Flow cytometry
☒	☐ MRI-based neuroimaging

Antibodies

Antibodies used

Anti-PRPS1 (Proteintech, Cat#15549-1-AP; RRID: AB_10694269; 1:1000);
Anti-Phospho-PRPS1 (Ser180) (Thermo Fisher Scientific, Cat#PA5-106230; RRID: AB_2817628; 1:600);
Anti-PRPS2 (Novus, Cat#NBP1-31435; RRID: AB_2300290; 1:1000);
Anti-OGT (Cell Signaling Technology, Cat#24083; RRID: AB_2716710; 1:1000);
Anti-OGA (Proteintech, Cat#14711-1-AP; RRID: AB_2143063;1:1000);
Anti-AMPKα (Cell Signaling Technology, Cat#2532; RRID: AB_330331;1:1000);
Anti-Phospho-AMPKα (Thr172) (Cell Signaling Technology, Cat#2535; RRID: AB_331250; 1:1000);
Anti-ACC (Cell Signaling Technology, Cat#3662; RRID: AB_2219400; 1:1000);
Anti-Phospho-ACC (Ser79) (Cell Signaling Technology, Cat#3661; RRID: AB_330337; 1:1000);
Anti-β-Tubulin (Proteintech, Cat#66240-1-Ig; RRID: AB_2881629; 1:2500);
Anti-β-Actin (Proteintech, Cat#60008-1-Ig; RRID: AB_2289225; 1:2500);
Anti-γ-H2AX (Ser139) (Cell Signaling Technology, Cat#9718; RRID: AB_2118009; 1:1000);
Anti-Histone H3 (Millipore, Cat#06-755; RRID: AB_11211742; 1:1000);
Anti-O-GlcNAc (RL2) (Abcam, Cat#ab2739; RRID:AB_303264; 1:1000);
Anti-O-GlcNAc (CTD110.6) (Cell Signaling Technology, Cat#9875; RRID: AB_10950973; 1:1000);
Anti-O-GlcNAc (18B10.C7) (Thermo Fisher Scientific, Cat#MA1-038; RRID: AB_2536725; 1:1000);
Anti-FLAG (Sigma-Aldrich, Cat#F3165; RRID: AB_259529; 1:4000);
Anti-HA (BioLegend, Cat#MMS-101P, Clone 16B12; RRID:AB_2314672; 1:2000);
Anti-HA (Cell Signaling Technology, Cat#3724; RRID: AB_1549585; 1:2000);
Anti-c-Myc (Santa Cruz Biotechnology, Cat#sc-40; RRID: AB_627268; 1:2000).
The secondary antibodies (Jackson ImmunoResearch, Cat#115-035-003; RRID: AB_10015289,1:5000, and Cat# 111-035-003; RRID: AB_2313567, 1:5000; Cat# 111-585-045; RRID: AB_2338062,1:200)

Validation

All the antibodies listed above were validated in western blotting and Anti-γ-H2AX was validated in immunofluorescence staining in this manuscript. All the antibodies listed above were validated in human cells. Anti-FLAG, Anti-O-GlcNAc (RL2), Anti-O-GlcNAc (CTD110.6), Anti-ACC, Anti-Phospho-ACC (Ser79), Anti-AMPKα, Anti-Phospho-AMPKα (Thr172), and Anti-β-Tubulin were validated in mouse MEFs.
All commercial antibodies have been validated by the manufacturers (see website links below):
Anti-PRPS1 (Proteintech, Cat#15549-1-AP; RRID: AB_10694269): https://www.ptglab.com/products/PRPS1-Antibody-15549-1-AP.htm
Anti-Phospho-PRPS1 (Ser180) (Thermo Fisher Scientific, Cat#PA5-106230; RRID: AB_2817628): https://www.thermofisher.com/antibody/product/Phospho-PRPS1-Ser180-Antibody-Polyclonal/PA5-106230
Anti-PRPS2 (Novus, Cat#NBP1-31435; RRID: AB_2300290): https://www.novusbio.com/products/prps2-antibody_nbp1-31435
Anti-OGT (Cell Signaling Technology, Cat#24083; RRID: AB_2716710): https://www.cellsignal.com/products/primary-antibodies/ogt-d1d8q-rabbit-mab/24083
Anti-OGA (Proteintech, Cat#14711-1-AP; RRID: AB_2143063): https://www.ptglab.com/products/MGEA5-Antibody-14711-1-AP.htm
Anti-AMPKα (Cell Signaling Technology, Cat#2532; RRID: AB_330331): https://www.cellsignal.com/products/primary-antibodies/ampka-antibody/2532
Anti-Phospho-AMPKα (Thr172) (Cell Signaling Technology, Cat#2535; RRID: AB_331250): https://www.cellsignal.com/products/primary-antibodies/phospho-ampka-thr172-40h9-rabbit-mab/2535
Anti-ACC (Cell Signaling Technology, Cat#3662; RRID: AB_2219400): https://www.cellsignal.com/products/primary-antibodies/acetyl-coa-carboxylase-antibody/3662
Anti-Phospho-ACC (Ser79) (Cell Signaling Technology, Cat#3661; RRID: AB_330337): https://www.cellsignal.com/products/primary-antibodies/phospho-acetyl-coa-carboxylase-ser79-antibody/3661
Anti-β-Tubulin (Proteintech, Cat#66240-1-Ig; RRID: AB_2881629): https://www.ptglab.com/products/Tubulin-beta-Antibody-66240-1-Ig.htm
Anti-β-Actin (Proteintech, Cat#60008-1-Ig; RRID: AB_2289225): https://www.ptglab.com/products/ACTB-Antibody-60008-1-Ig.htm
Anti-γ-H2AX (Ser139) (Cell Signaling Technology, Cat#9718; RRID: AB_2118009): https://www.cellsignal.com/products/primary-antibodies/phospho-histone-h2a-x-ser139-20e3-rabbit-mab/9718
Anti-Histone H3 (Millipore, Cat#06-755; RRID: AB_11211742): https://www.emdmillipore.com/US/en/product/Anti-Histone-H3-Antibody,MM_NF-06-755
Anti-O-GlcNAc (RL2) (Abcam, Cat#ab2739; RRID:AB_303264): https://www.abcam.com/o-linked-n-acetylglucosamine-antibody-rl2-ab2739.html
Anti-O-GlcNAc (CTD110.6) (Cell Signaling Technology, Cat#9875; RRID: AB_10950973): https://www.cellsignal.com/products/primary-antibodies/o-glcnac-ctd110-6-mouse-mab/9875
Anti-O-GlcNAc (18B10.C7) (Thermo Fisher Scientific, Cat#MA1-038; RRID: AB_2536725): https://www.thermofisher.com/antibody/product/O-linked-N-acetylglucosamine-O-GlcNAc-Antibody-clone-18B10-C7-Monoclonal/MA1-038

Anti-FLAG (Sigma-Aldrich, Cat#F3165; RRID: AB_259529): https://www.sigmaaldrich.com/US/en/product/sigma/f3165
Anti-HA (BioLegend, Cat#MMS-101P, Clone 16B12; RRID:AB_2314672): https://www.biolegend.com/en-gb/search-results/purified-anti-ha-11-epitope-tag-antibody-11374?GroupID=GROUP26
The secondary antibodies (Jackson ImmunoResearch, Cat#115-035-003; RRID: AB_10015289, and Cat# 111-035-003; RRID: AB_2313567; Cat# 111-585-045; RRID: AB_2338062) :https://www.jacksonimmuno.com/?utm_term=jackson%20immunoresearch&utm_campaign=Products+%5BSearch,+USA%5D&utm_source=adwords&utm_medium=ppc&hsa_acc=9495885278&hsa_cam=1338042942&hsa_grp=54692580946&hsa_ad=589091855883&hsa_src=g&hsa_tgt=kwd-324200759657&hsa_kw=jackson%20immunoresearch&hsa_mt=e&hsa_net=adwords&hsa_ver=3&gclid=Cj0KCQjwlumhBhClARIsABO6p-xFxGtepQMIk35zexQfG31M0gNIVXmYXKRufBRNnozhG7eCVxWc59caApMhEALw_wcB
Anti-HA (Cell Signaling Technology, Cat#3724; RRID: AB_1549585): https://www.cellsignal.com/products/primary-antibodies/ha-tag-c29f4-rabbit-mab/3724
Anti-c-Myc (Santa Cruz Biotechnology, Cat#sc-40; RRID: AB_627268): https://www.scbt.com/p/c-myc-antibody-9e10

Eukaryotic cell lines

Policy information about cell lines and Sex and Gender in Research

Cell line source(s)	H1299 (ATCC, Cat#CRL-5803; RRID: CVCL_0060) (isolated from the lung of a 43-year-old, male patient with carcinoma), A549 (ATCC, Cat#CCL-185; RRID: CVCL_0023) (isolated from the lung tissue of a 58-year-old male with lung cancer), and HEK293T (ATCC, Cat#CRL-11268; RRID: CVCL_1926) (isolated from human embryo kidney tissue) cell lines were purchased from ATCC. Ampk+/+ and Ampk−/− mouse embryonic fibroblasts (MEFs) cells were kindly provided by Dr. Eduardo Chini (Mayo Clinic, MN).
Authentication	All cell lines were not authenticated beyond inspection based on morphological criteria.
Mycoplasma contamination	The cell lines are routinely tested using mycoplasma detection kit. All cell lines were tested negative for mycoplasma.
Commonly misidentified lines (See ICLAC register)	No commonly misidentified cell lines were used.

Animals and other research organisms

Policy information about studies involving animals; ARRIVE guidelines recommended for reporting animal research, and Sex and Gender in Research

Laboratory animals	Nude mice (6-8 weeks) Strain #:002019;RRID:IMSR_JAX:002019
Wild animals	This study did not involve wild animals.
Reporting on sex	Male nude mice were employed. The numbers of mice were indicated in figures or corresponding figure legends. The reason for choosing male mice is because OGT is an X-linked gene product and Ogt deletion in mice led to severe postnatal lethality. Male nude mice alive share the same status of Ogt and are better models to investigate the role of O-GlcNAcylation between different groups in vivo. In addition, if female mice are at different points in their estrous cycle, their statuses might vary. Mouse sex-based analyses were not performed.
Field-collected samples	This study did not involve field-collected samples.
Ethics oversight	Animal procedures were performed according to a protocol approved (2021-0065) by the Institutional Animal Care and Use Committee (IACUC) at Georgetown University.

Note that full information on the approval of the study protocol must also be provided in the manuscript.
