# Supplementary figures and images for: Direct stimulation of de novo nucleotide synthesis by O-GlcNAcylation
Source: Nat Chem Biol. 2023 Jun 12;20(1):19–29. doi: 10.1038/s41589-023-01354-x (PMC10746546; doi:10.1038/s41589-023-01354-x)

**Fig. 1b**

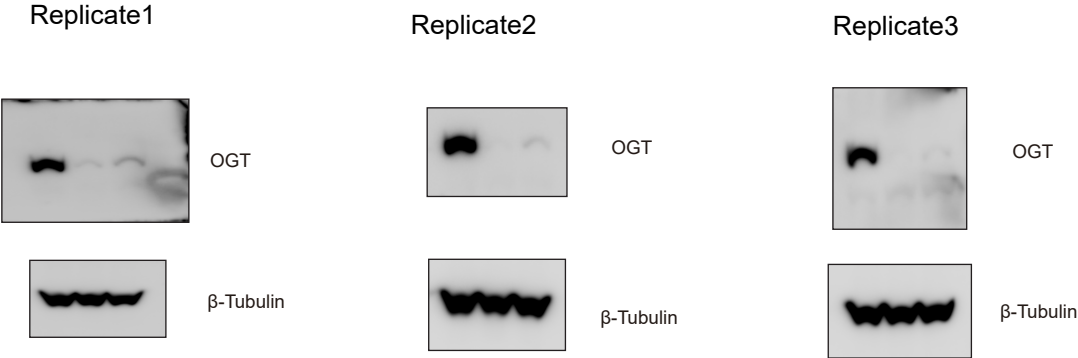

**Fig. 1i**

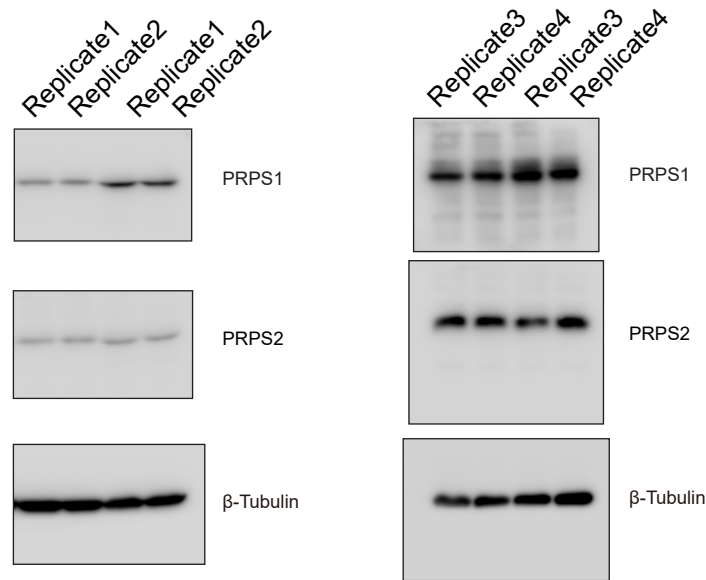

**Fig. 1j**

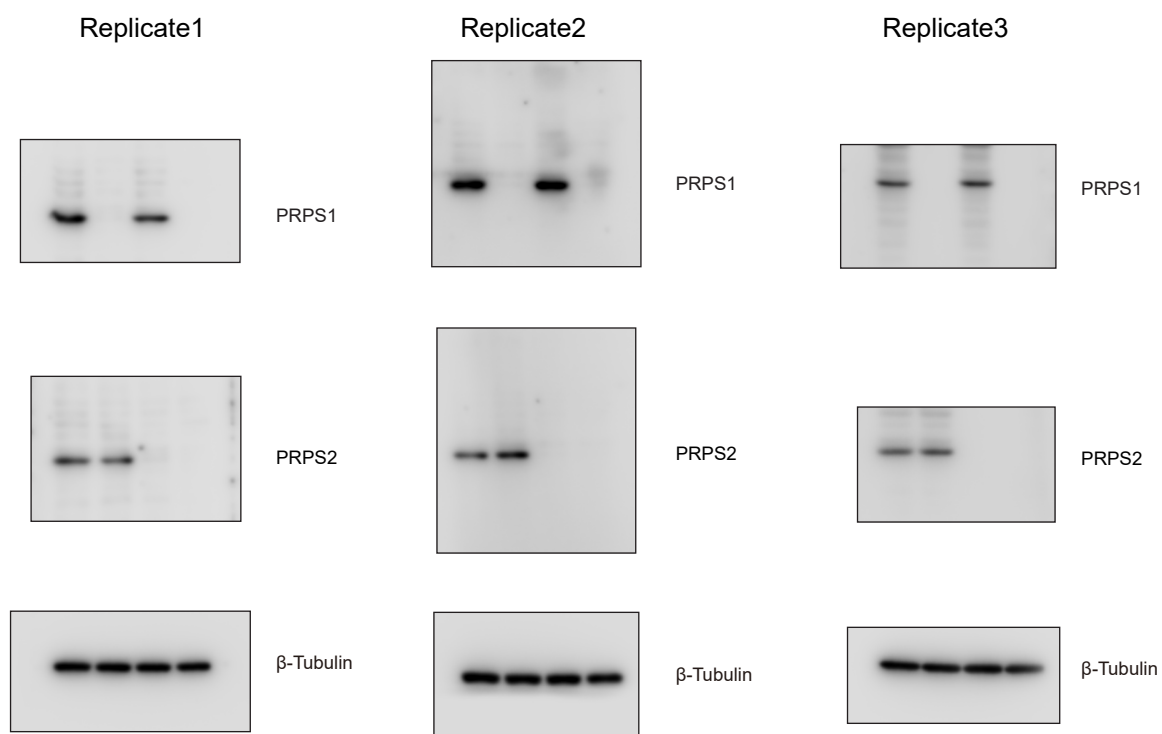

**Fig. 1k**

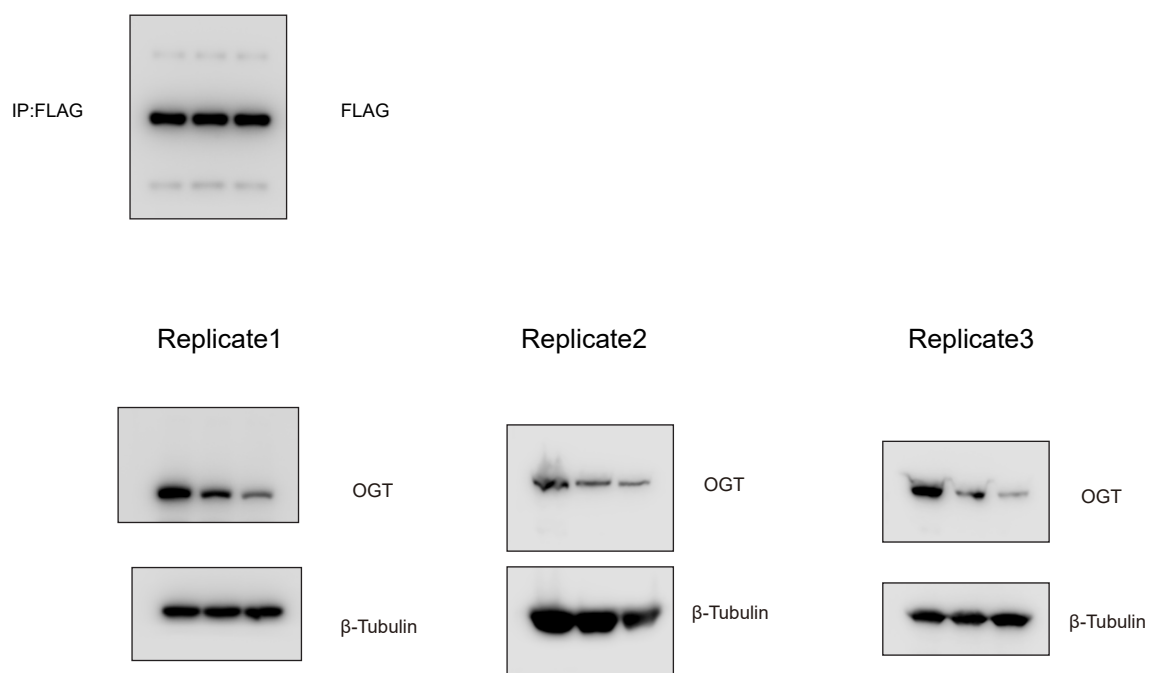

Supplement: Supplementary file 3 — Unprocessed western blots [file 41589_2023_1354_MOESM3_ESM.pdf]

Fig. 2a

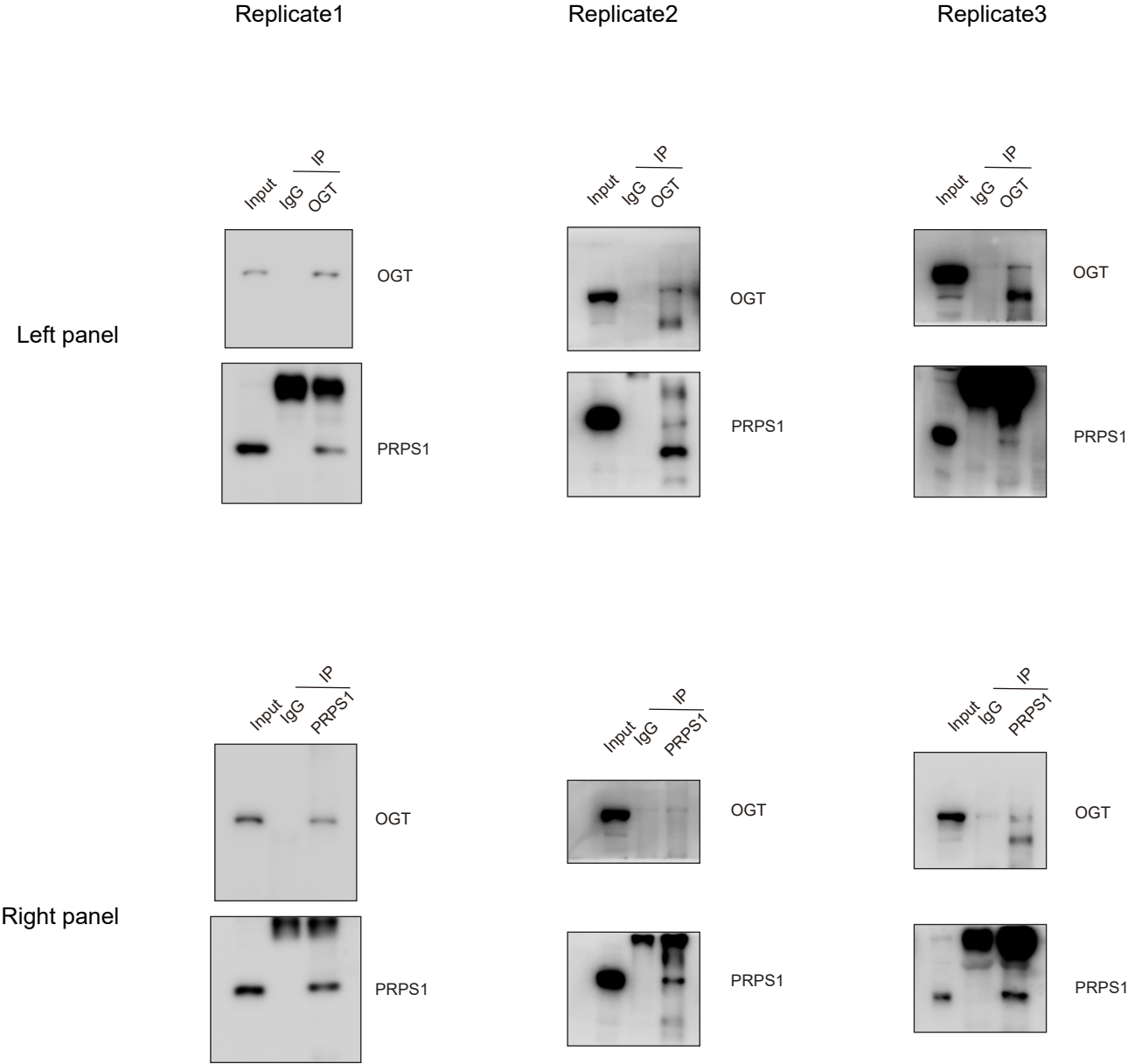

Fig. 2b

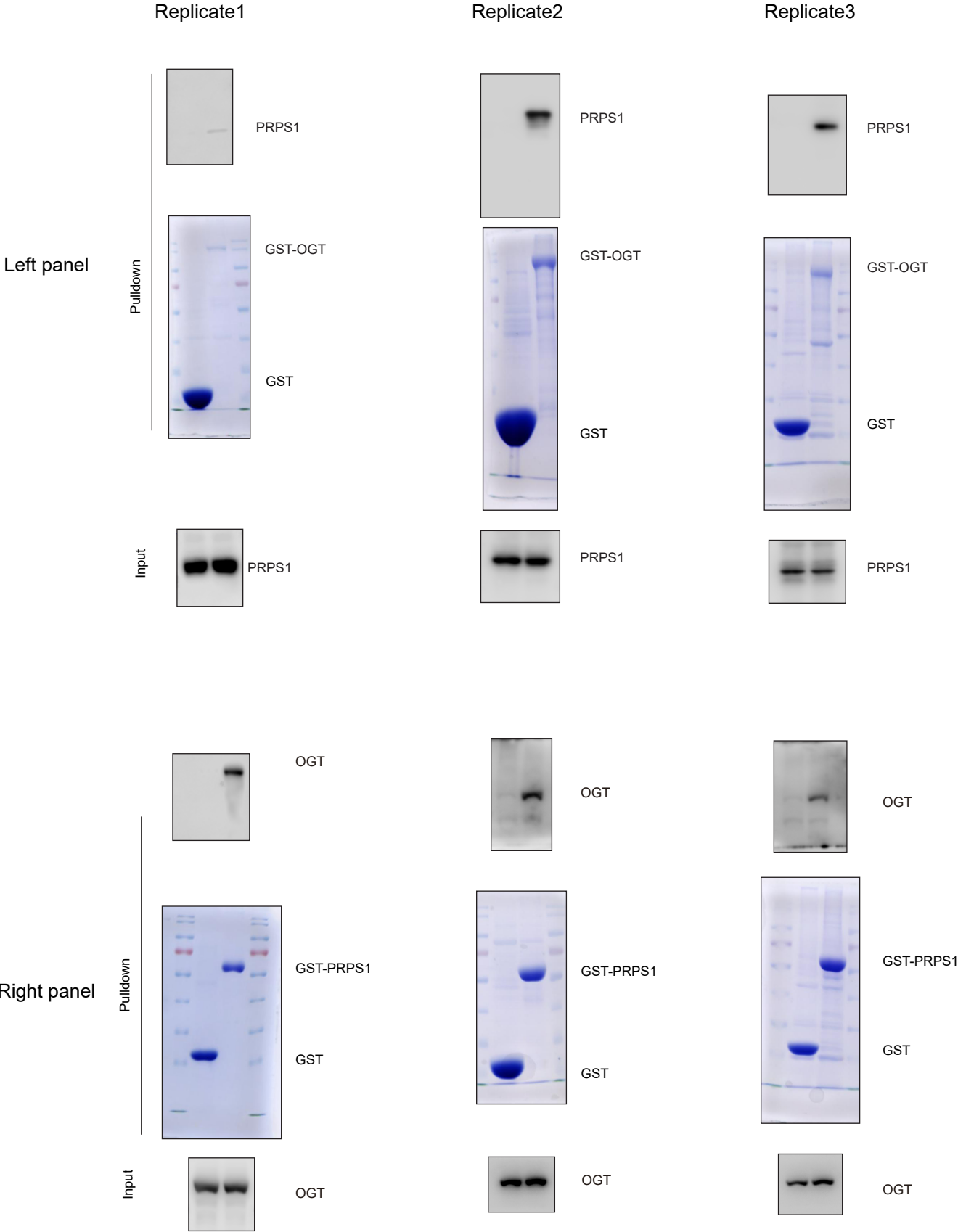

Fig. 2c

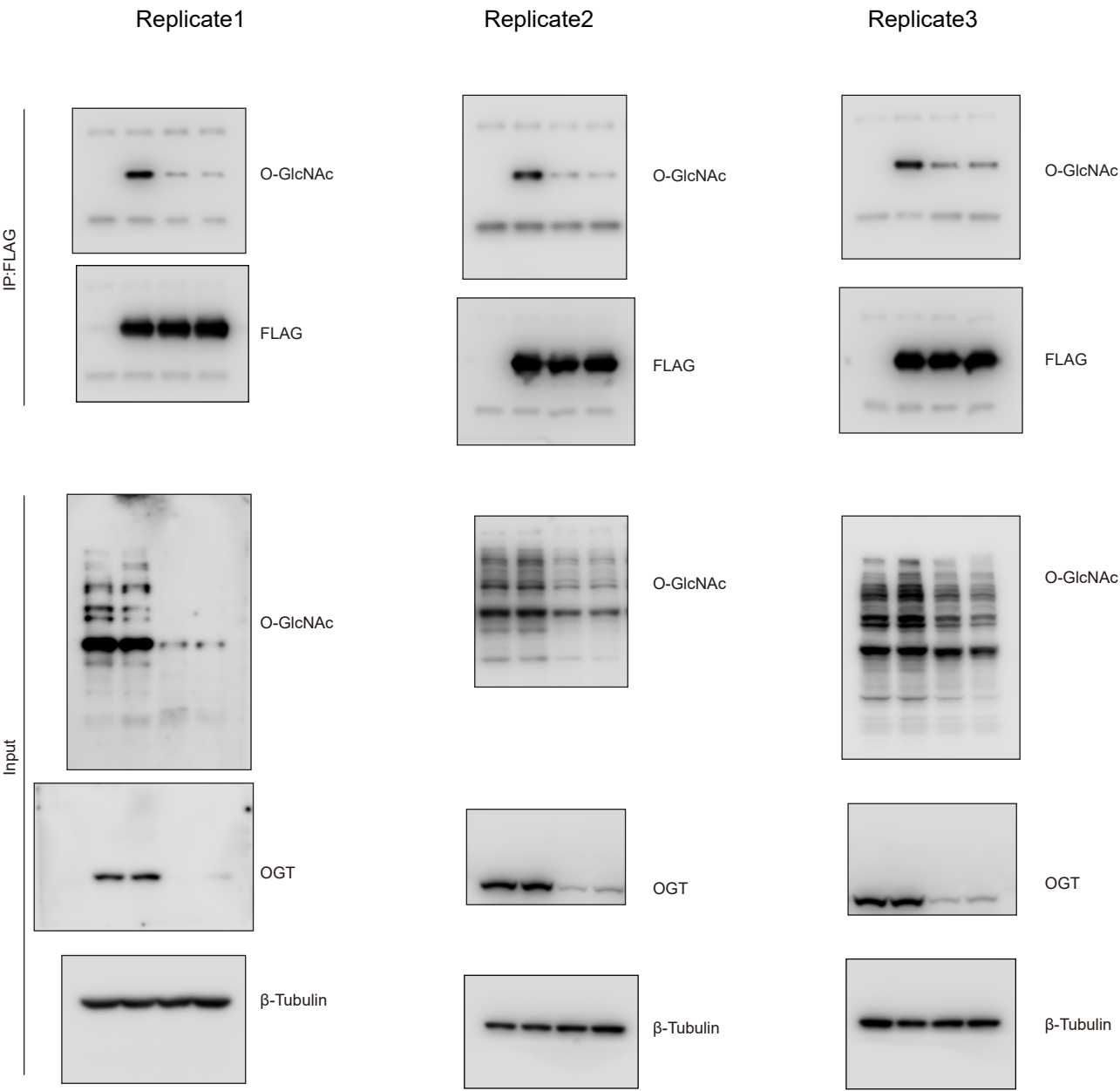

Fig. 2d

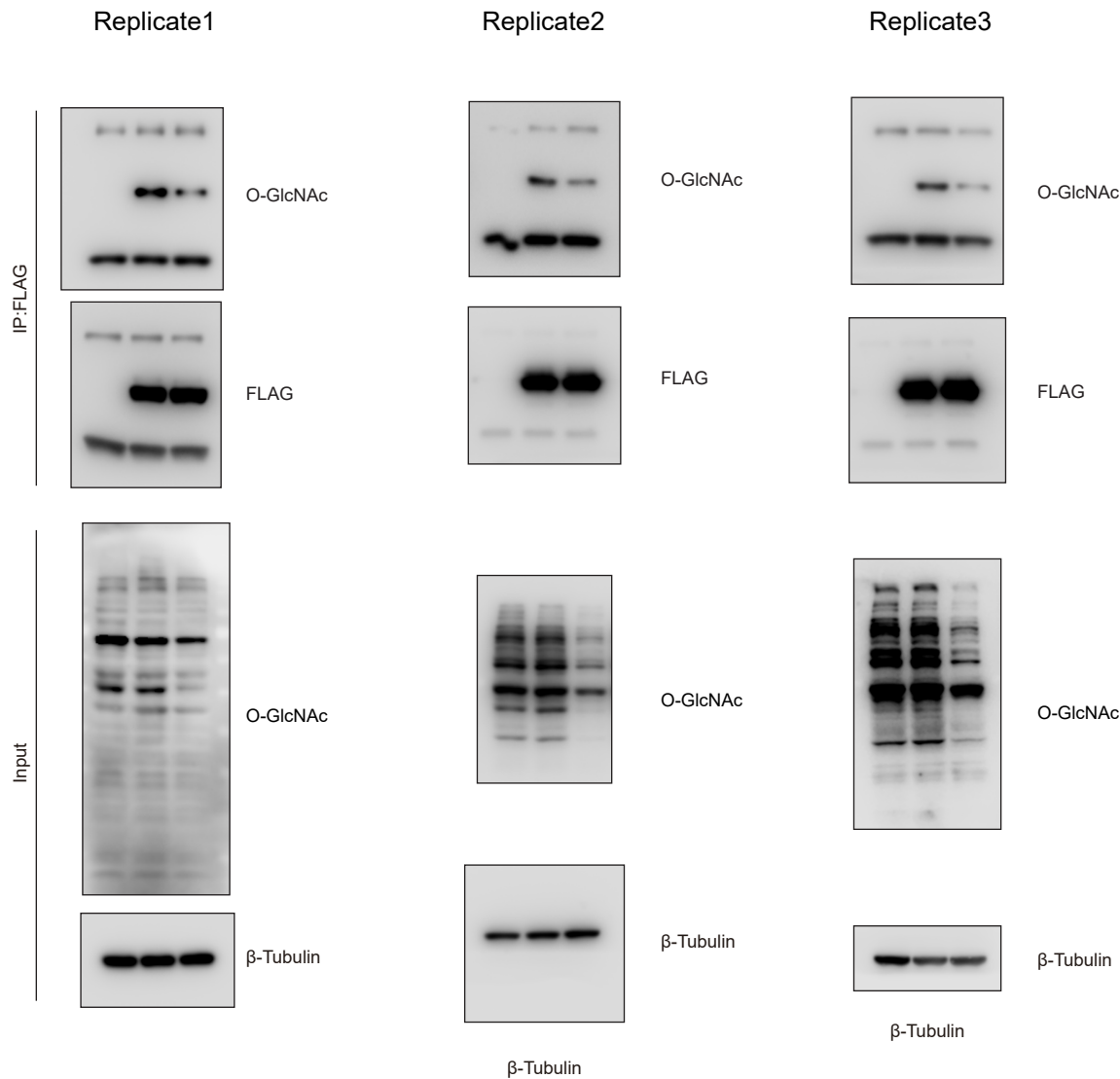

**Fig. 2e**

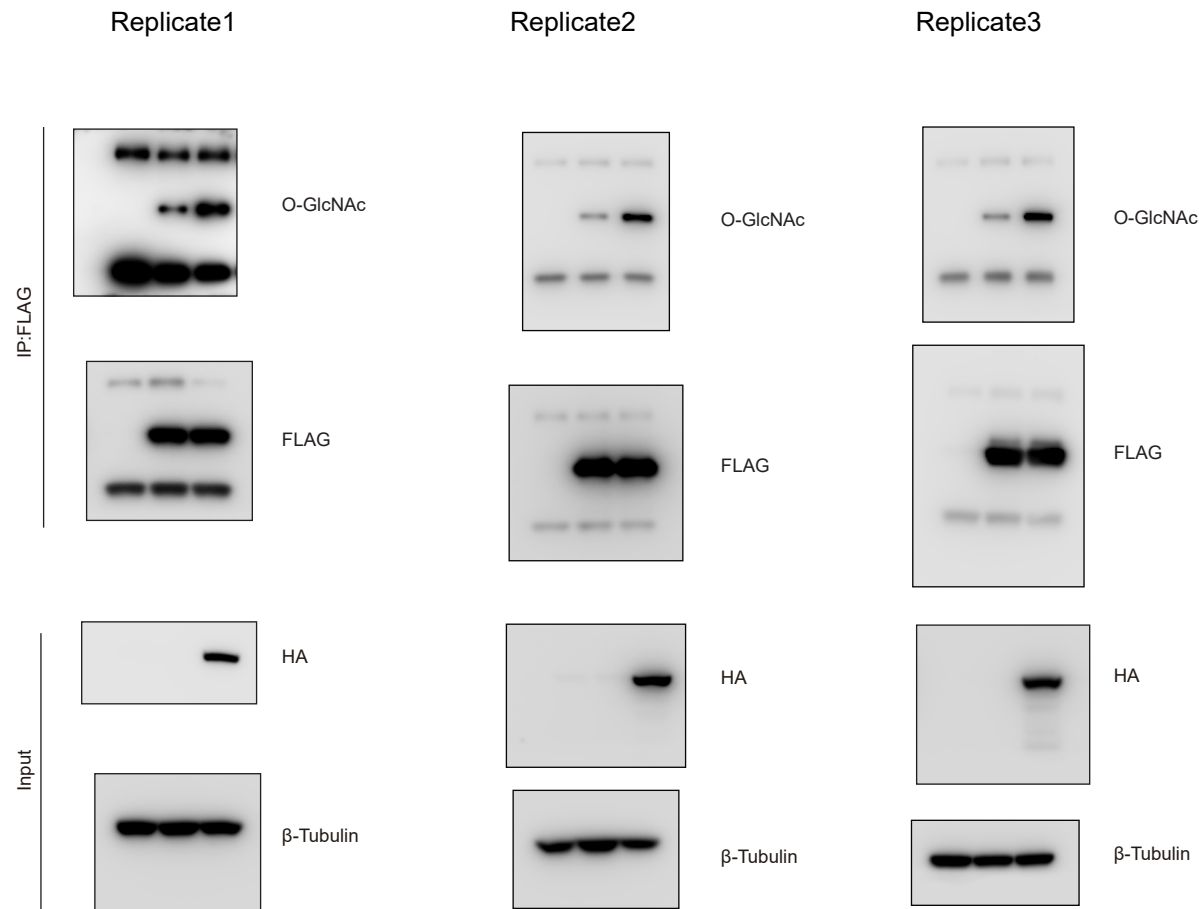

**Fig. 2f**

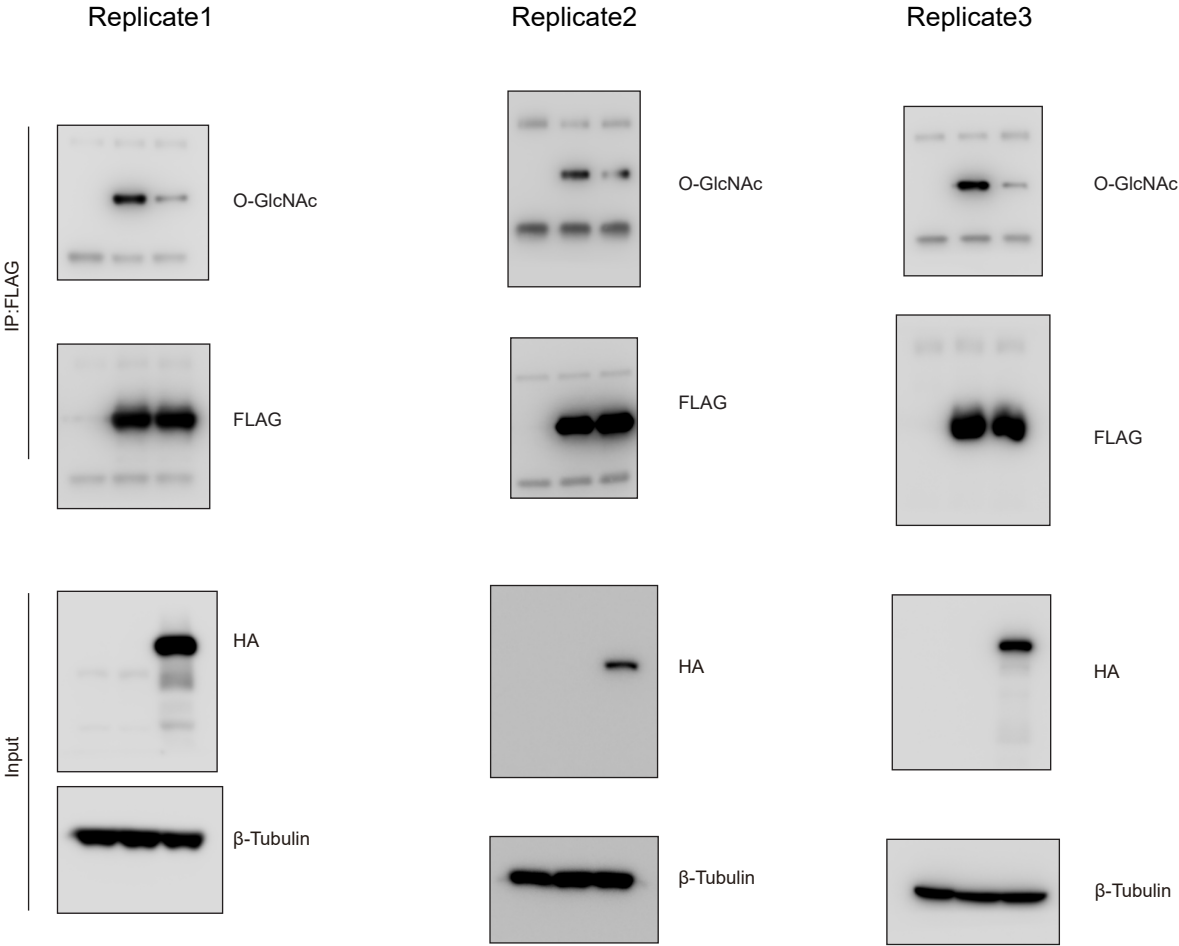

**Fig. 2g**

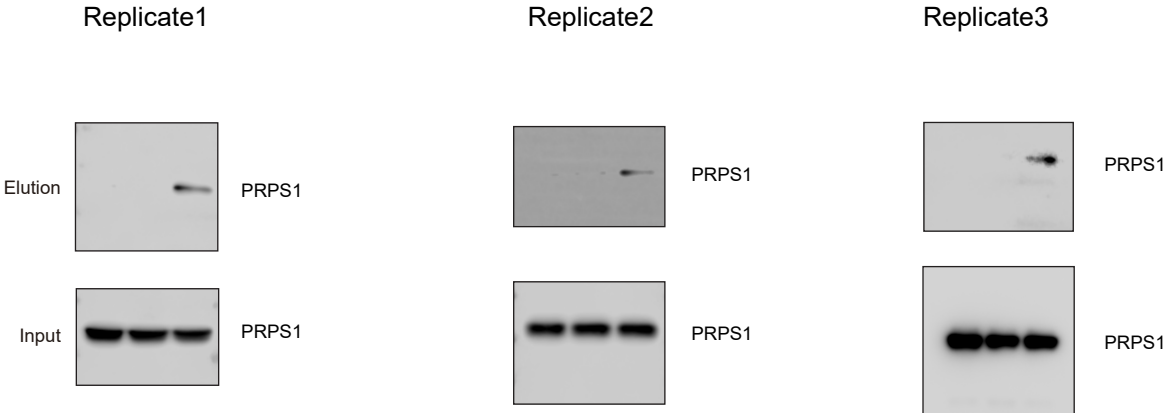

Fig. 2h

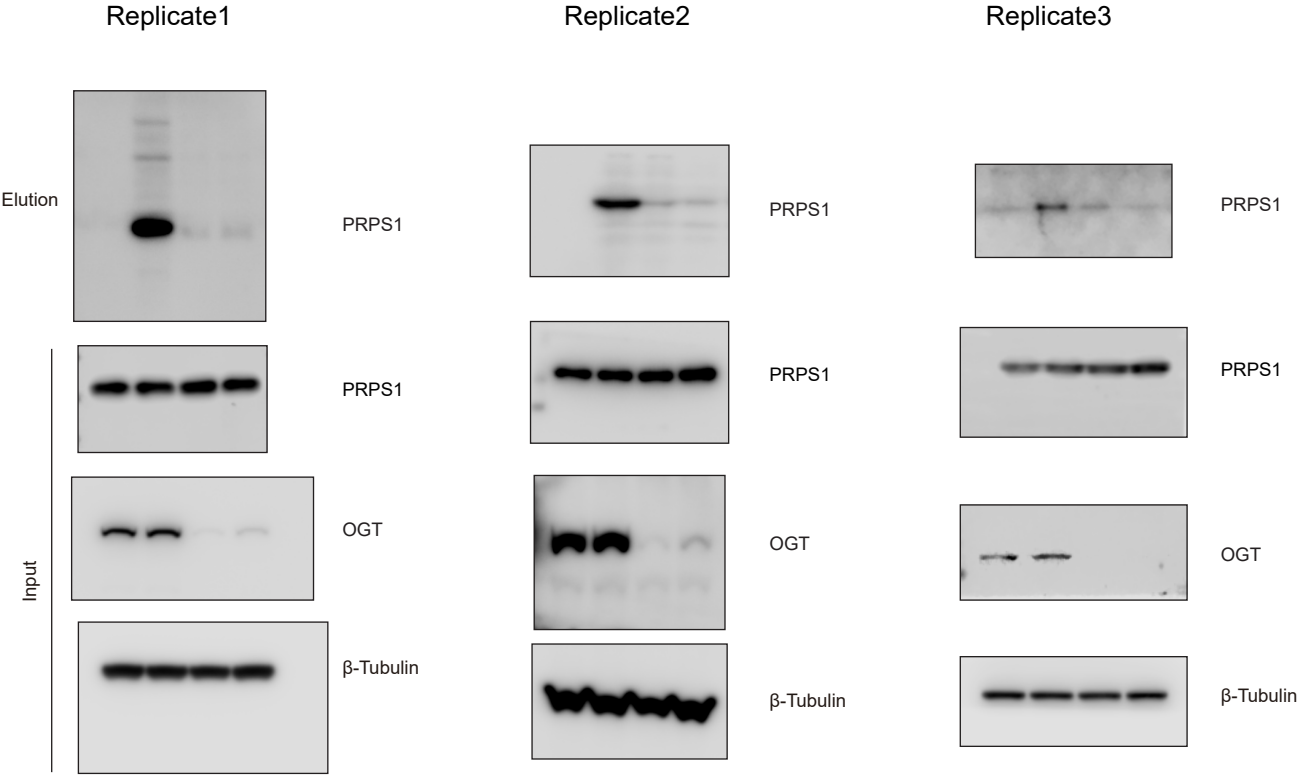

**Fig. 2i**

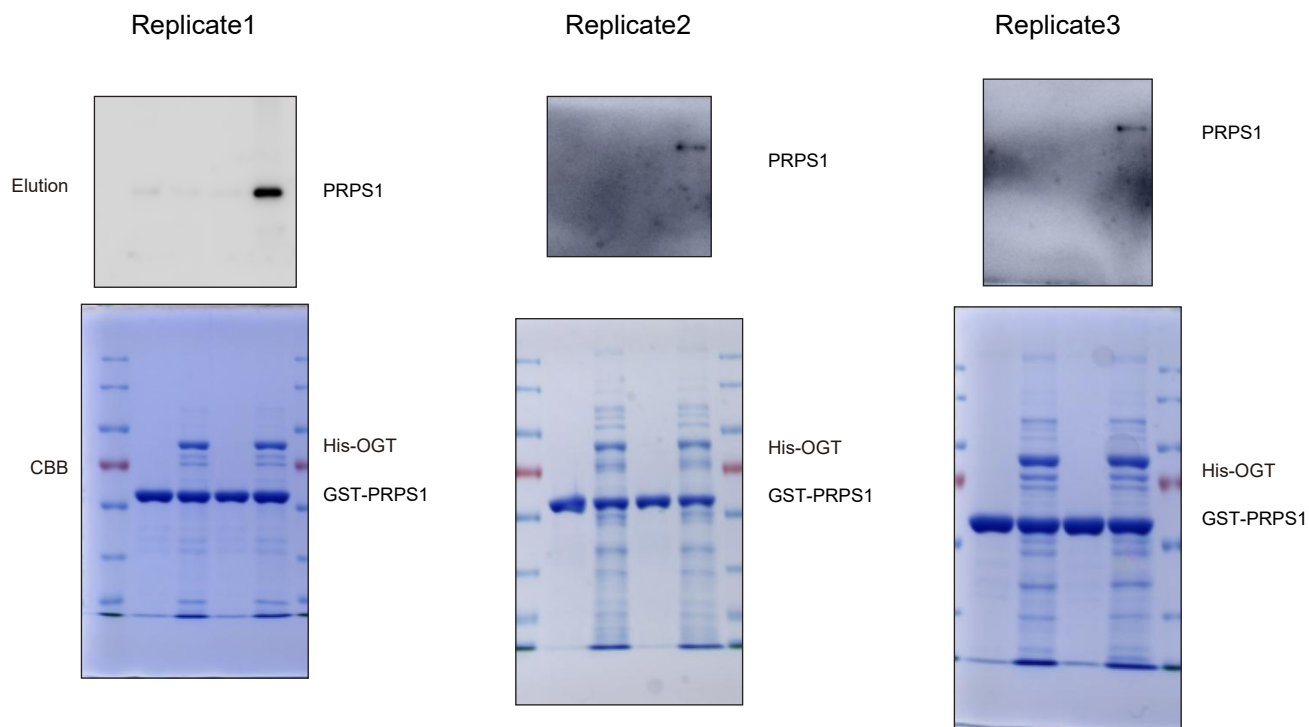

**Fig. 2j**

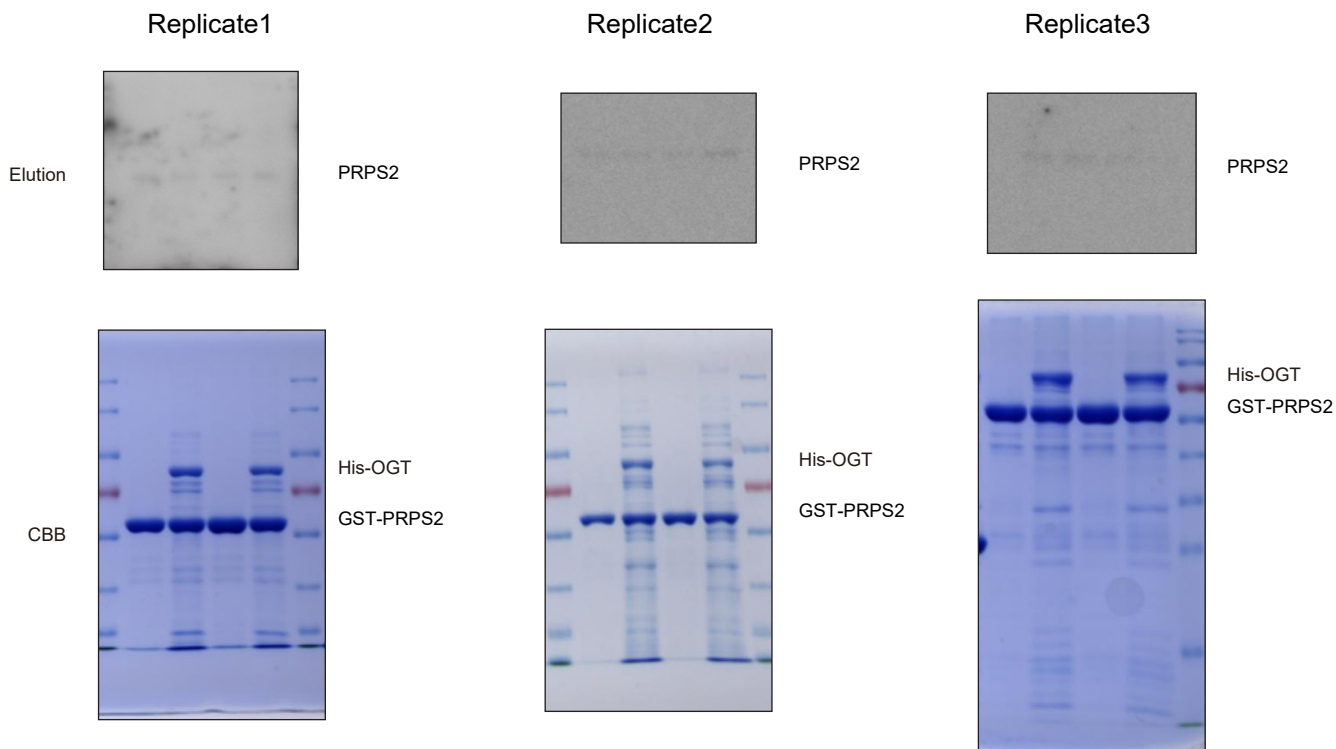

Fig. 2k

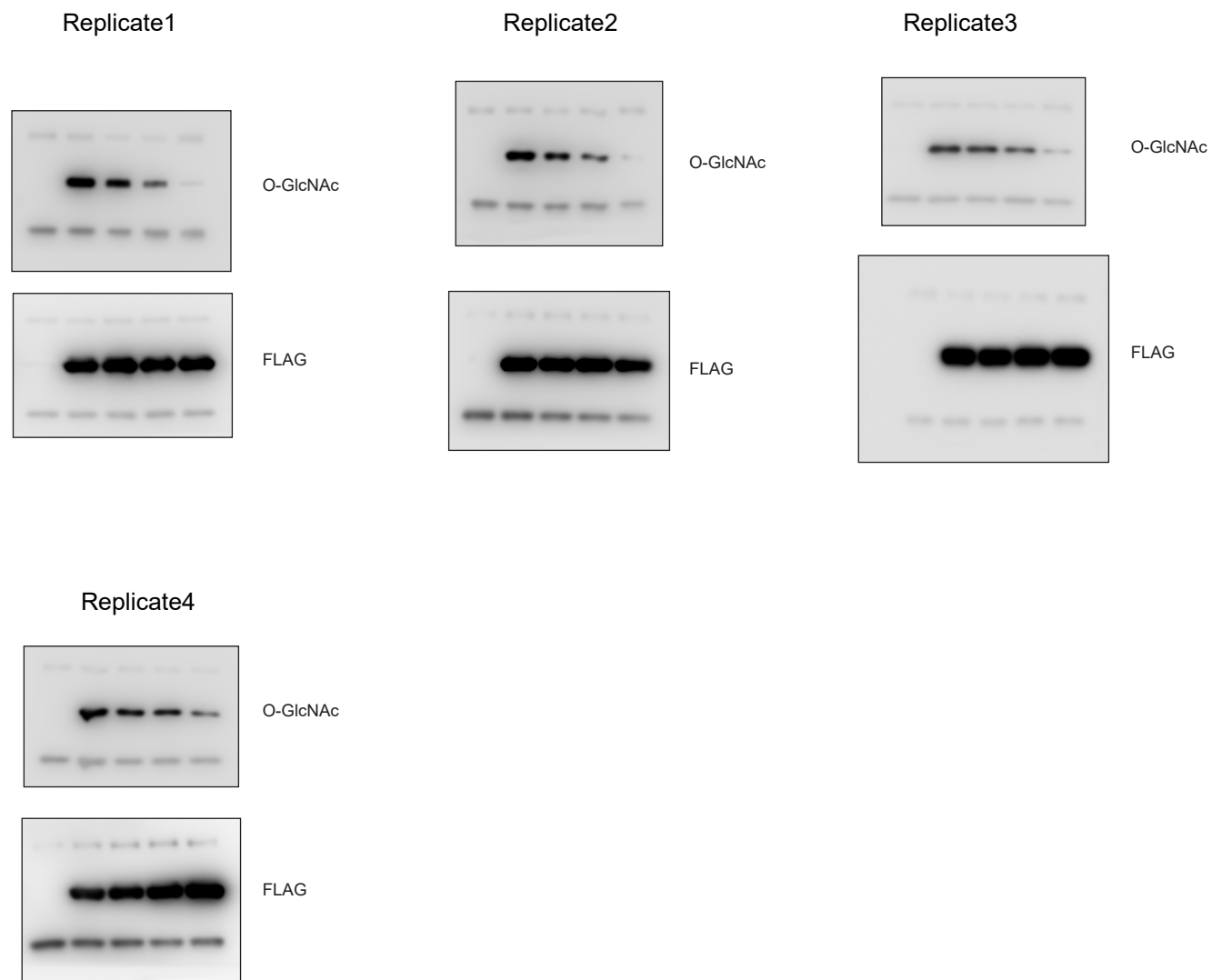

Fig. 2I

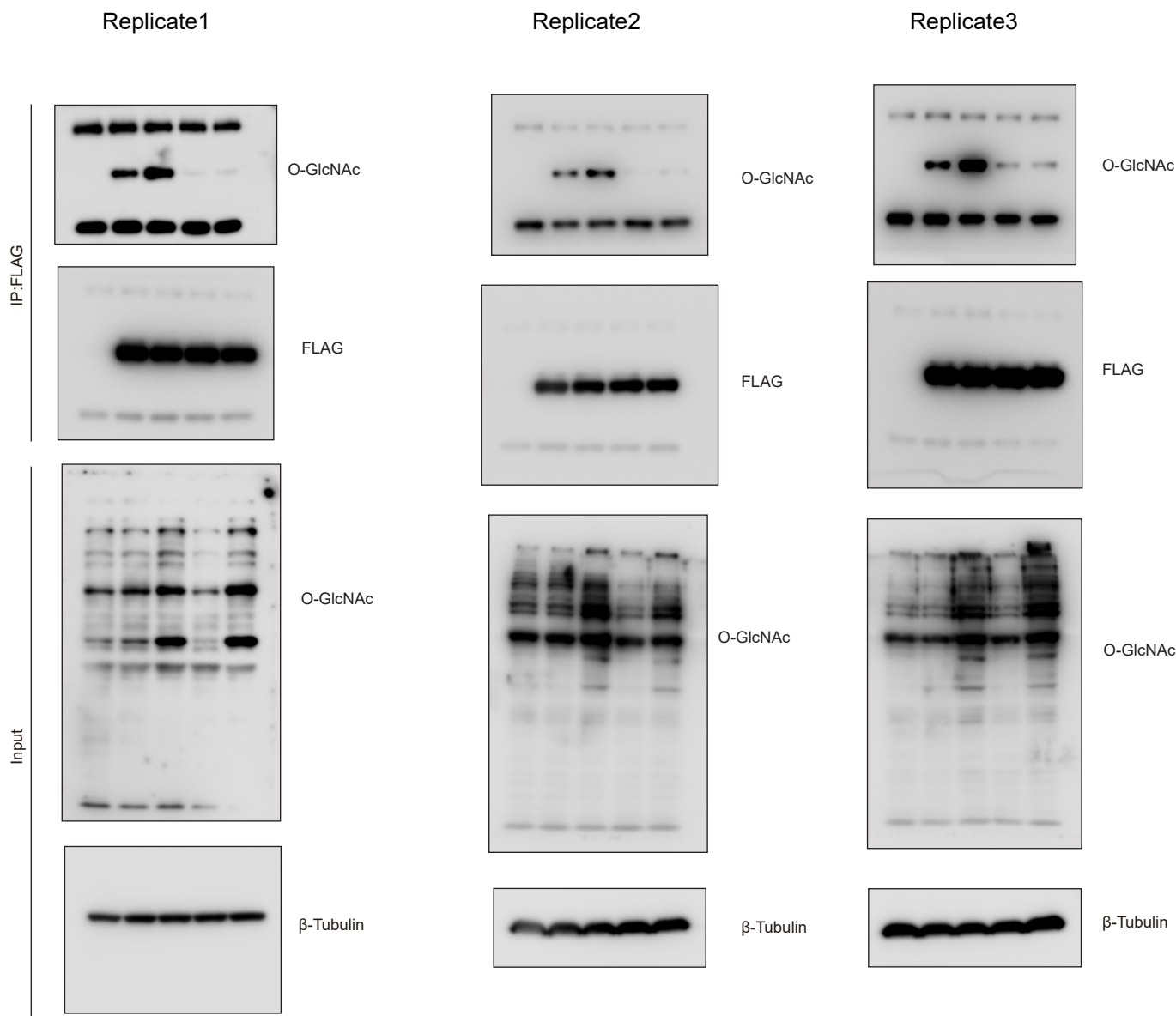

Supplement: Supplementary file 5 — Unprocessed western blots [file 41589_2023_1354_MOESM5_ESM.pdf]

Fig. 3a

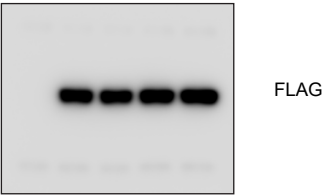

Fig. 3d

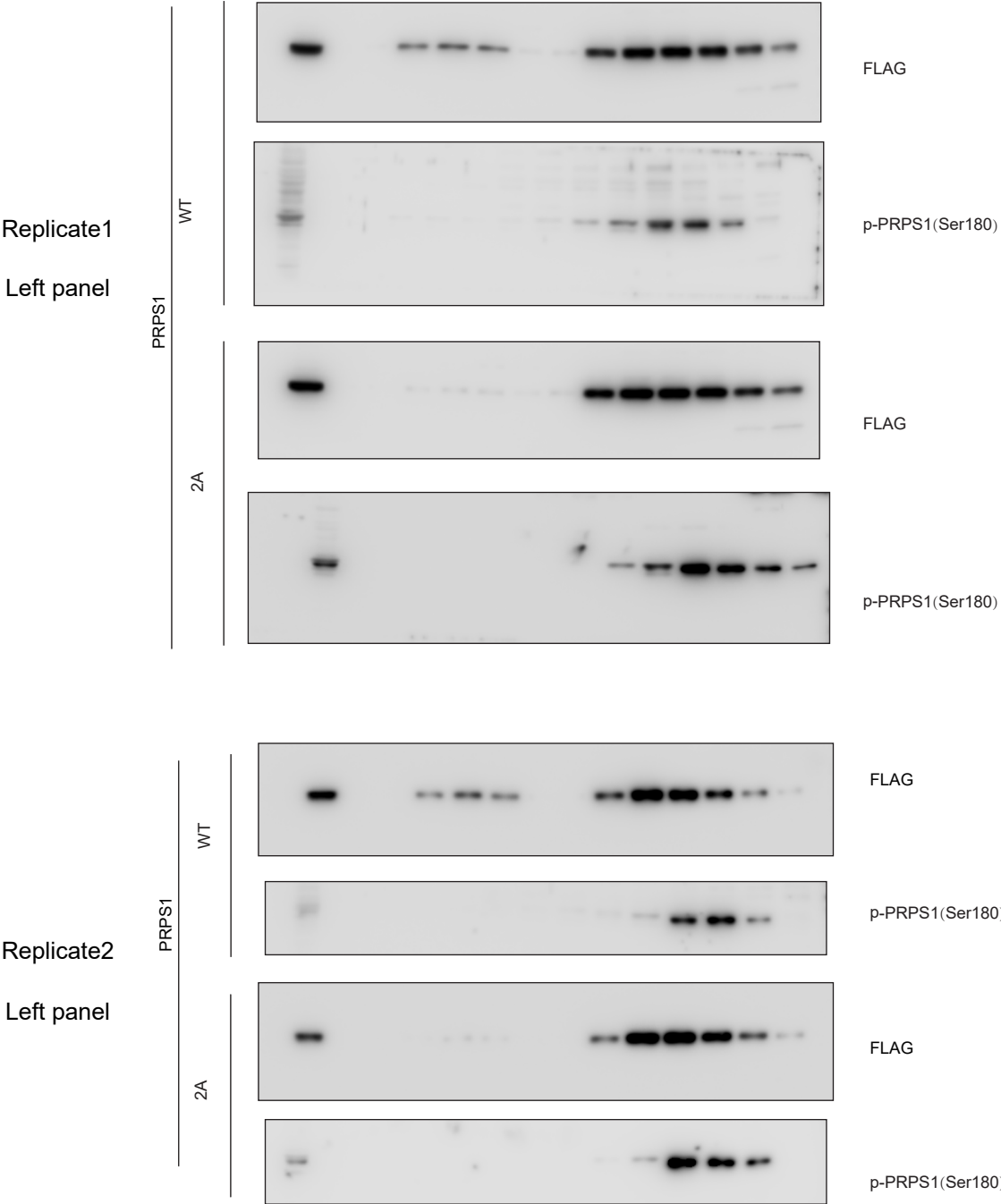

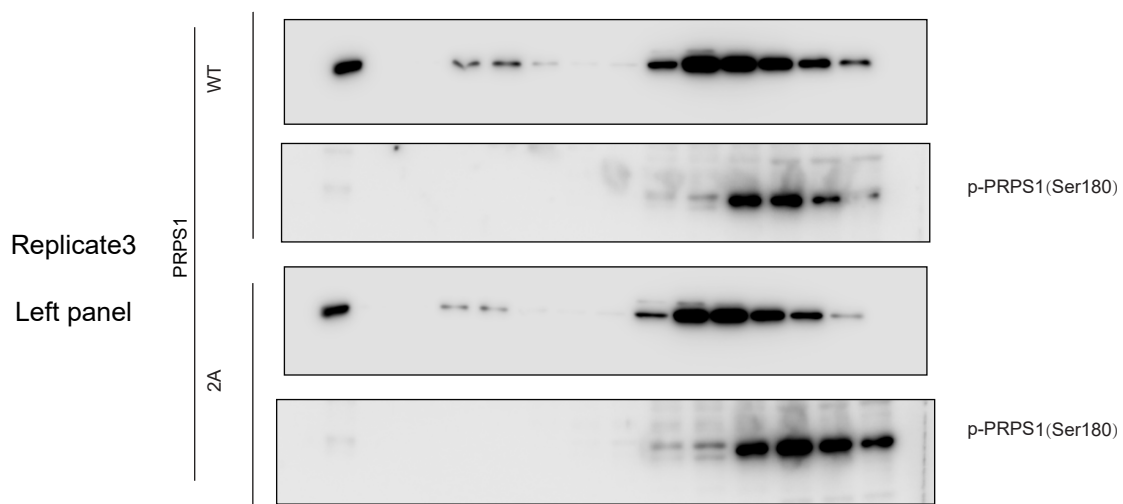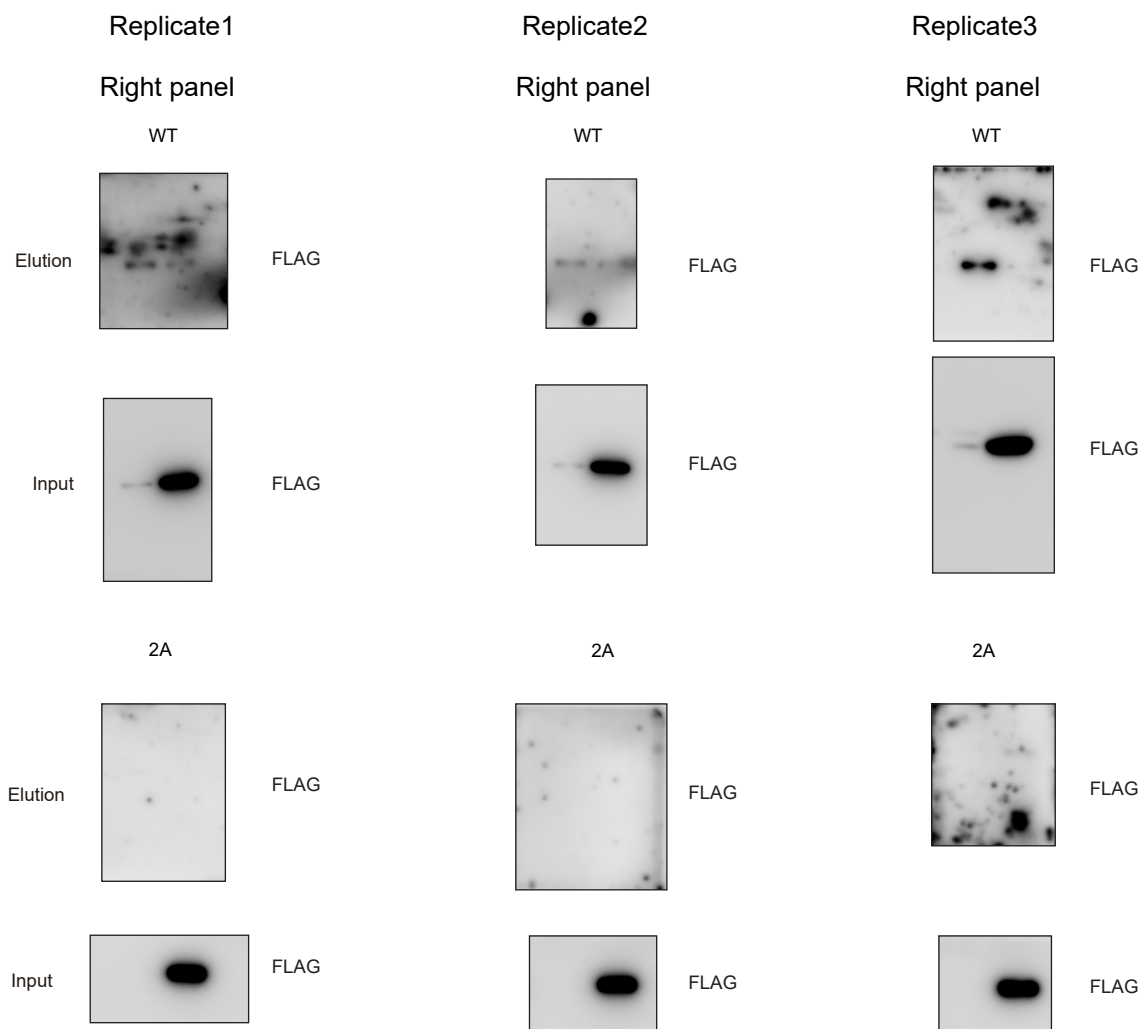

**Fig. 3e**

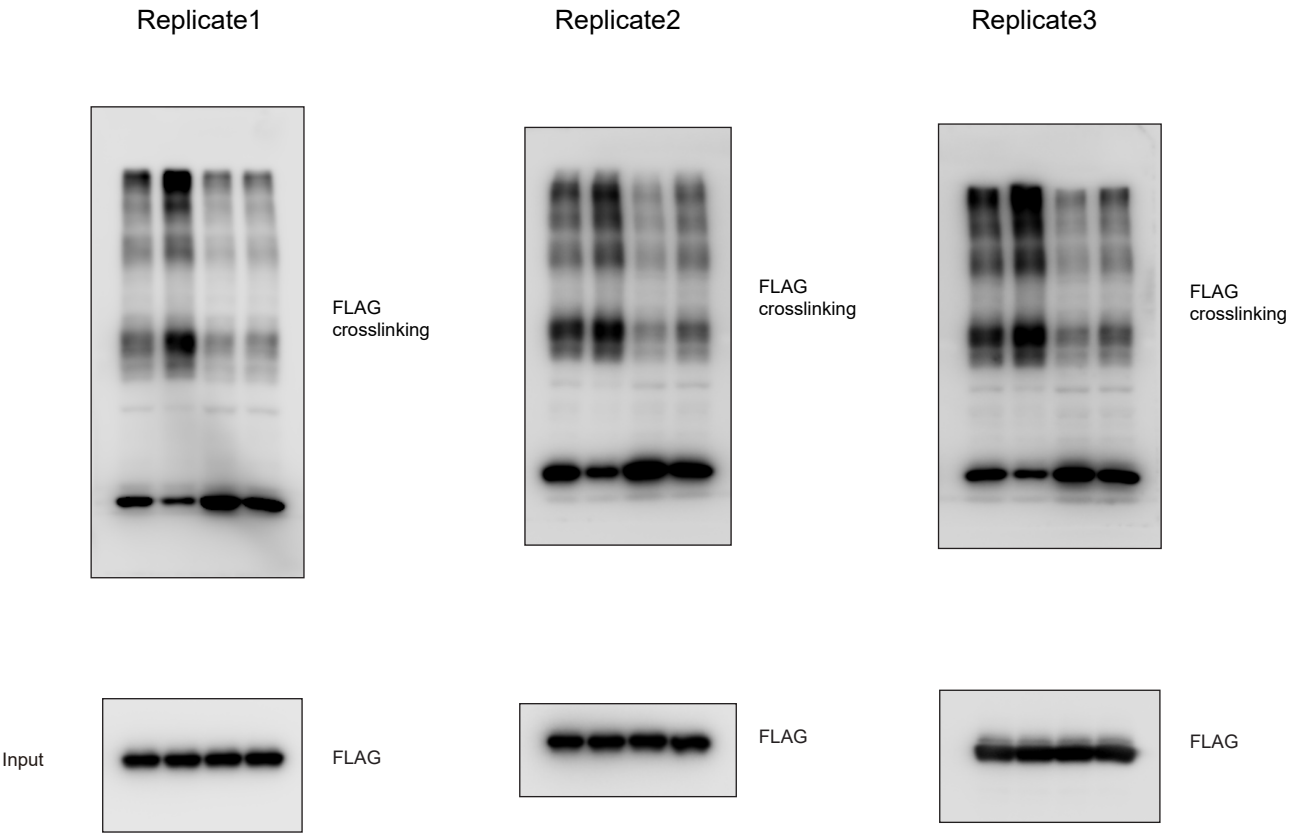

Supplement: Supplementary file 6 — Unprocessed western blots [file 41589_2023_1354_MOESM6_ESM.pdf]

Fig. 4a

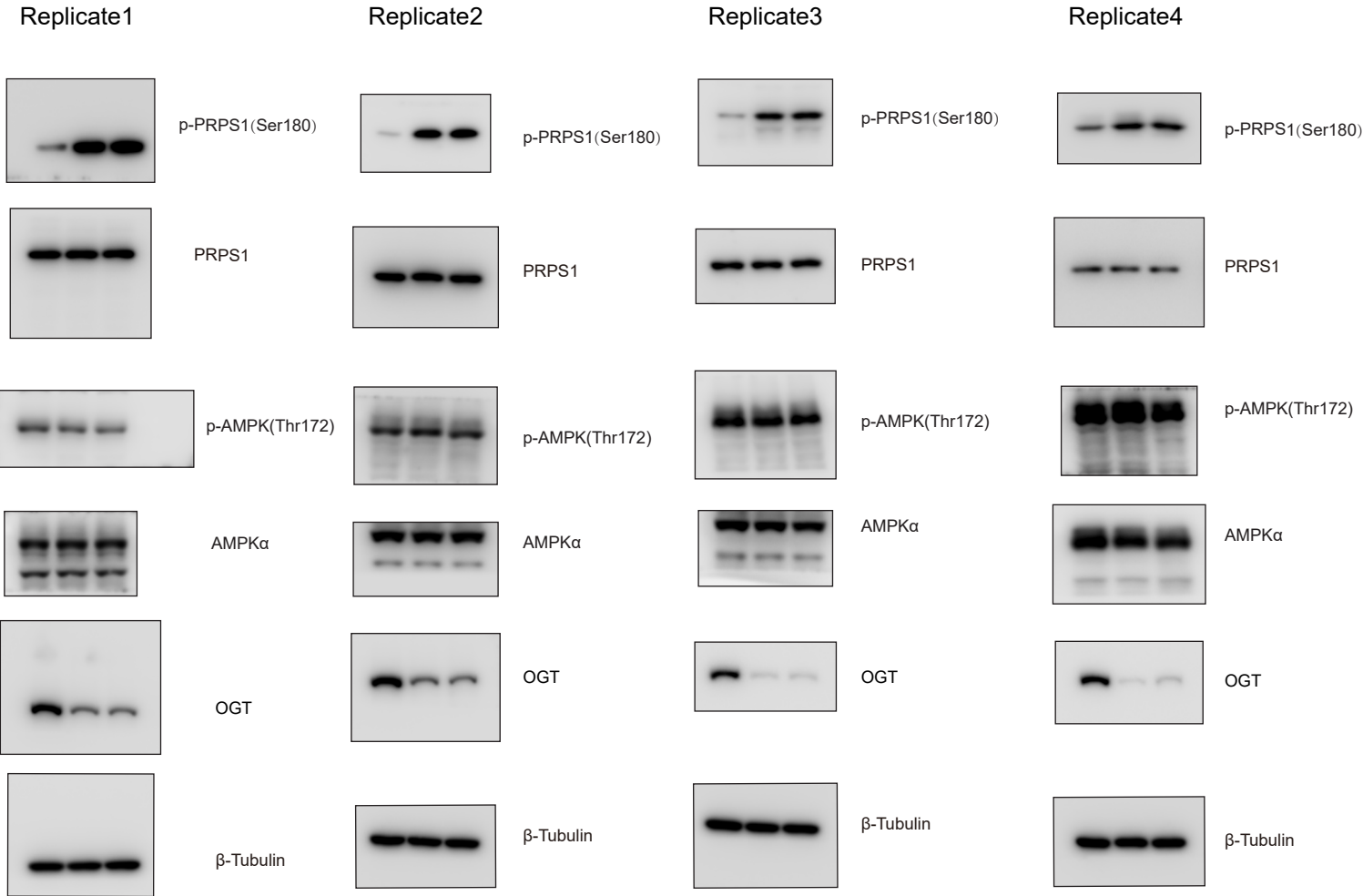

Fig. 4b

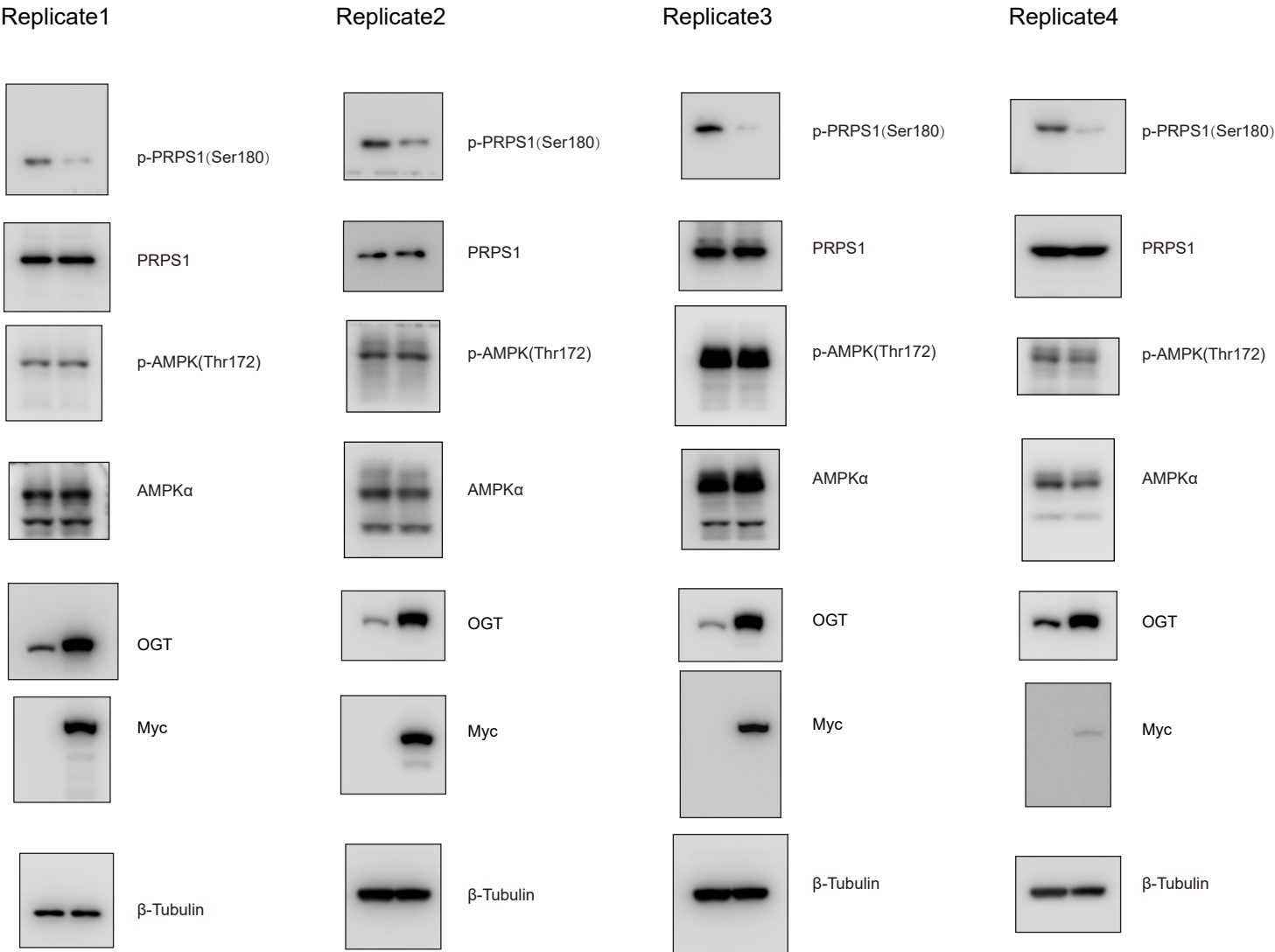

Fig. 4c

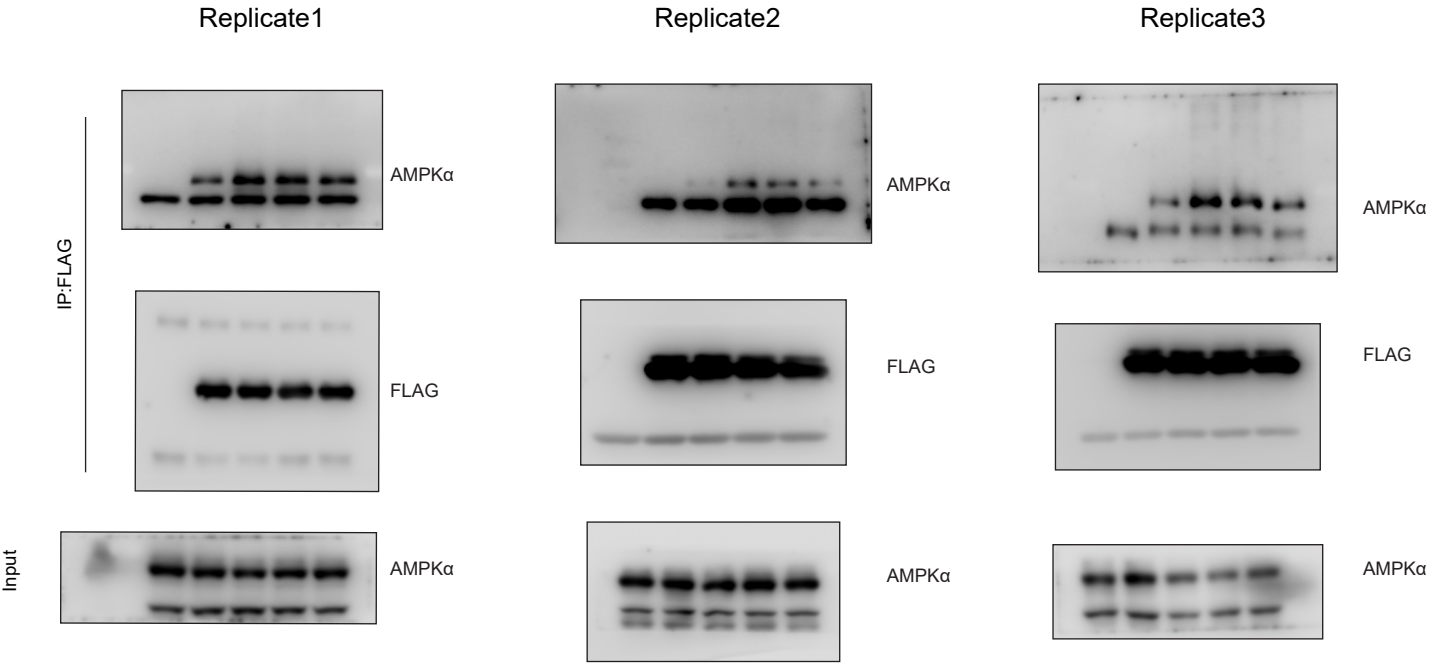

Fig. 4d

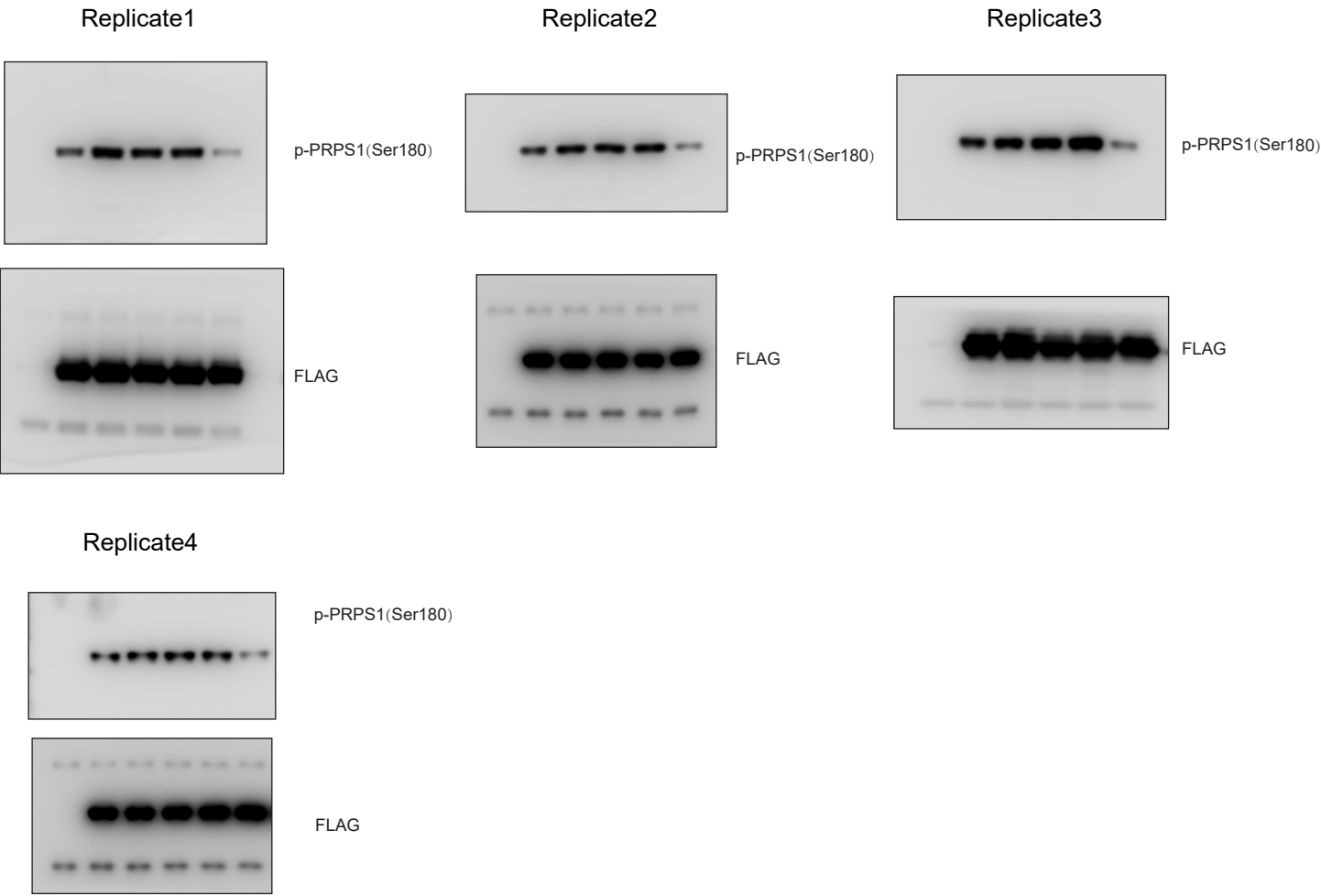

Fig. 4e

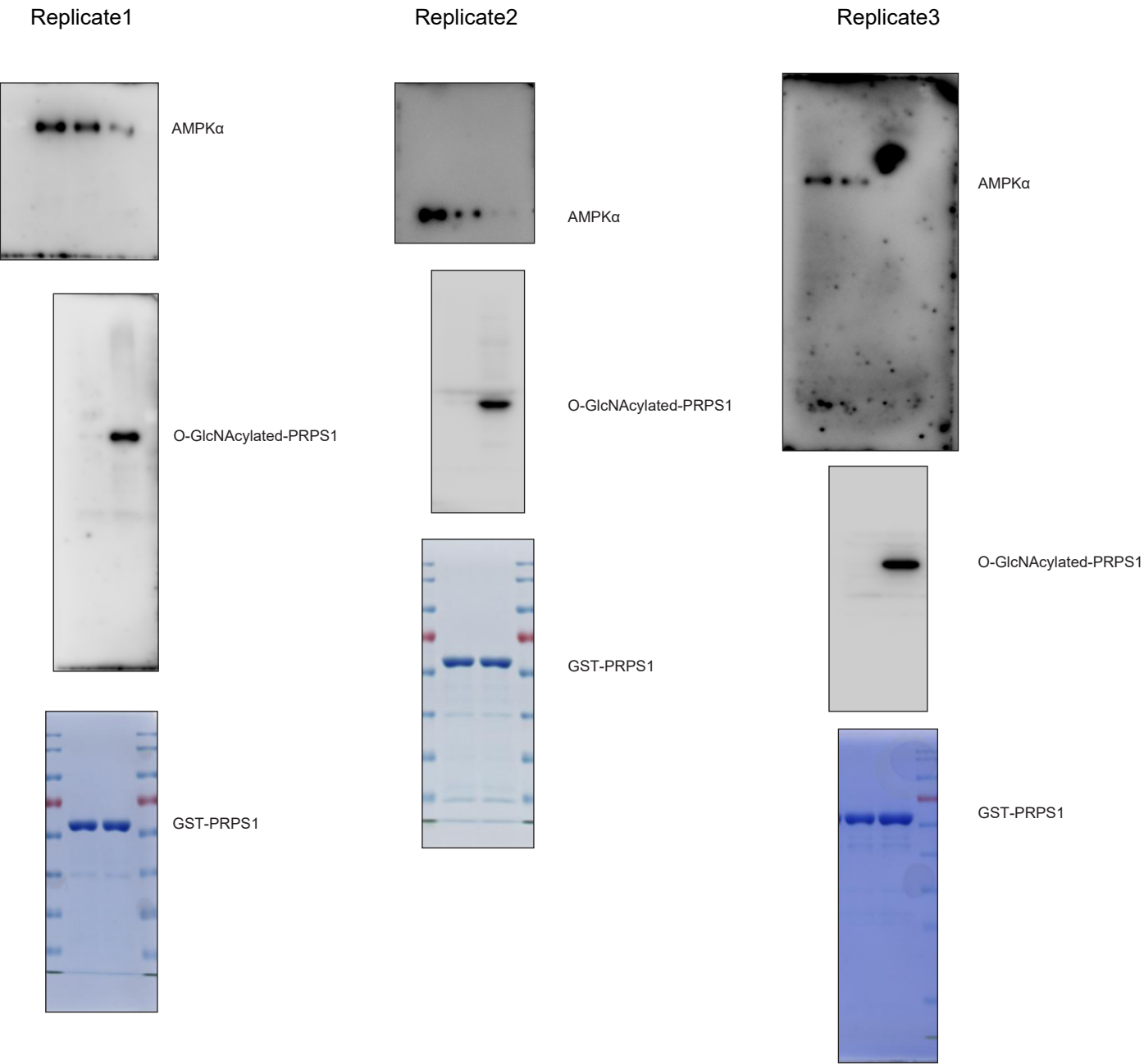

Fig. 4f

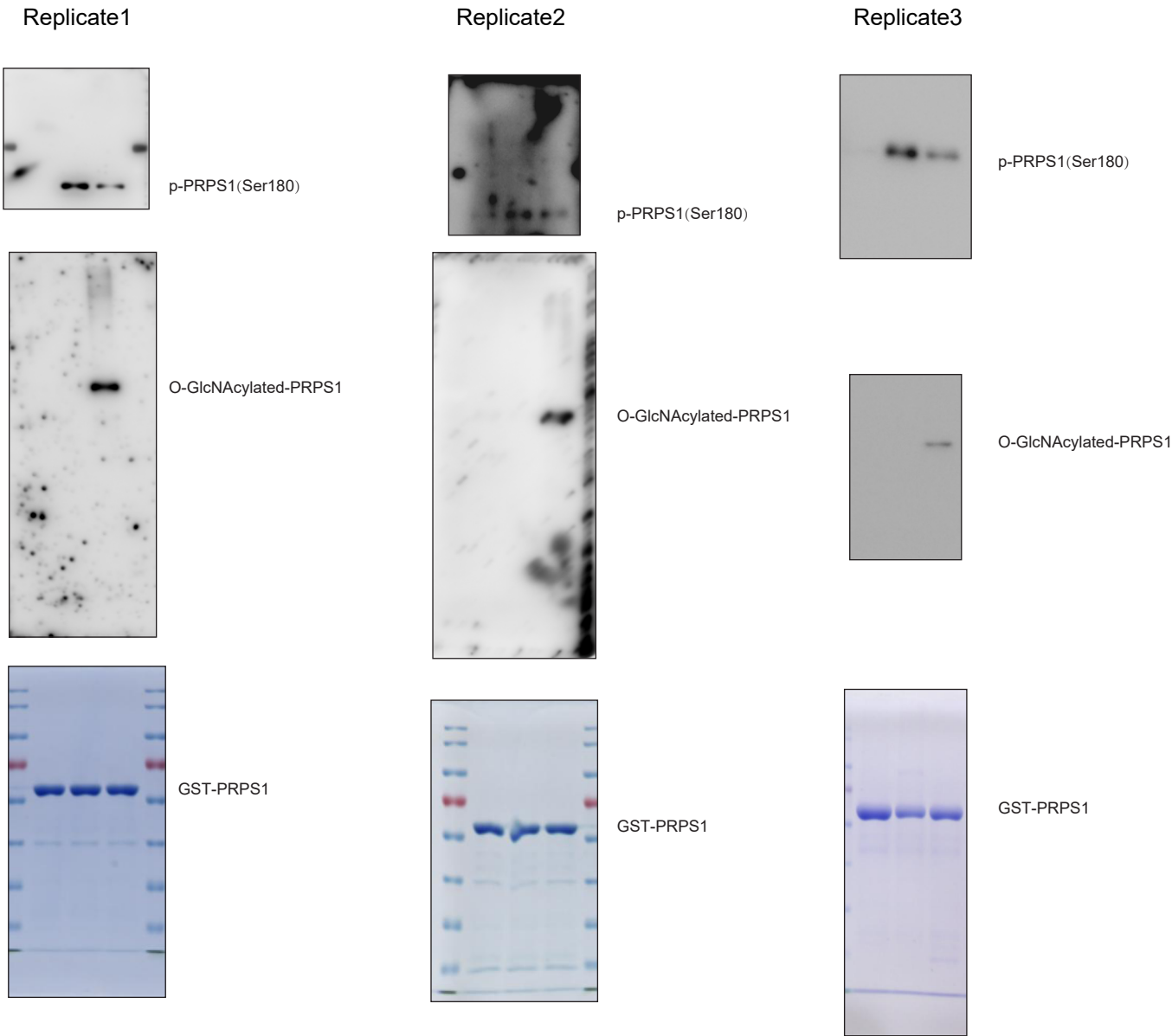

Fig. 4h

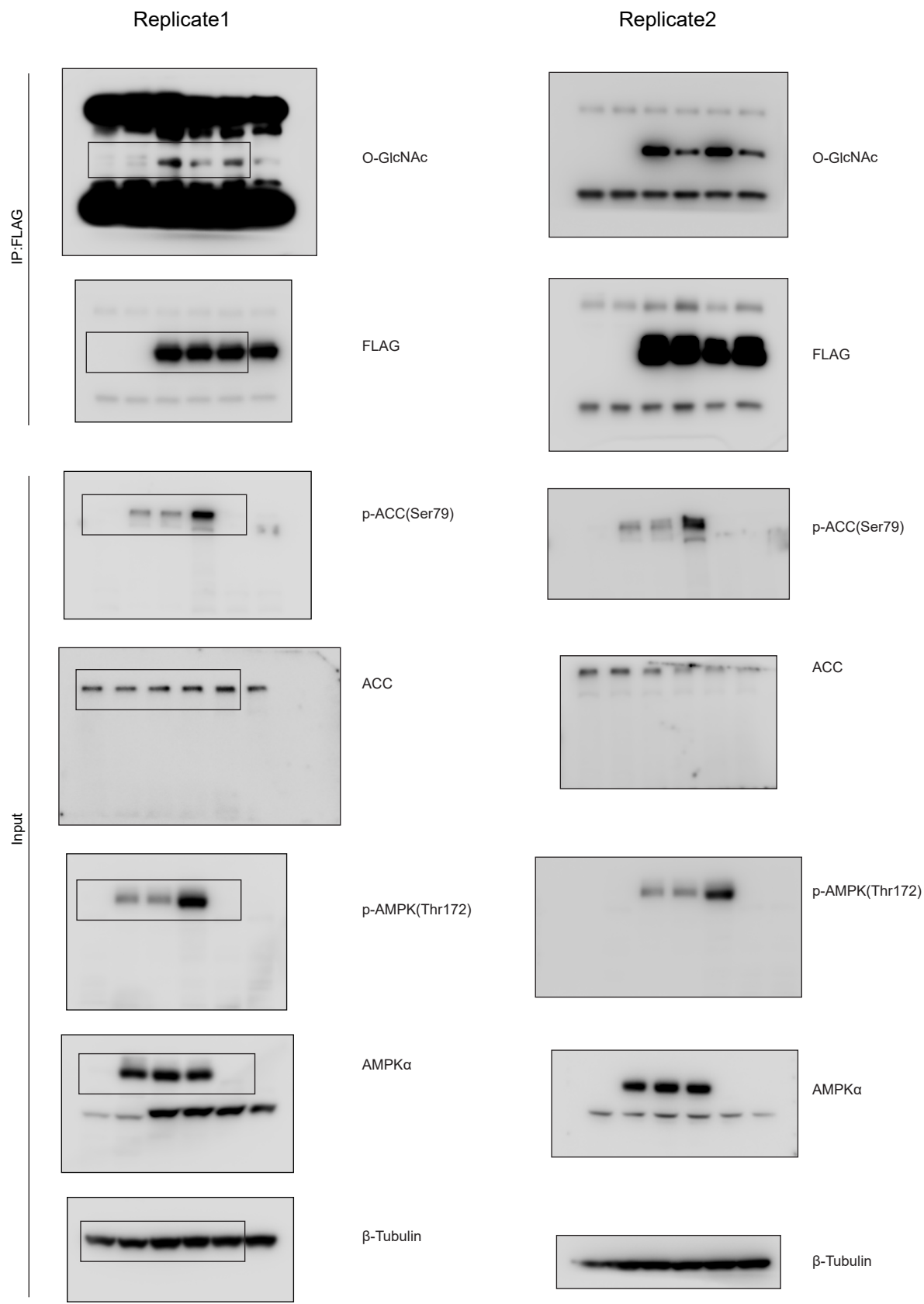

**Fig. 4i**

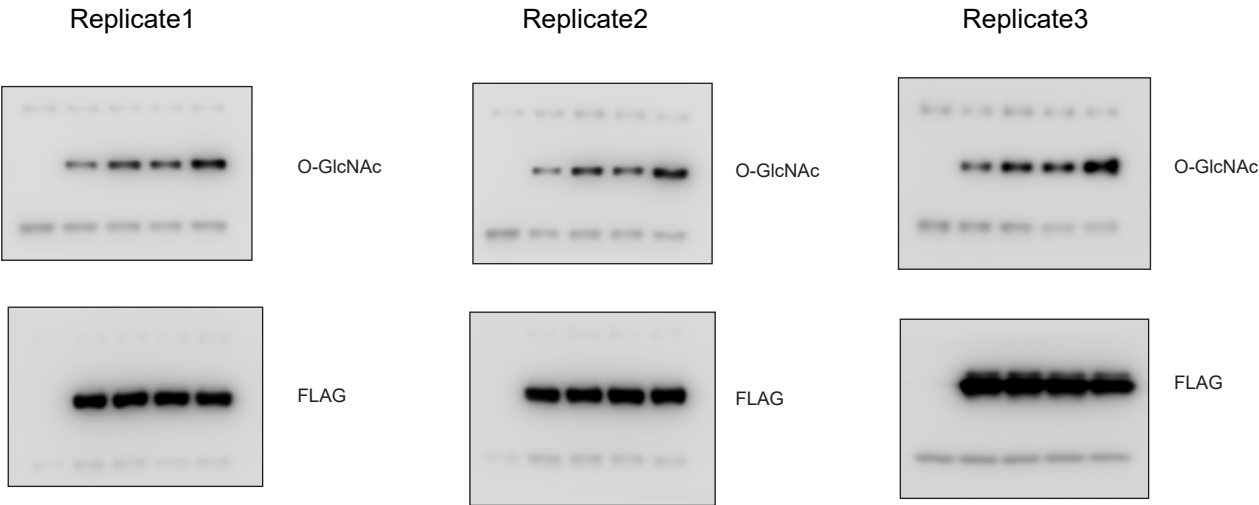

Fig. 4j

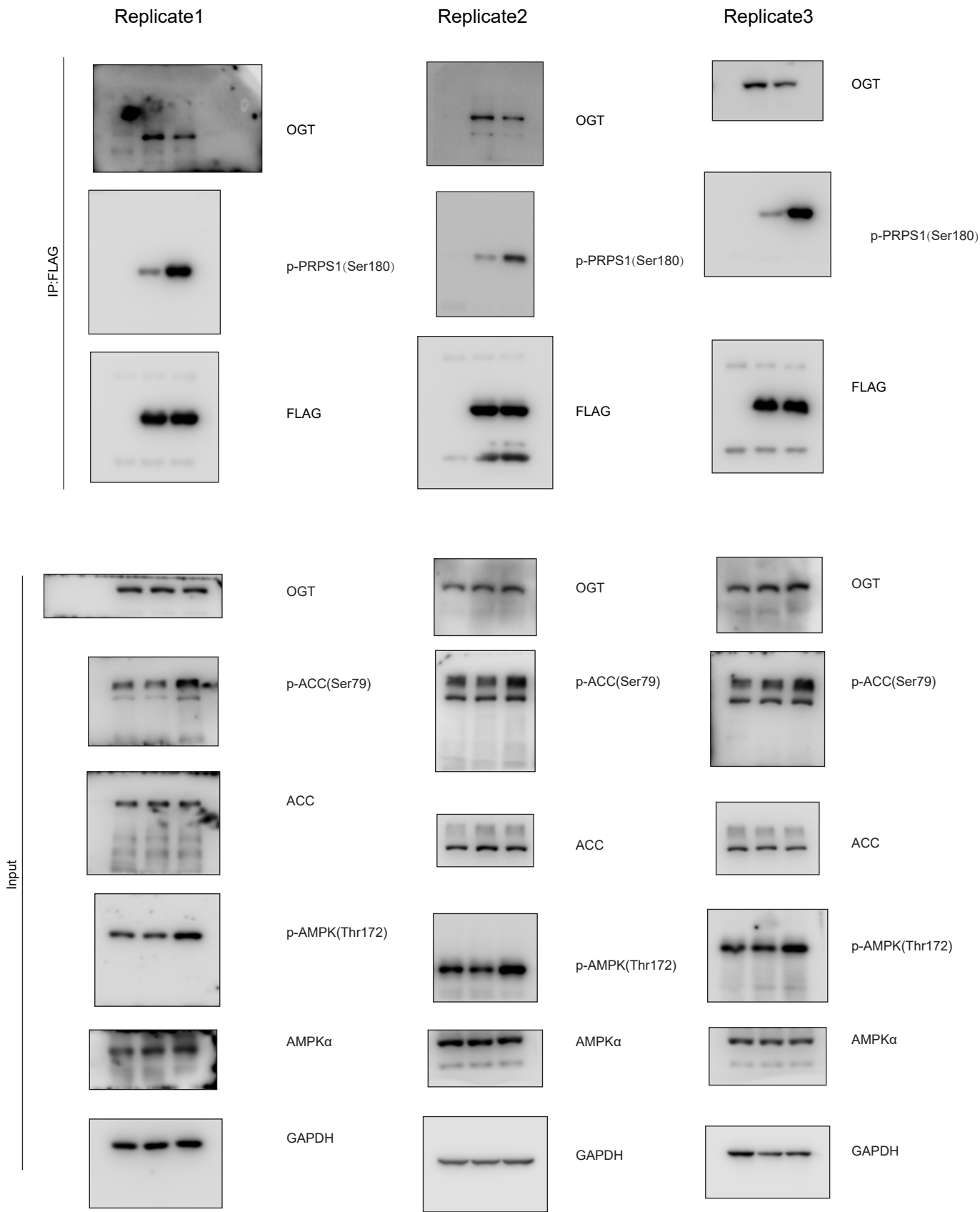

Fig. 4k

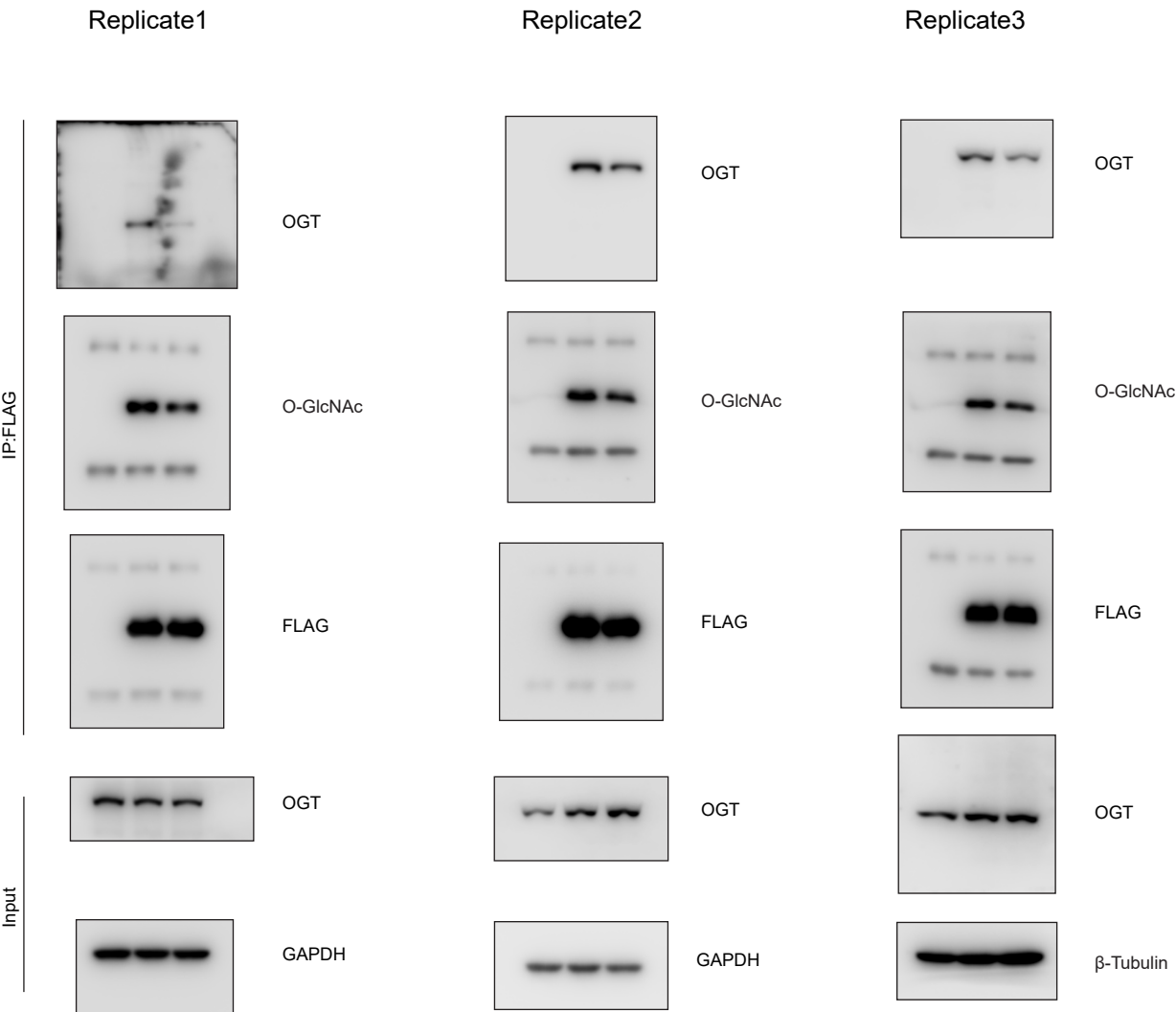

Fig. 4I

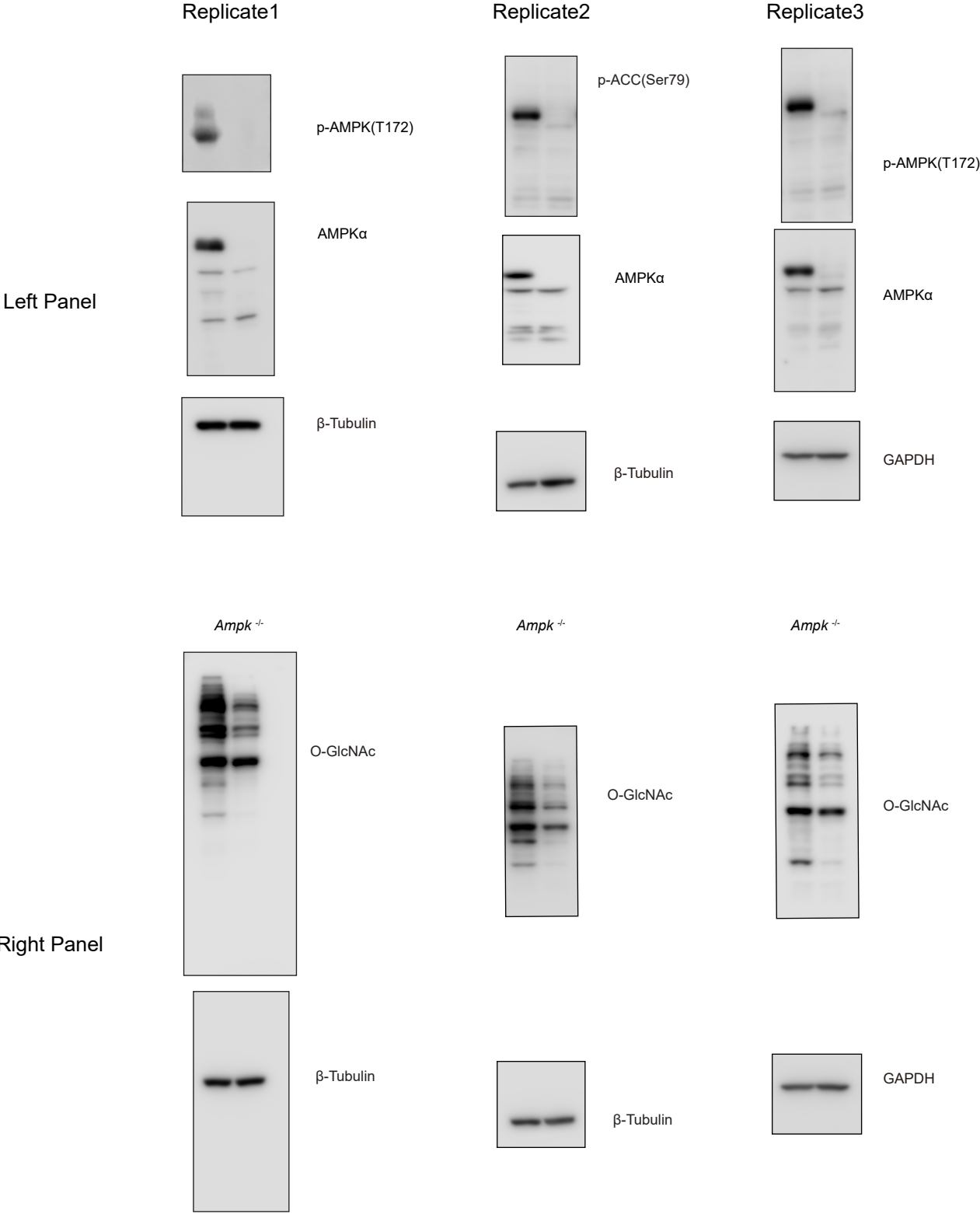

Fig. 4m

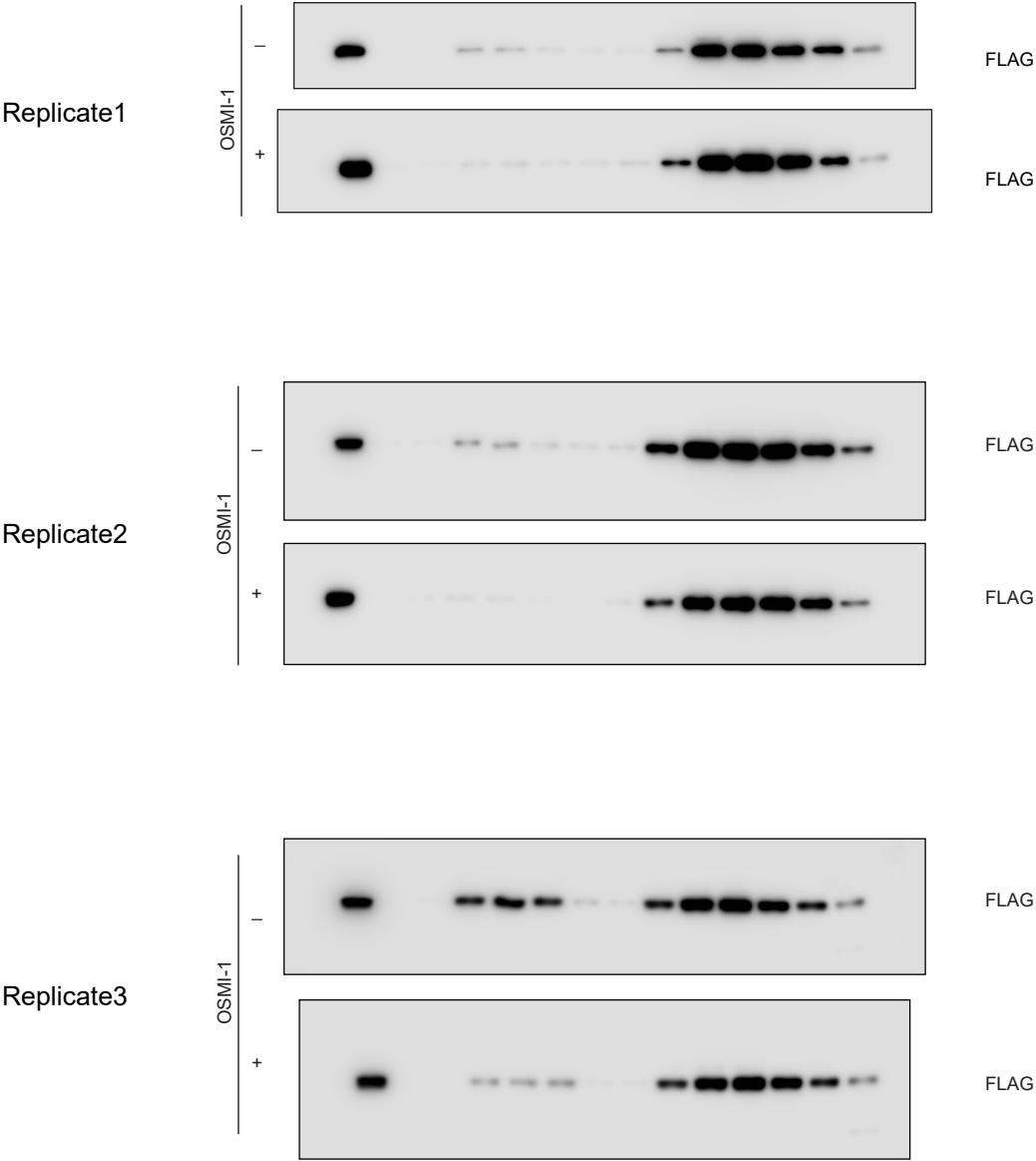

**Fig. 4n**

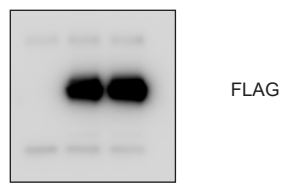

Supplement: Supplementary file 8 — Unprocessed western blots [file 41589_2023_1354_MOESM8_ESM.pdf]

**Fig. 5a**

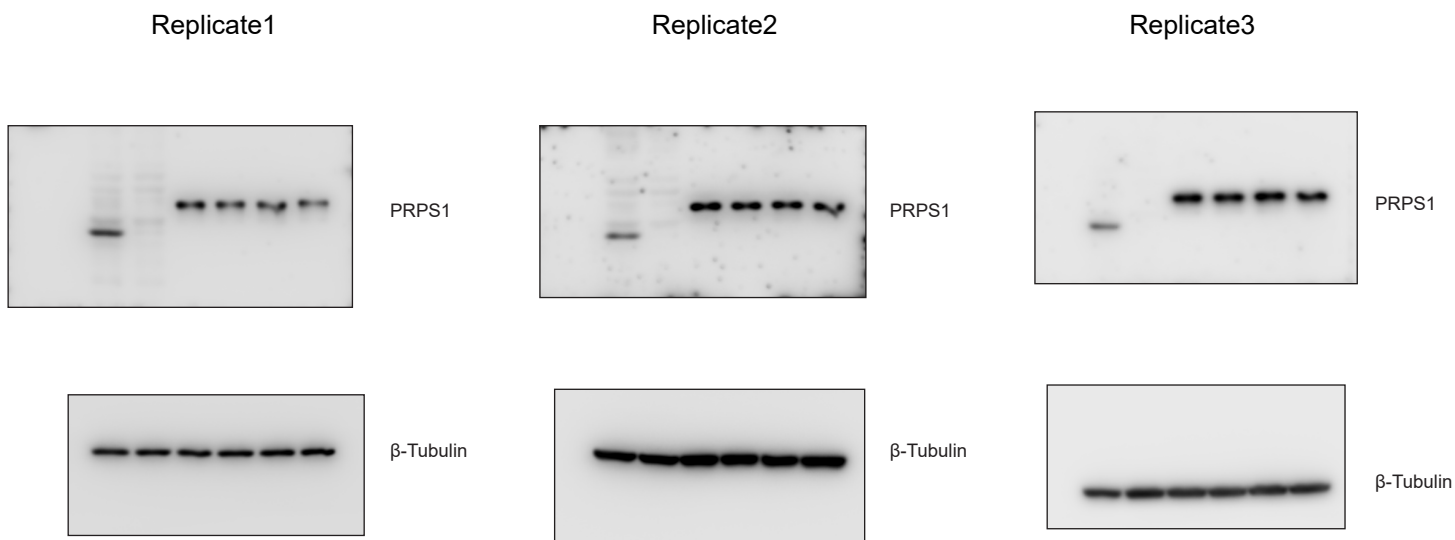

**Fig. 5h**

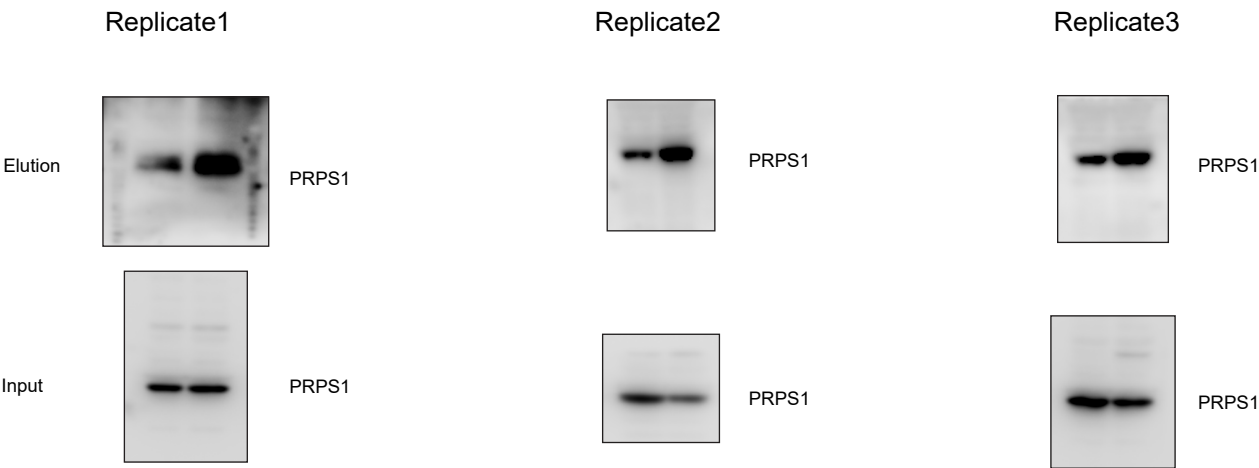

**Fig. 5i**

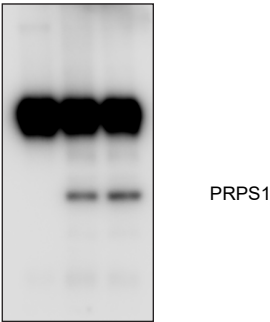

**Fig. 5j**

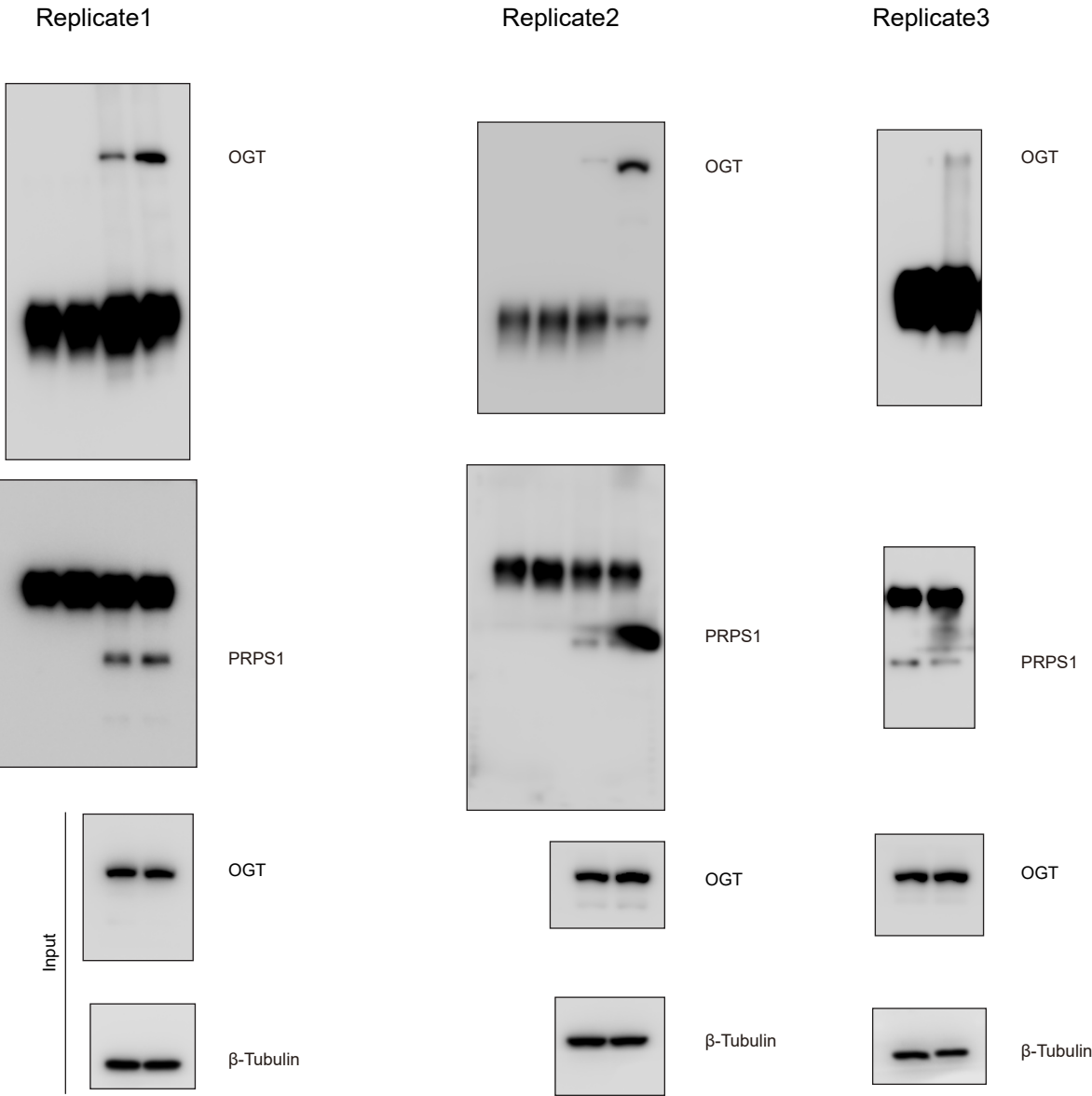

Supplement: Supplementary file 10 — Unprocessed western blots [file 41589_2023_1354_MOESM10_ESM.pdf]

Fig. 6a

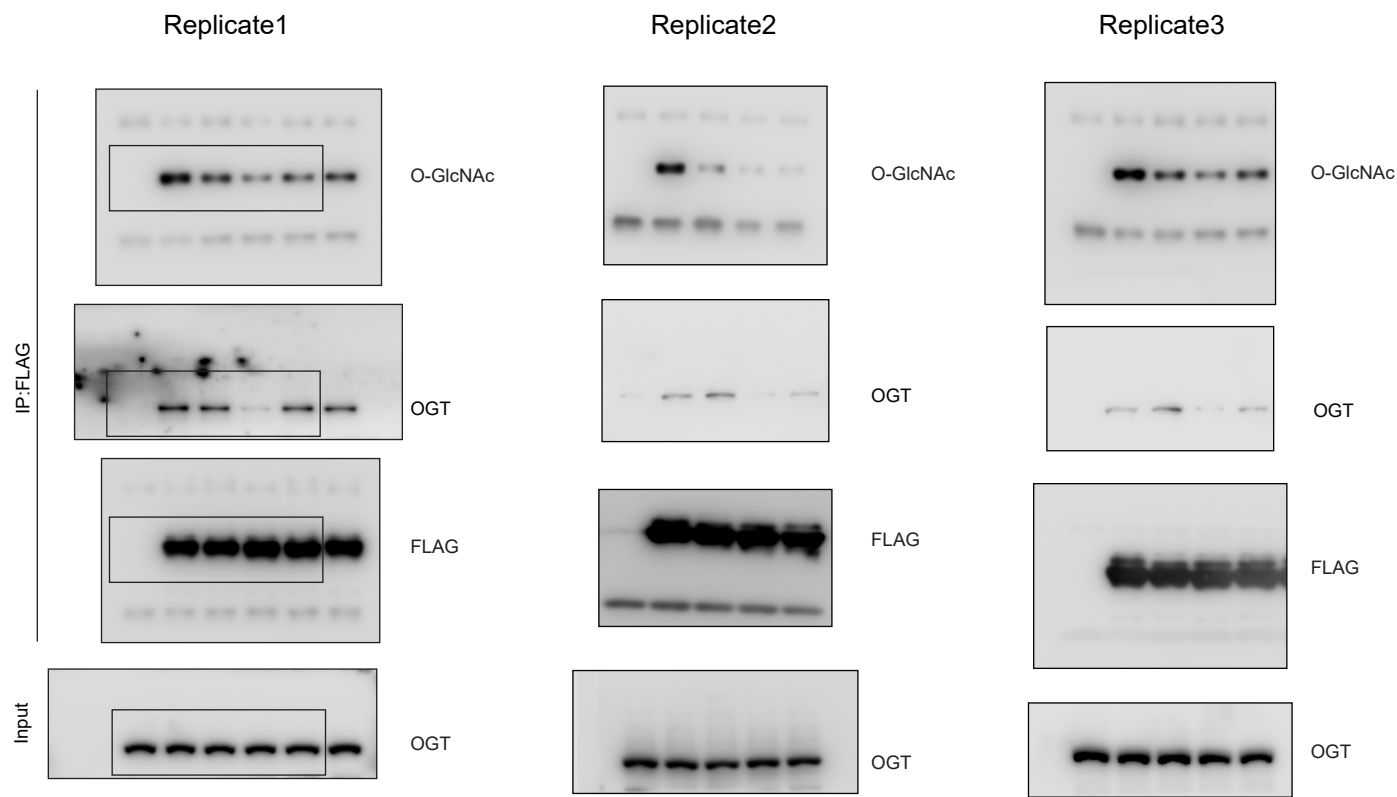

Fig. 6b

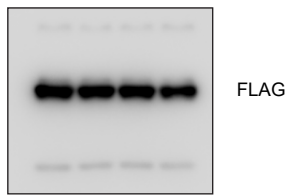

**Fig. 6c**

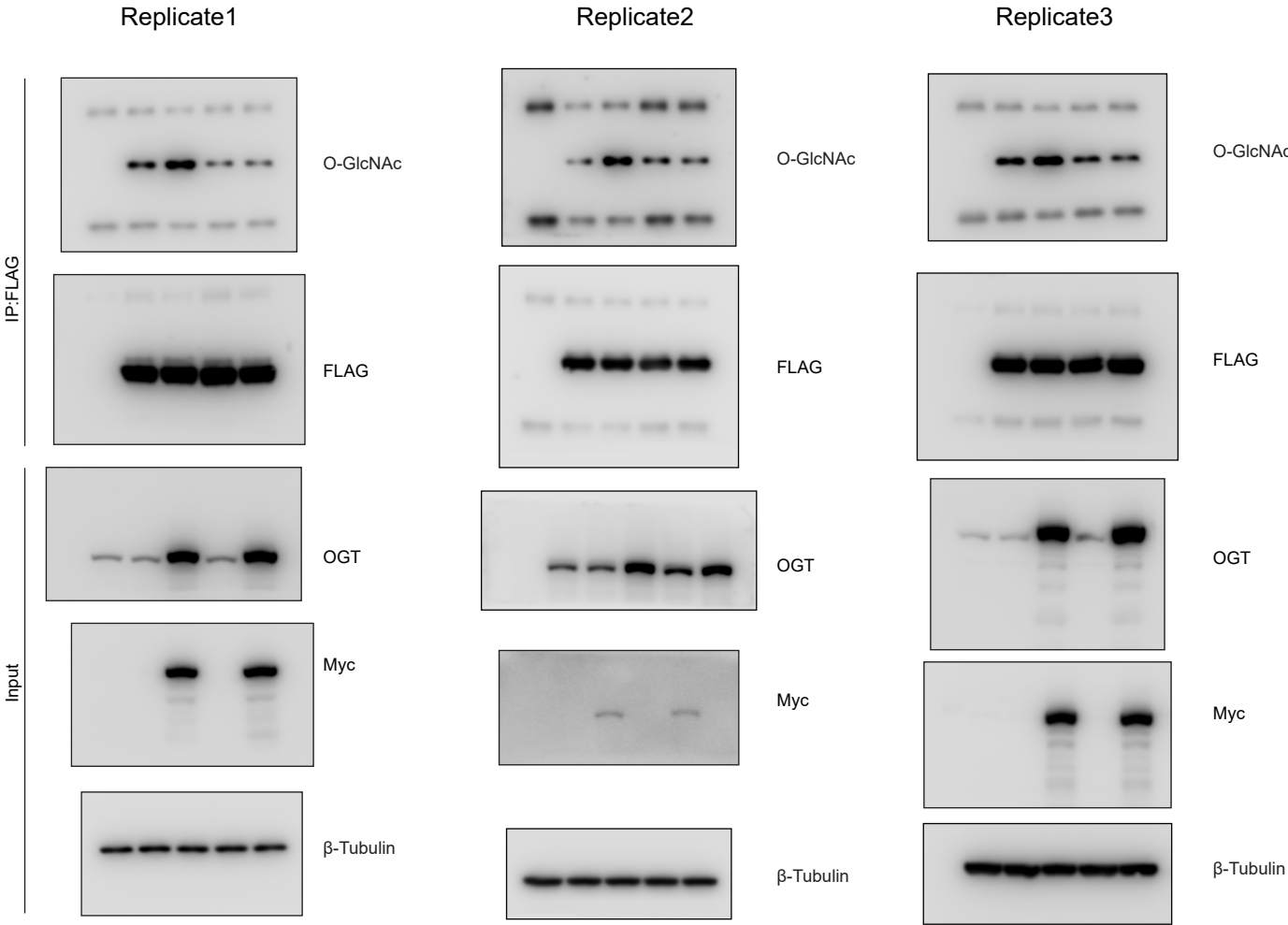

**Fig. 6d**

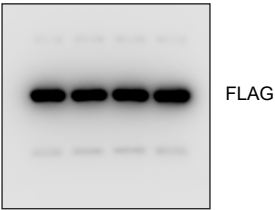

Fig. 6f

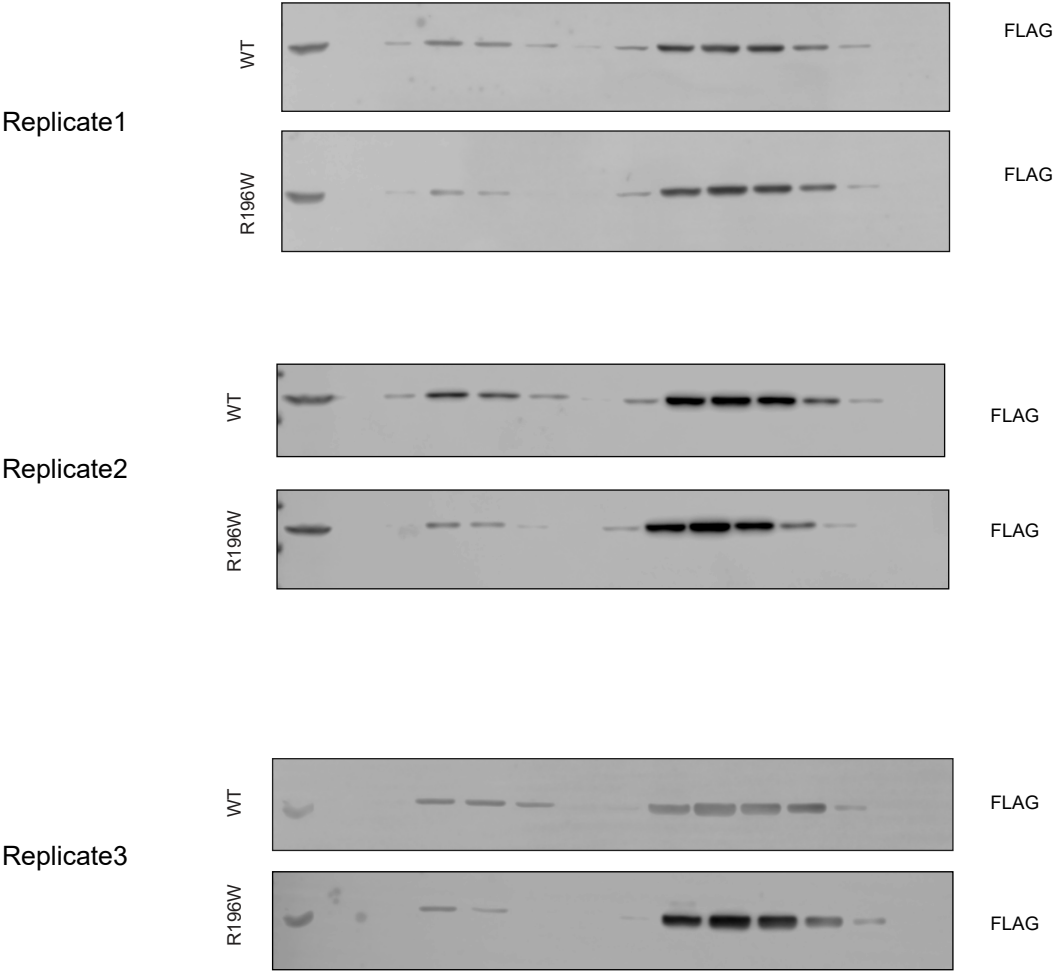

Fig. 6g

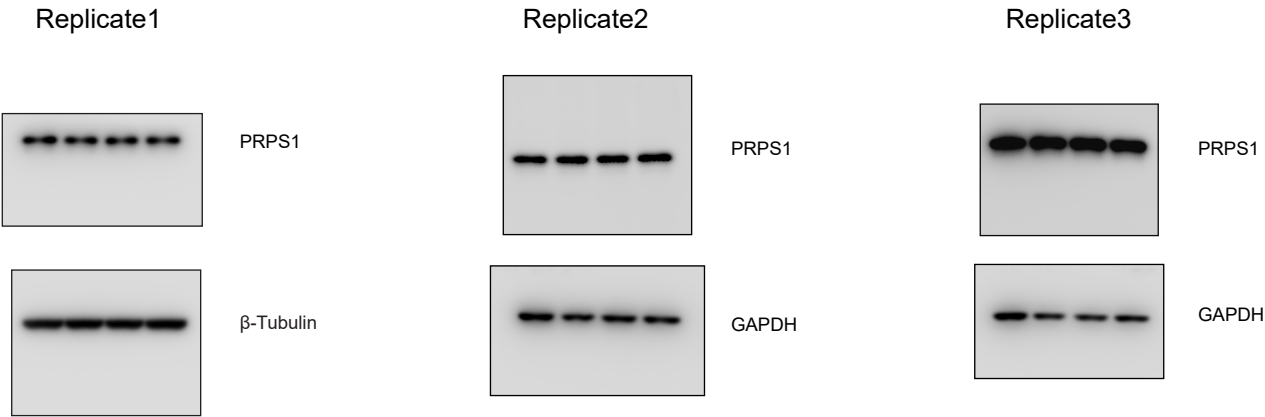

Fig. 6h

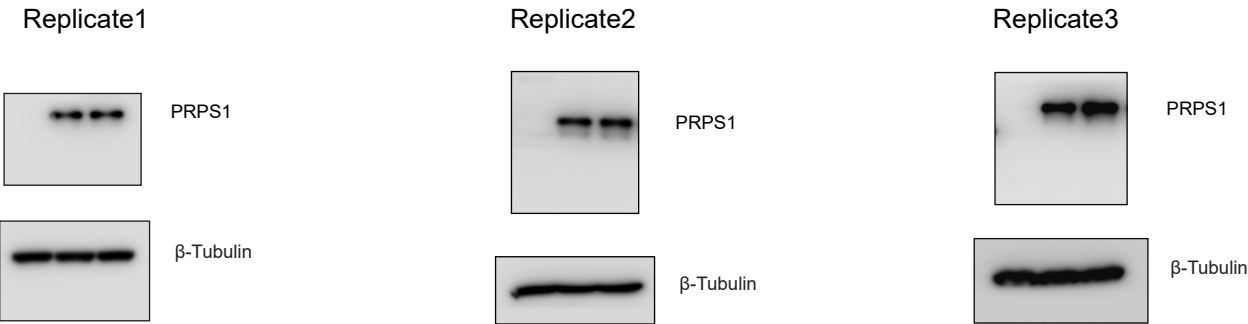

**Fig. 6i**

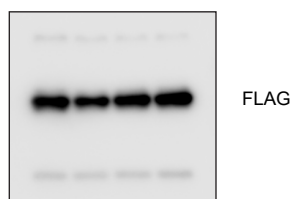

**Fig. 6j**

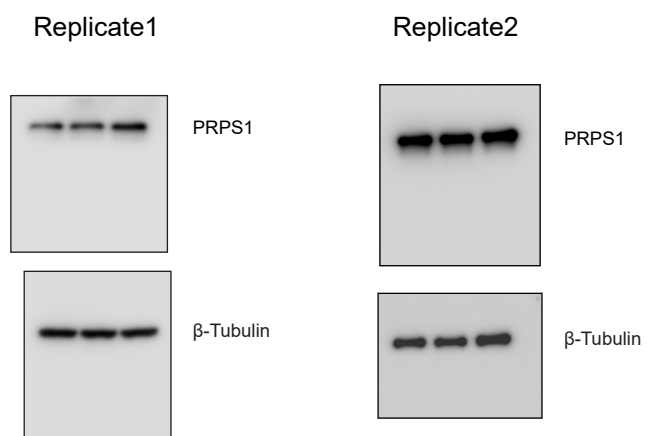

Supplement: Supplementary file 12 — Unprocessed western blots [file 41589_2023_1354_MOESM12_ESM.pdf]

Extended Data Fig. 3a

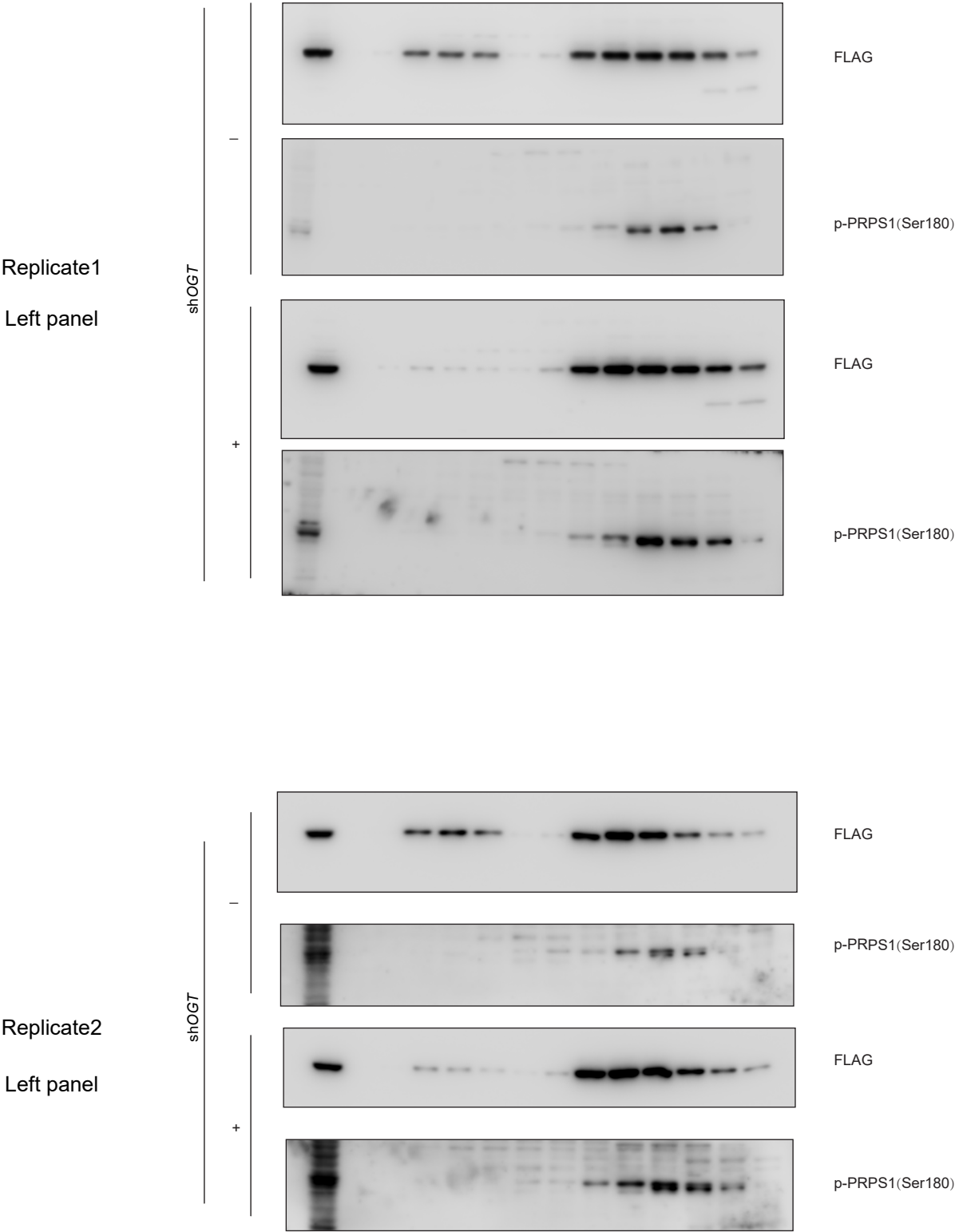

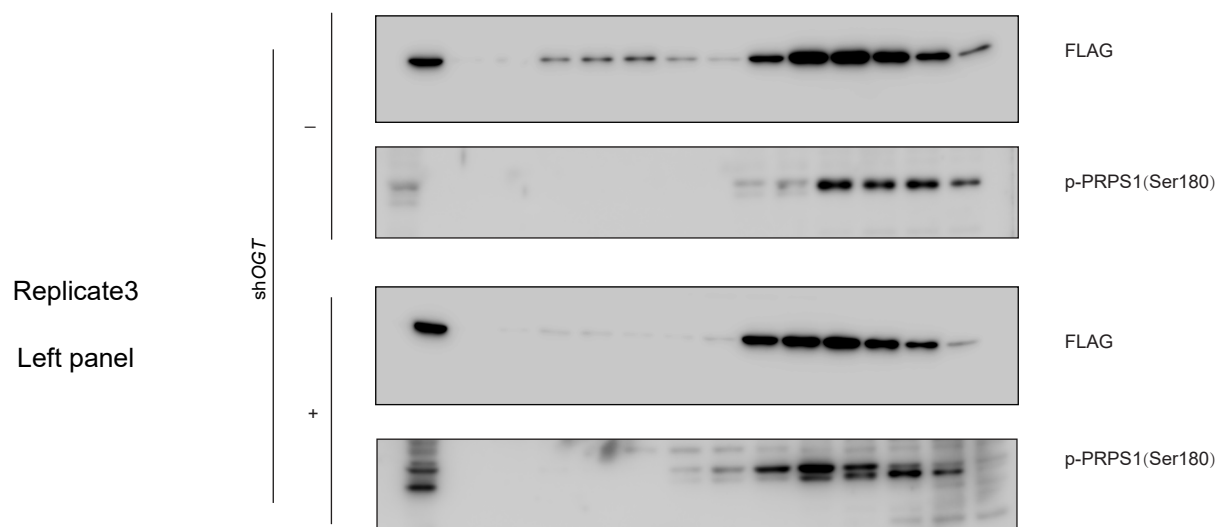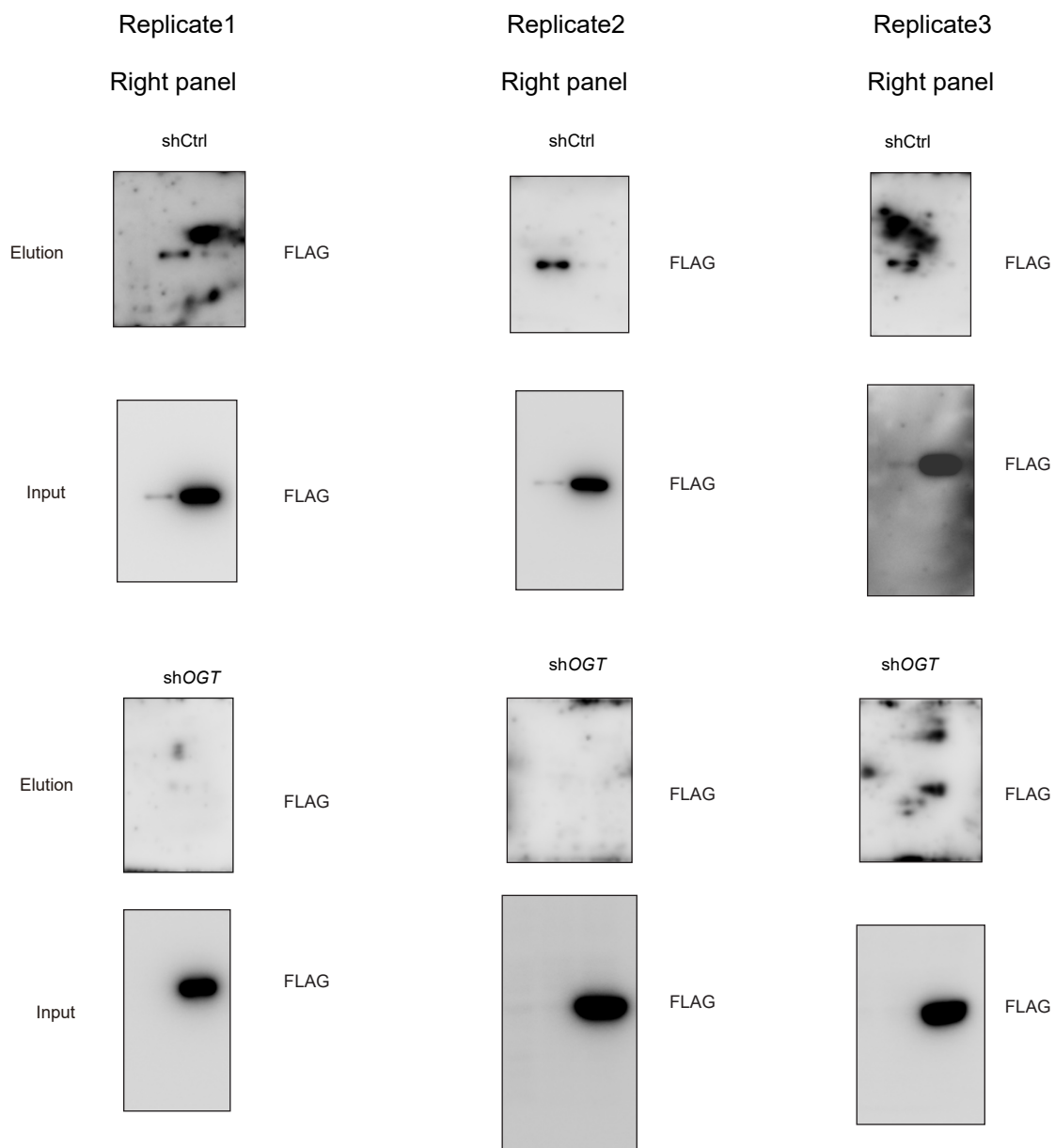

Extended Data Fig. 3b

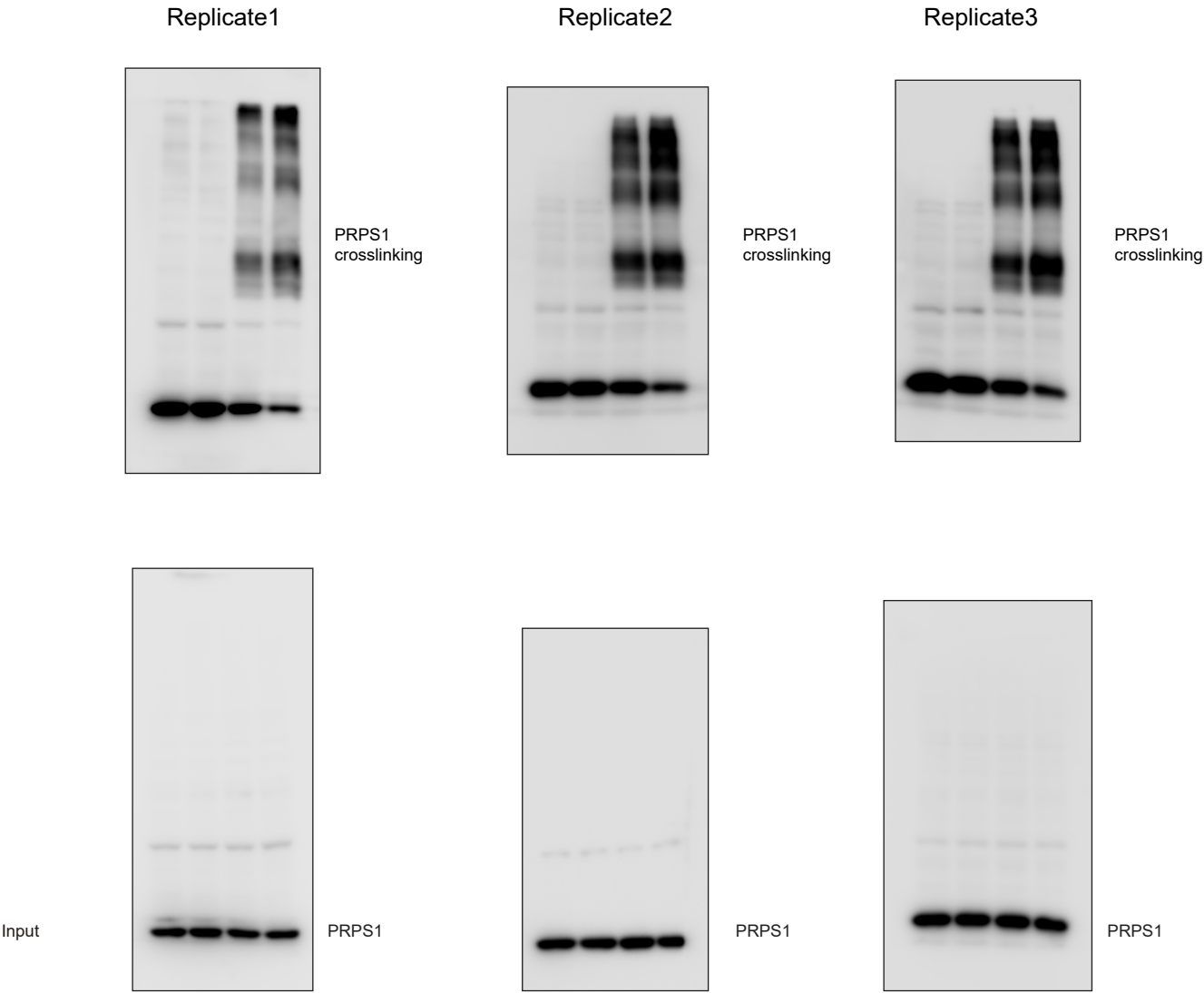

Extended Data Fig. 3c

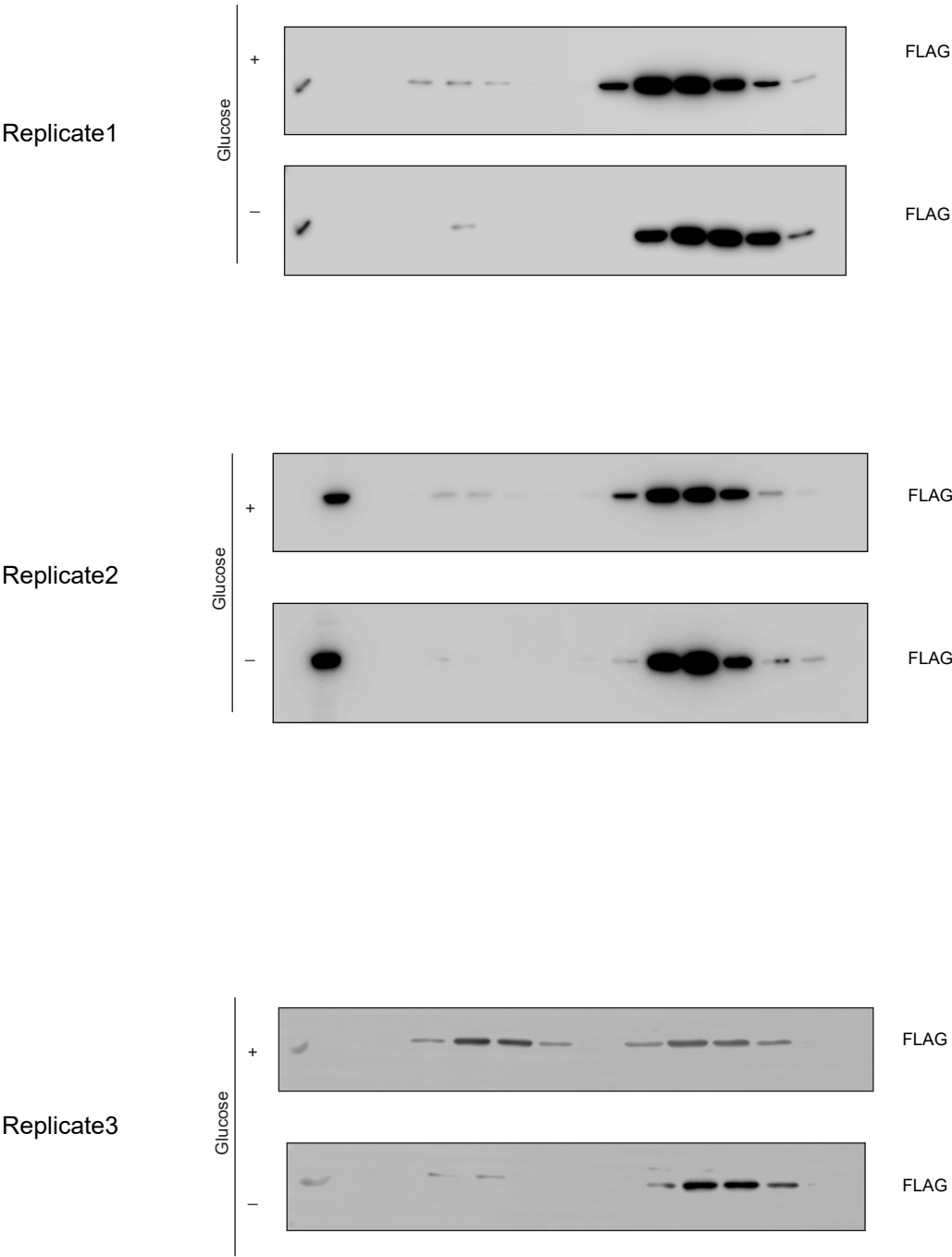

Extended Data Fig. 3d

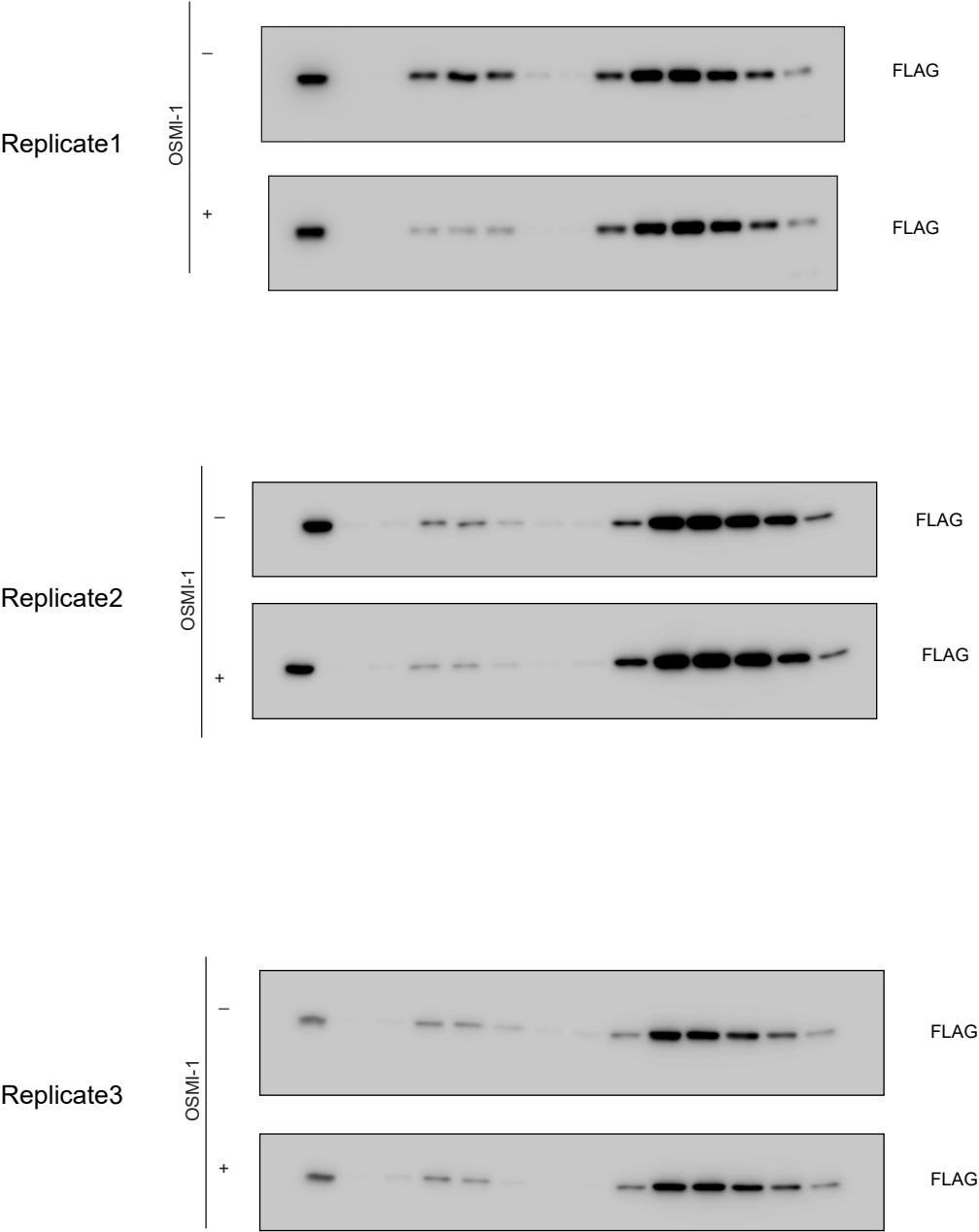

Supplement: Supplementary file 17 — Unprocessed western blots [file 41589_2023_1354_MOESM17_ESM.pdf]

Extended Data Fig. 5f

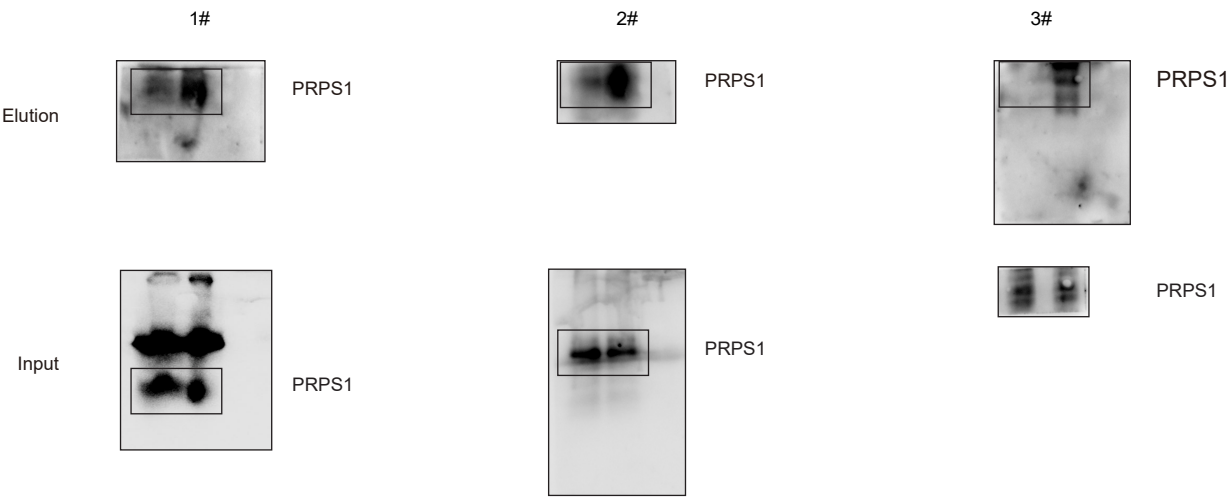

Supplement: Supplementary file 21 — Unprocessed western blots [file 41589_2023_1354_MOESM21_ESM.pdf]
