# Supplementary material for: Direct stimulation of de novo nucleotide synthesis by O-GlcNAcylation
Source: Nat Chem Biol. 2023 Jun 12;20(1):19–29. doi: 10.1038/s41589-023-01354-x (PMC10746546; doi:10.1038/s41589-023-01354-x)

Extended Data Fig. 1c

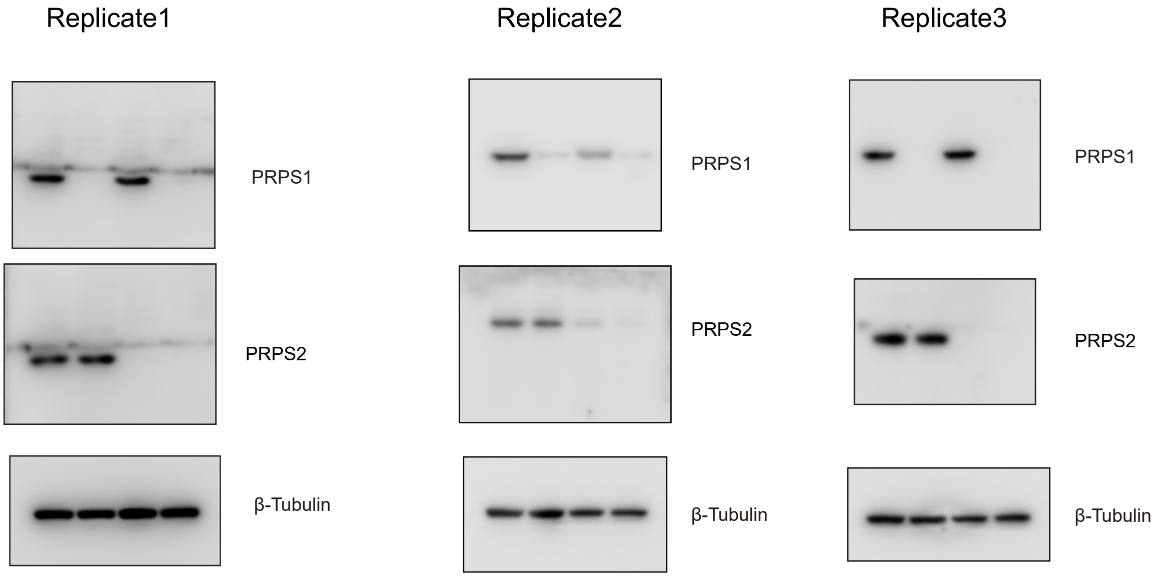

Extended Data Fig. 1d

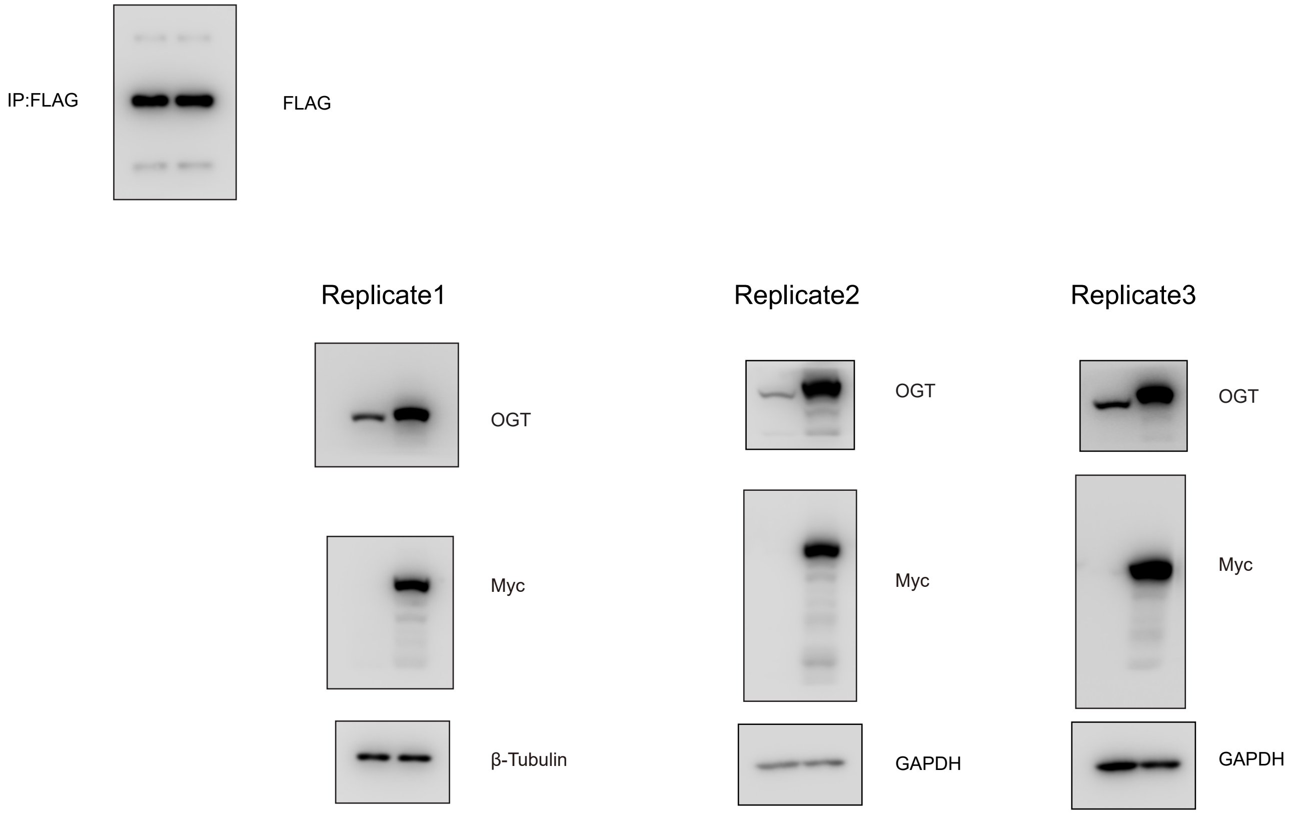

Extended Data Fig. 1e

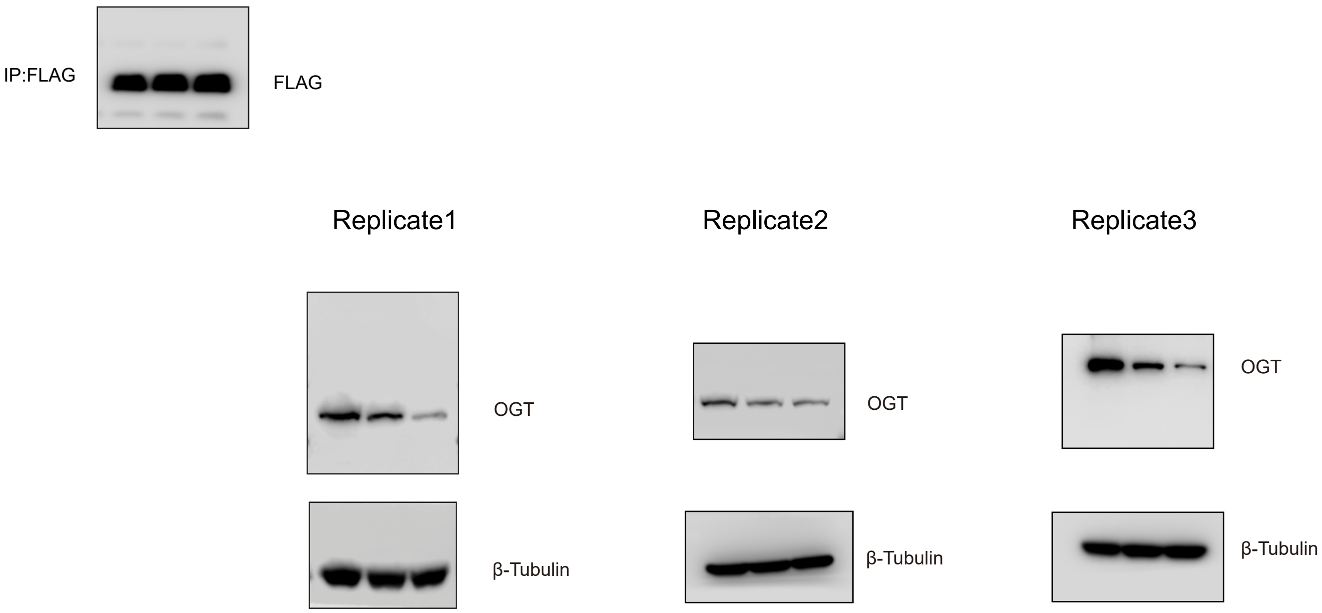

Extended Data Fig. 1f

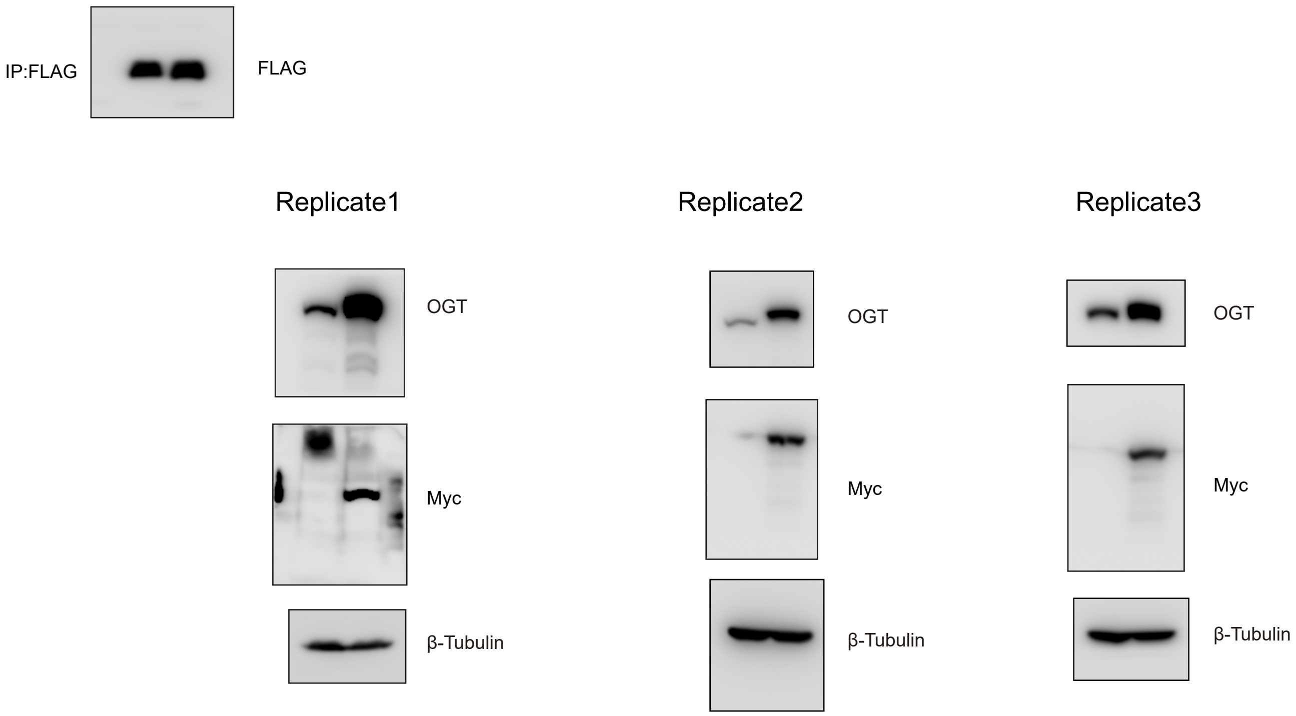

Extended Data Fig. 1g

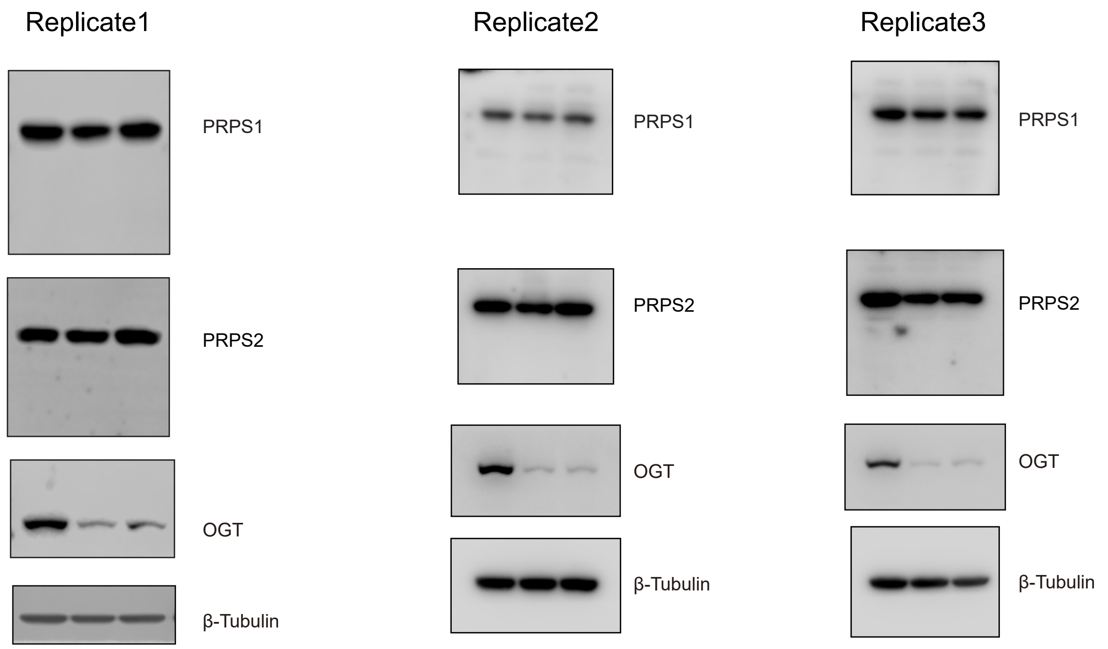

Extended Data Fig. 1h

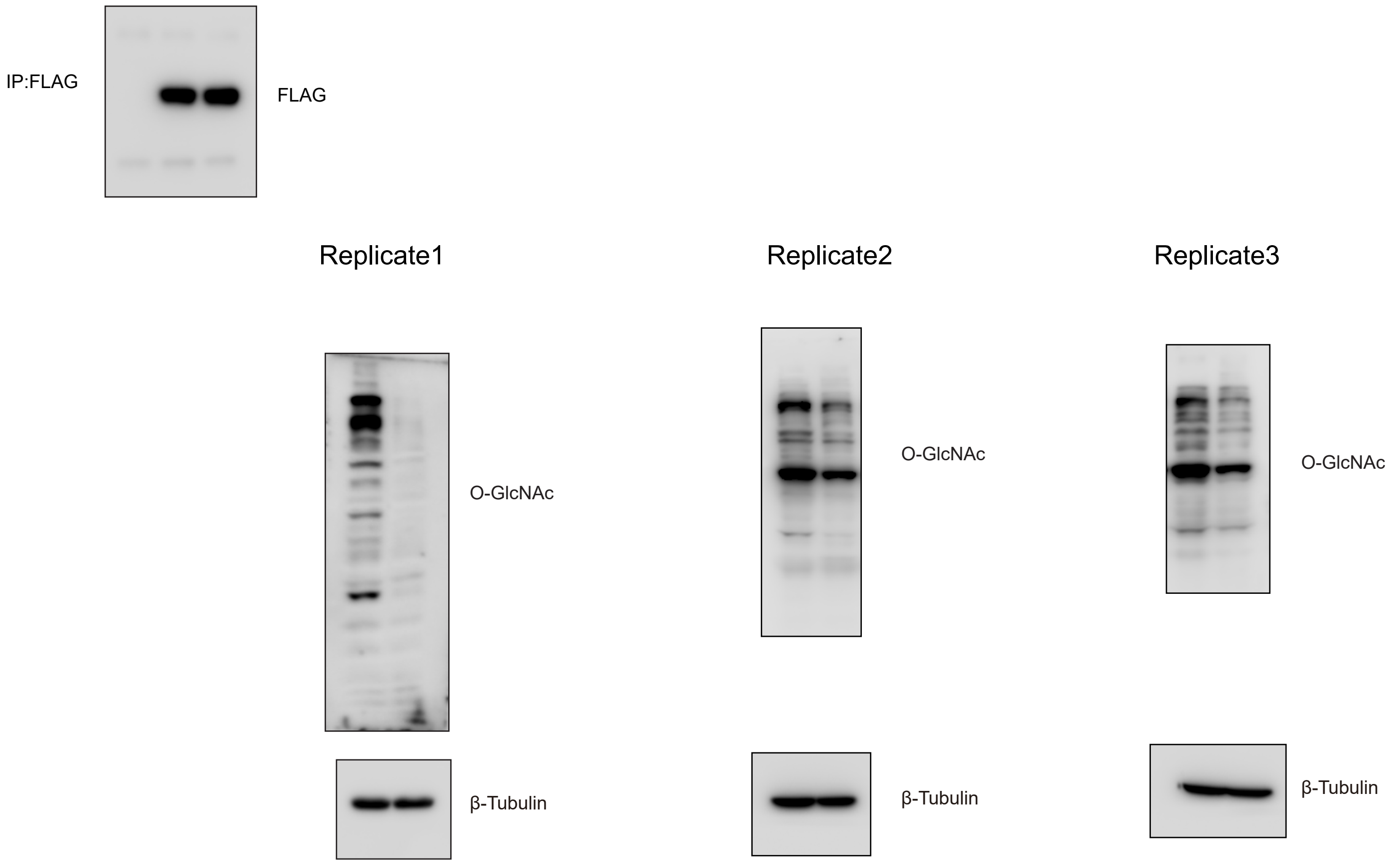

Extended Data Fig. 1i

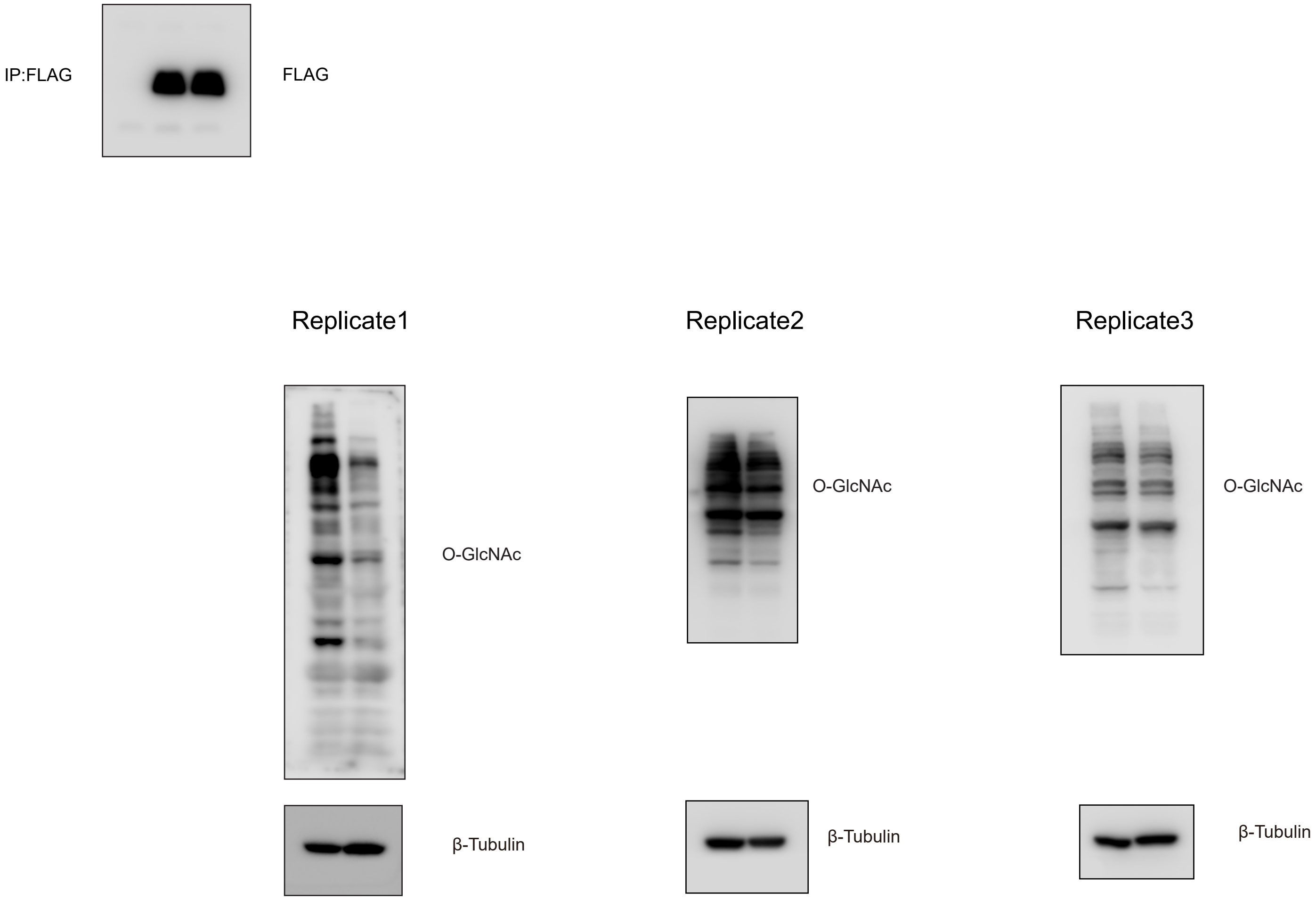

Supplement: Supplementary file 14 — Unprocessed western blots [file 41589_2023_1354_MOESM14_ESM.pdf]
