# Supplementary material for: Direct stimulation of de novo nucleotide synthesis by O-GlcNAcylation
Source: Nat Chem Biol. 2023 Jun 12;20(1):19–29. doi: 10.1038/s41589-023-01354-x (PMC10746546; doi:10.1038/s41589-023-01354-x)

Extended Data Fig.2a

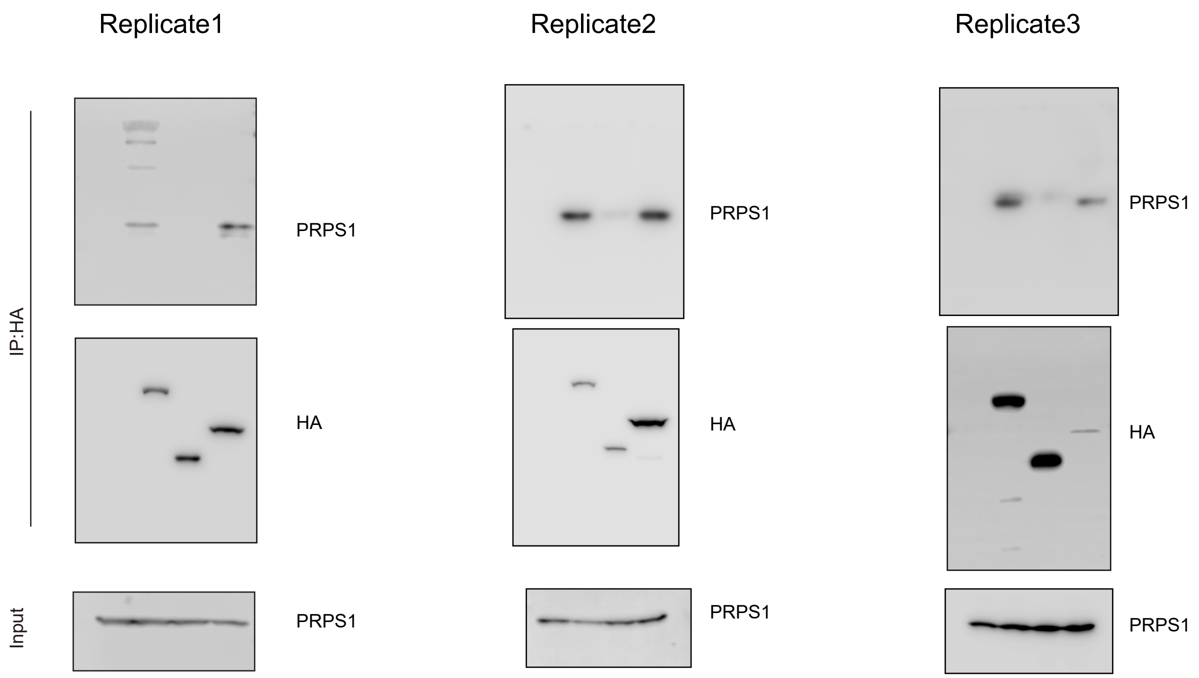

Extended Data Fig.2b

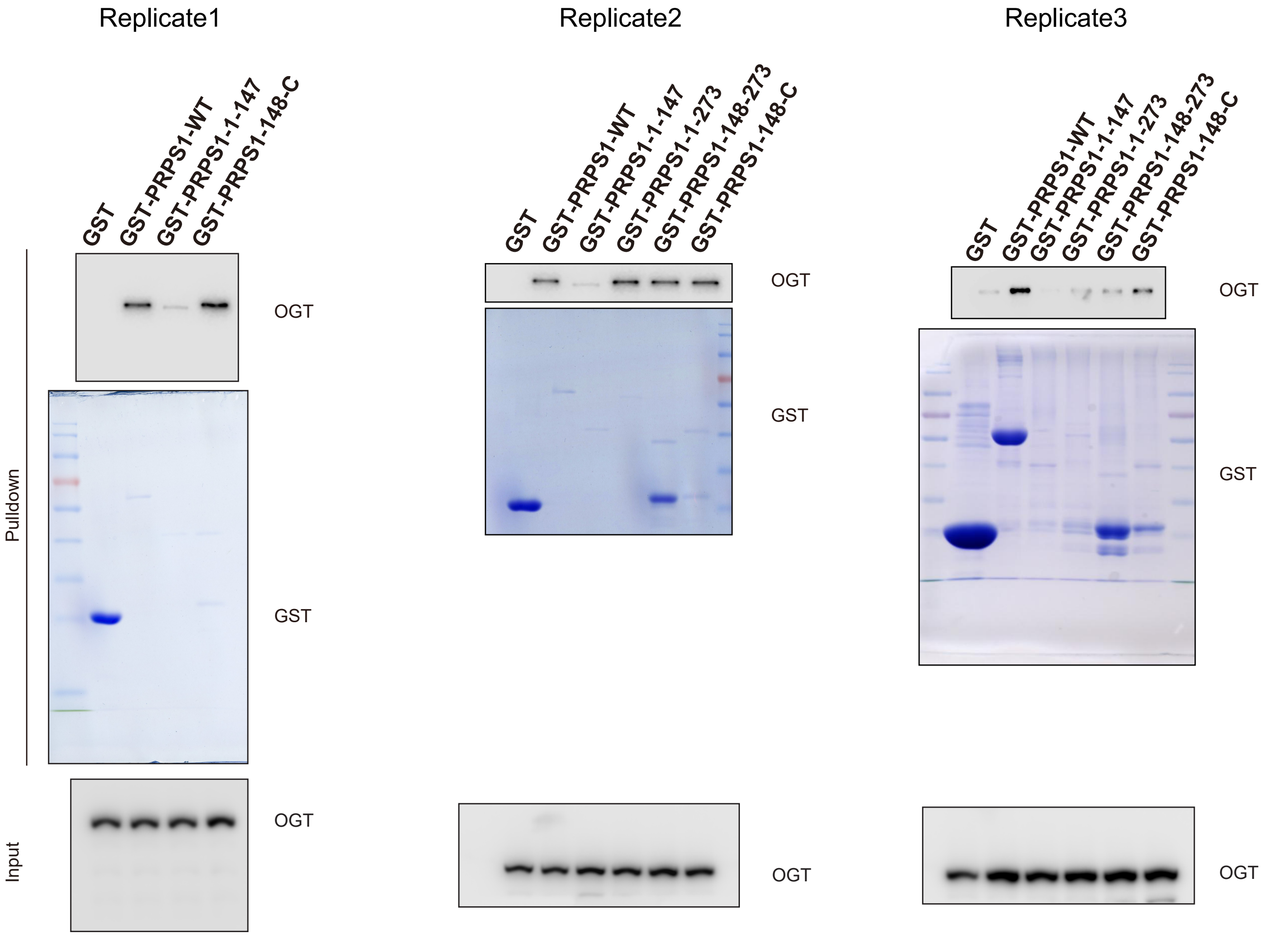

Extended Data Fig.2c

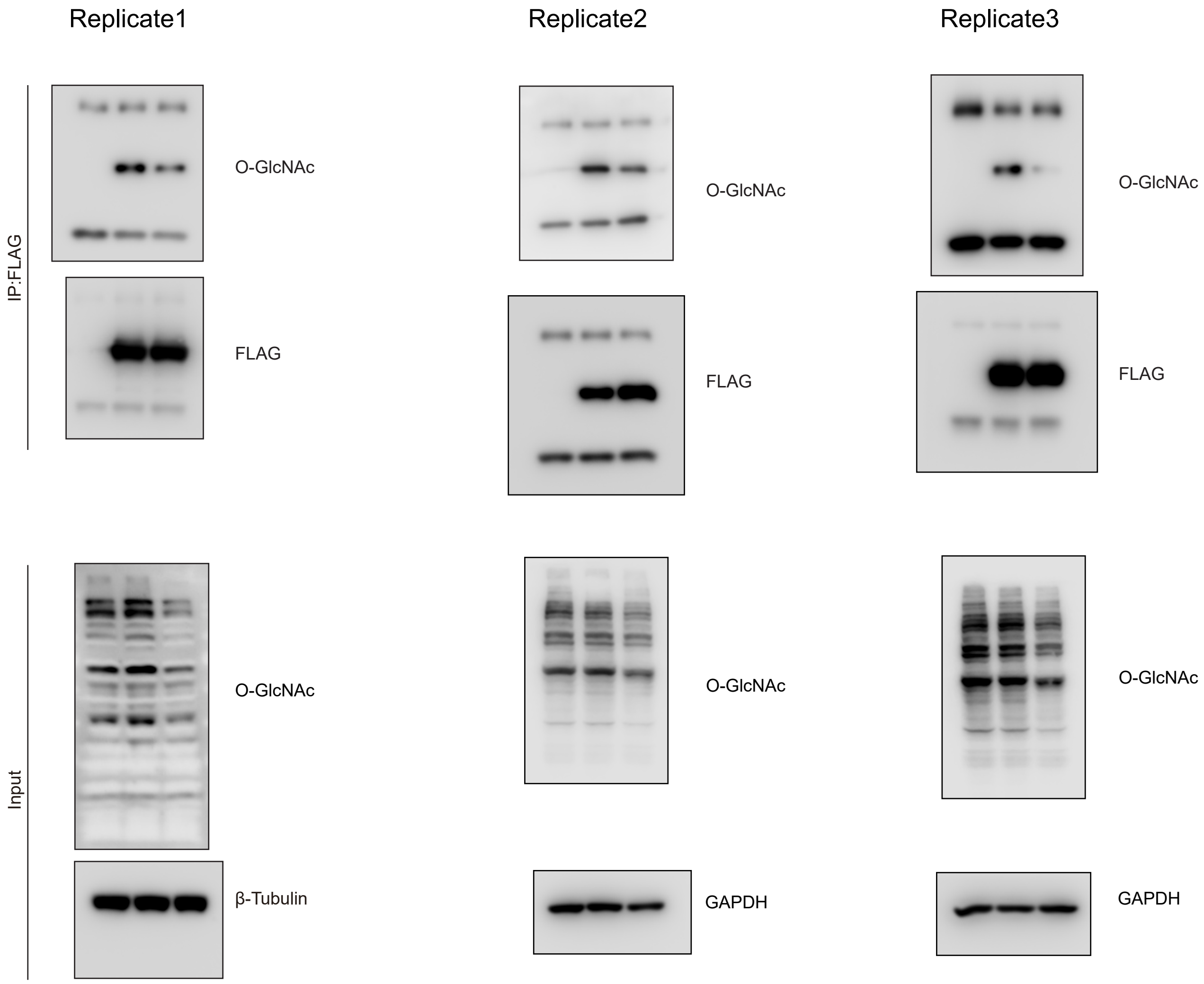

Extended Data Fig.2d

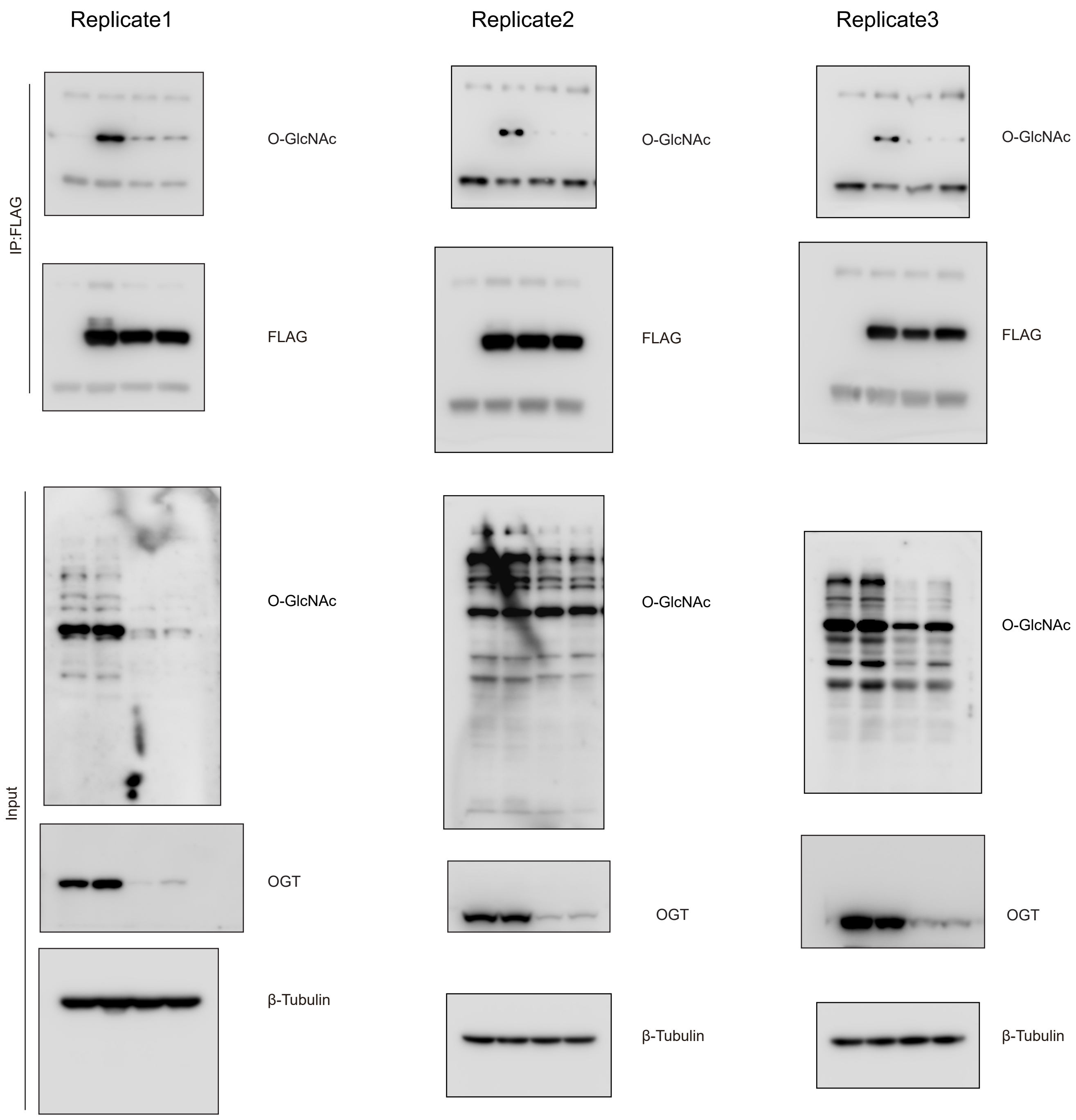

Extended Data Fig.2e

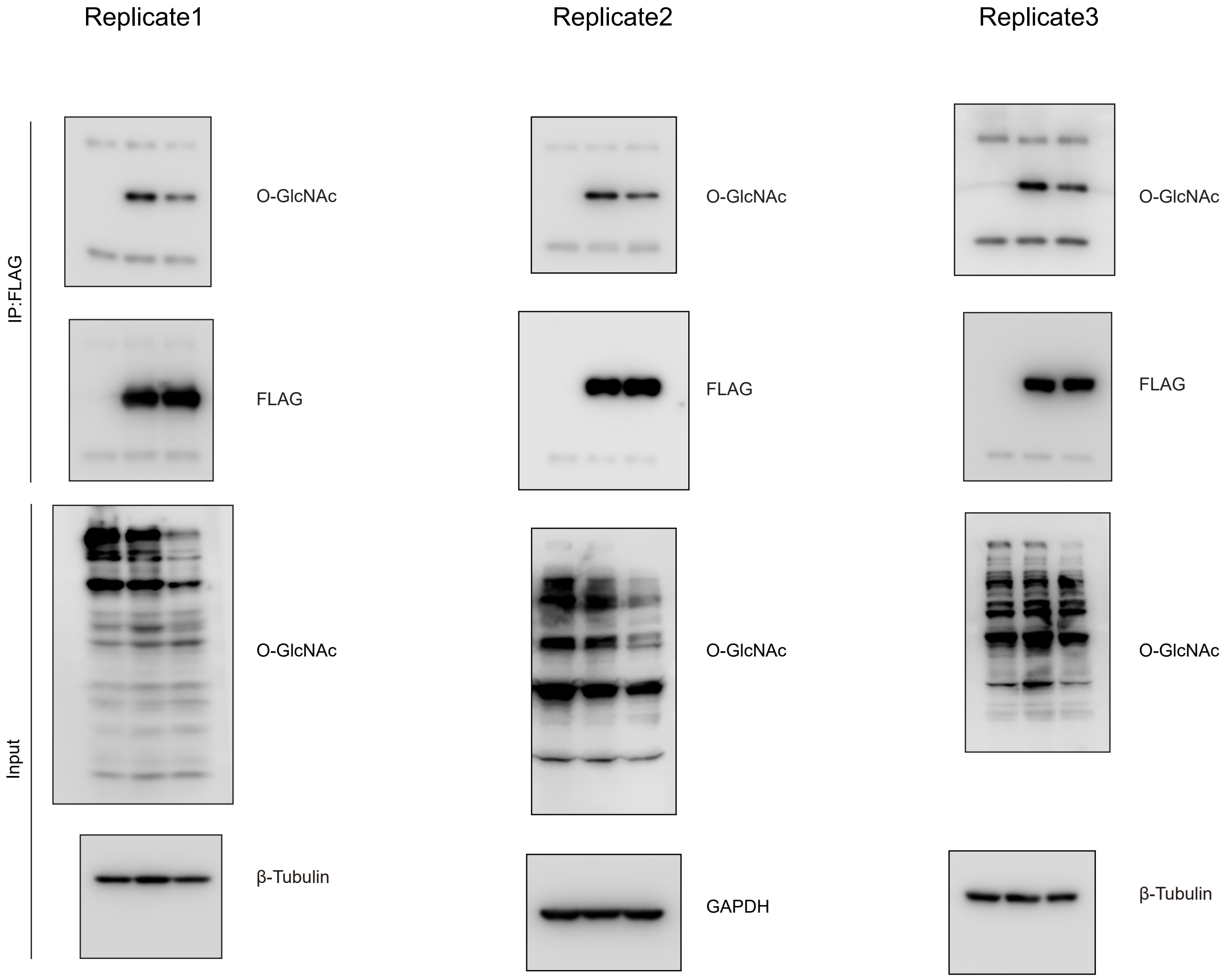

Extended Data Fig.2f

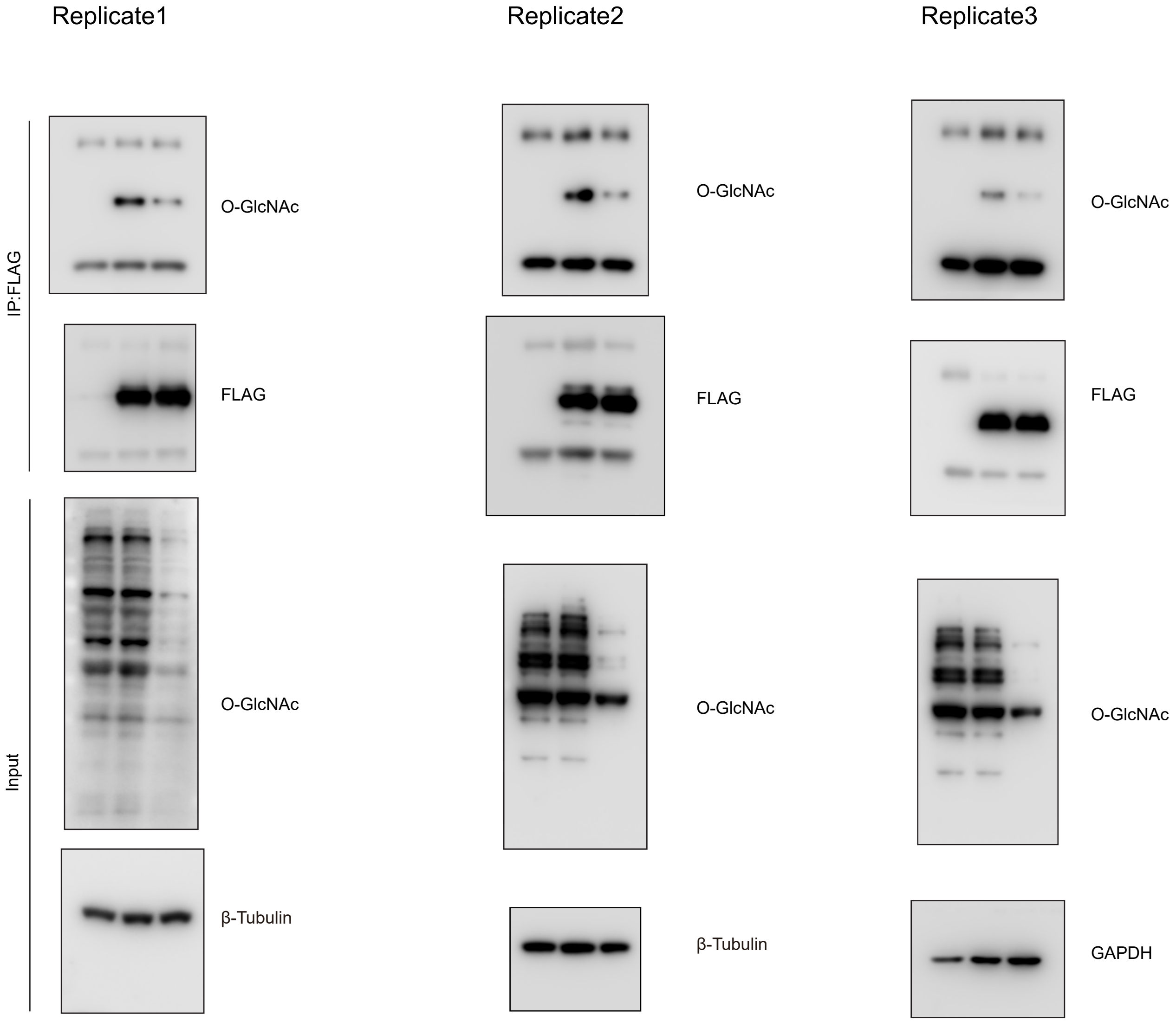

Extended Data Fig.2g

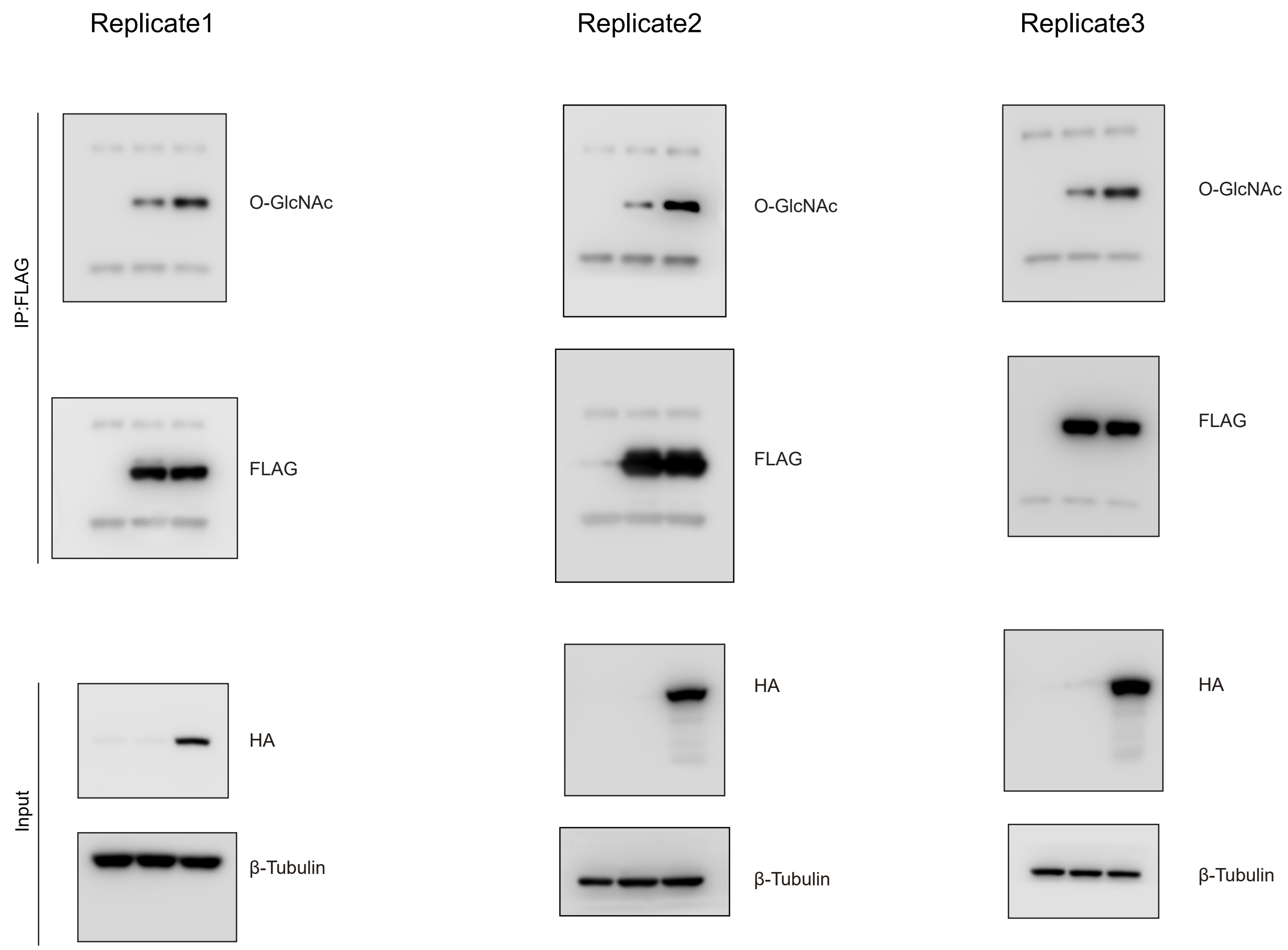

Extended Data Fig.2h

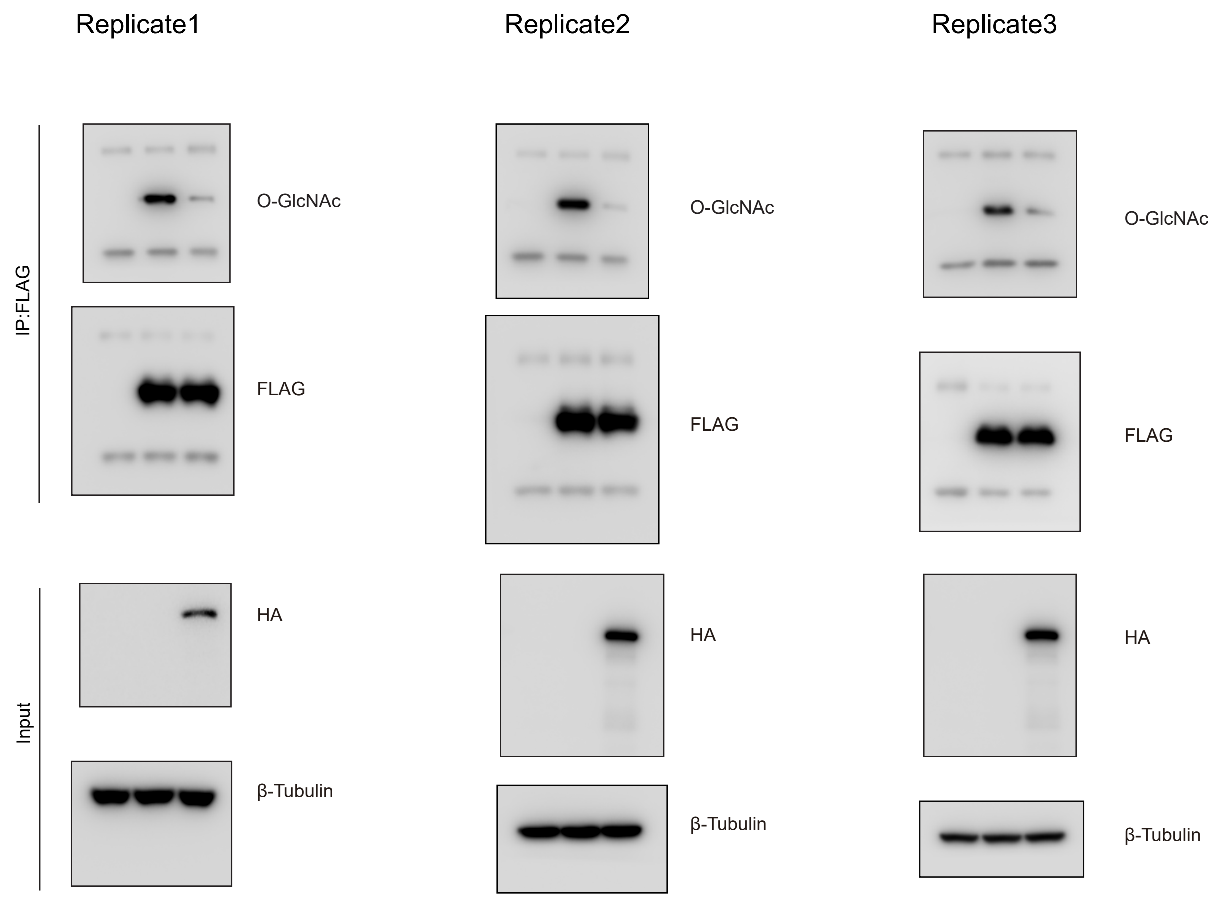

Extended Data Fig.2i

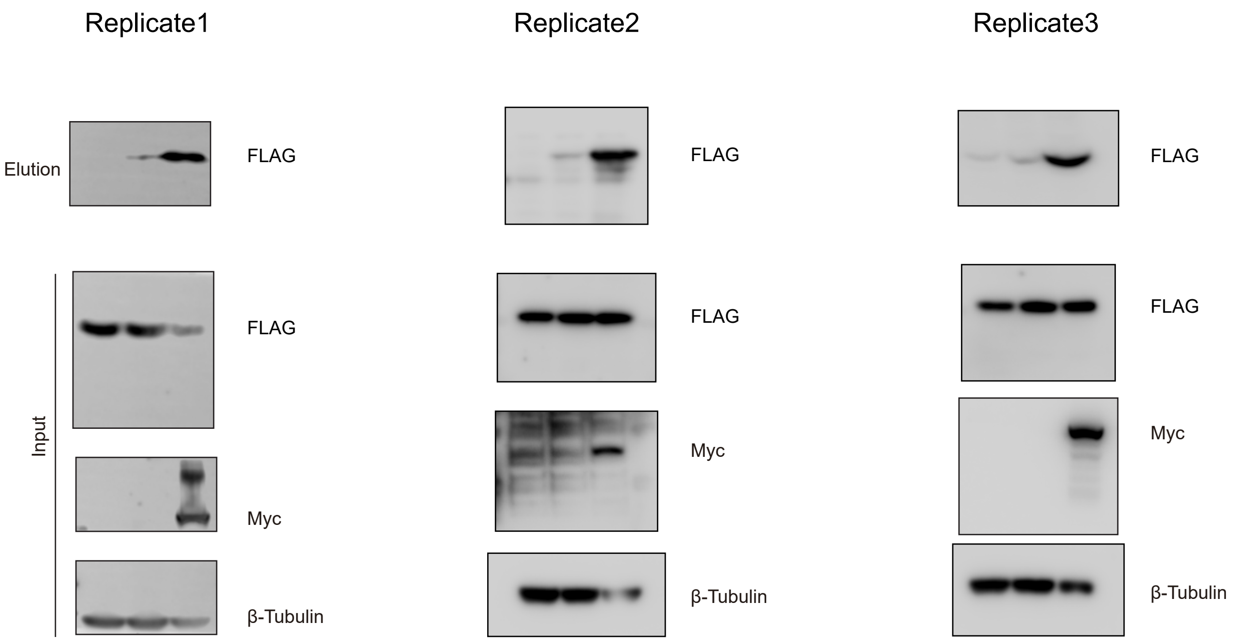

Supplement: Supplementary file 16 — Unprocessed western blots [file 41589_2023_1354_MOESM16_ESM.pdf]
