# Supplementary material for: Direct stimulation of de novo nucleotide synthesis by O-GlcNAcylation
Source: Nat Chem Biol. 2023 Jun 12;20(1):19–29. doi: 10.1038/s41589-023-01354-x (PMC10746546; doi:10.1038/s41589-023-01354-x)

Extended Data Fig. 4a

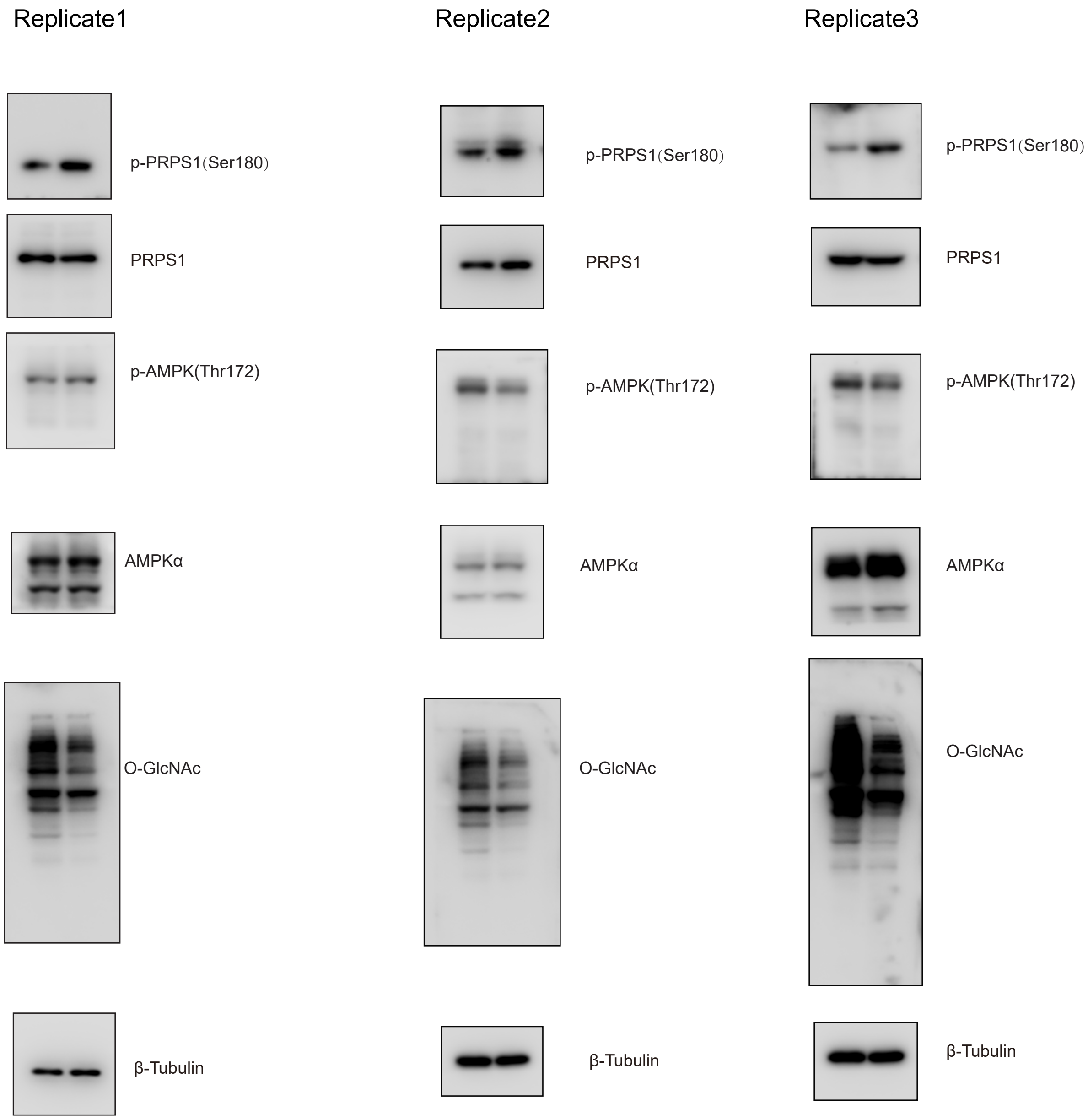

Extended Data Fig. 4d

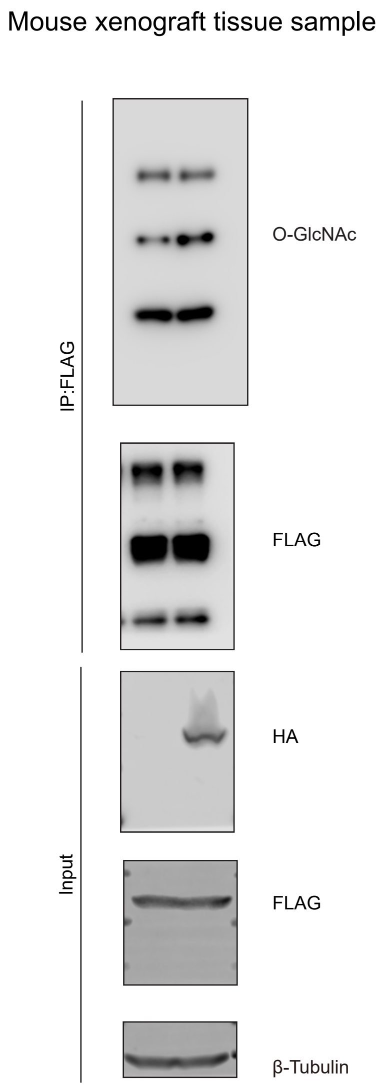

Extended Data Fig. 4h

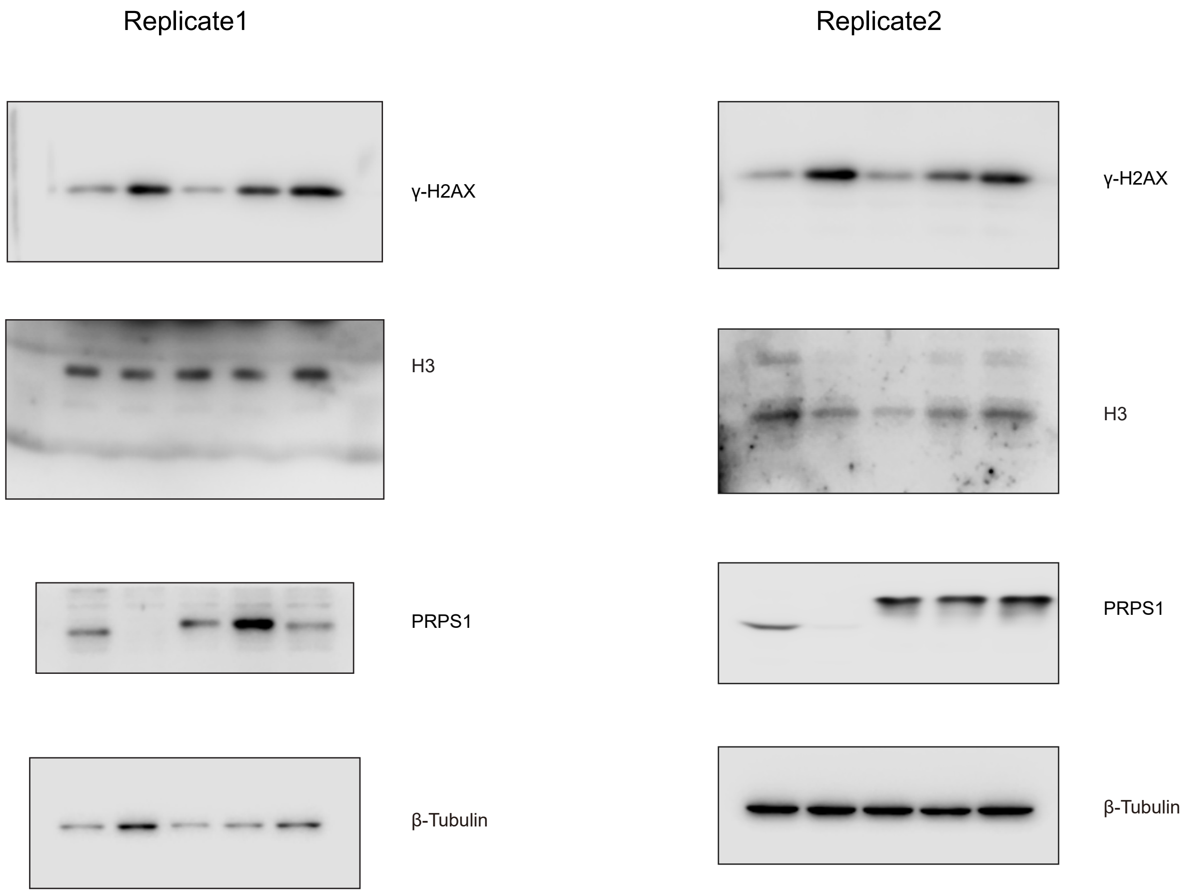

Extended Data Fig. 4i

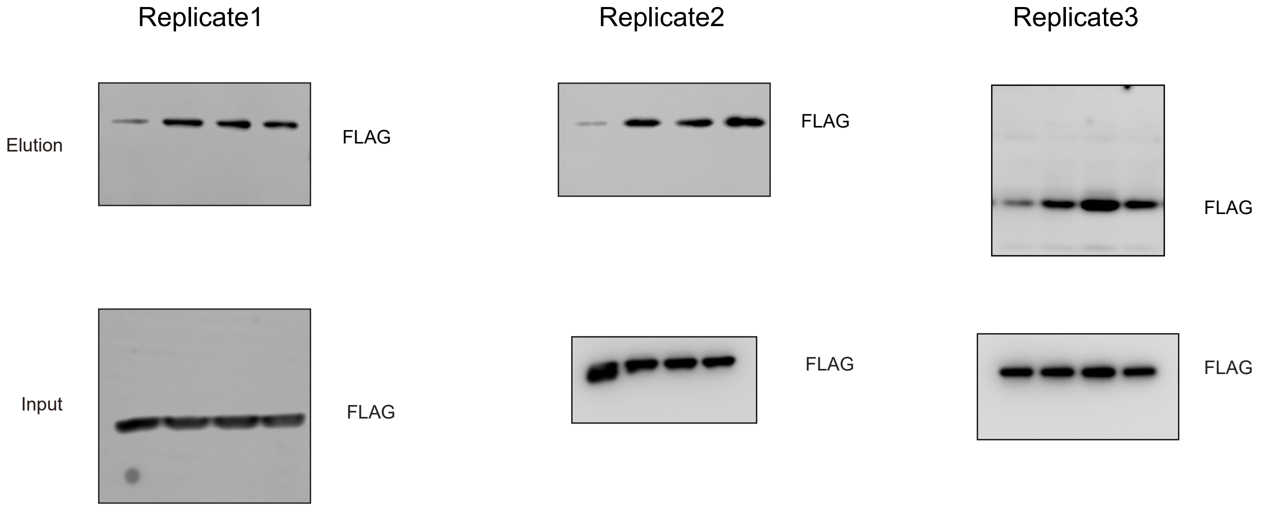

Extended Data Fig. 4j

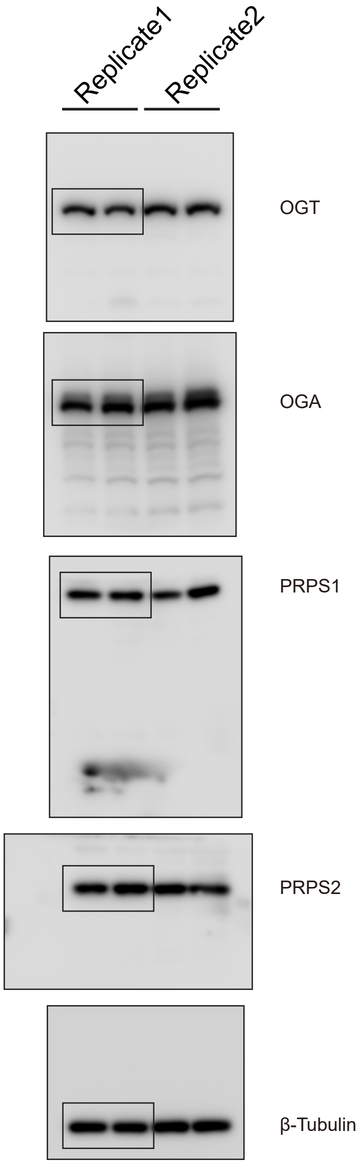

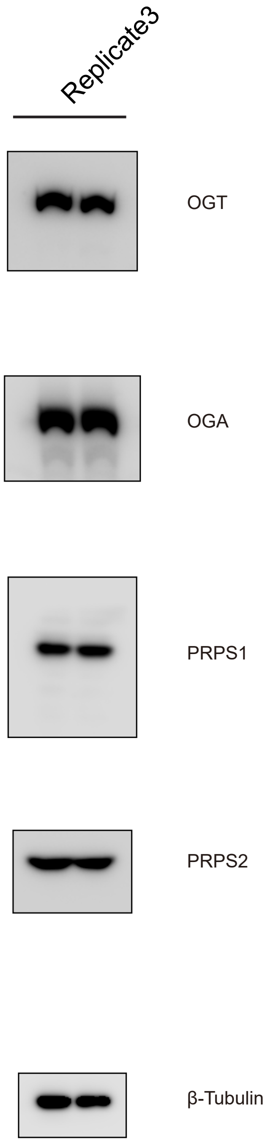

Extended Data Fig. 4k

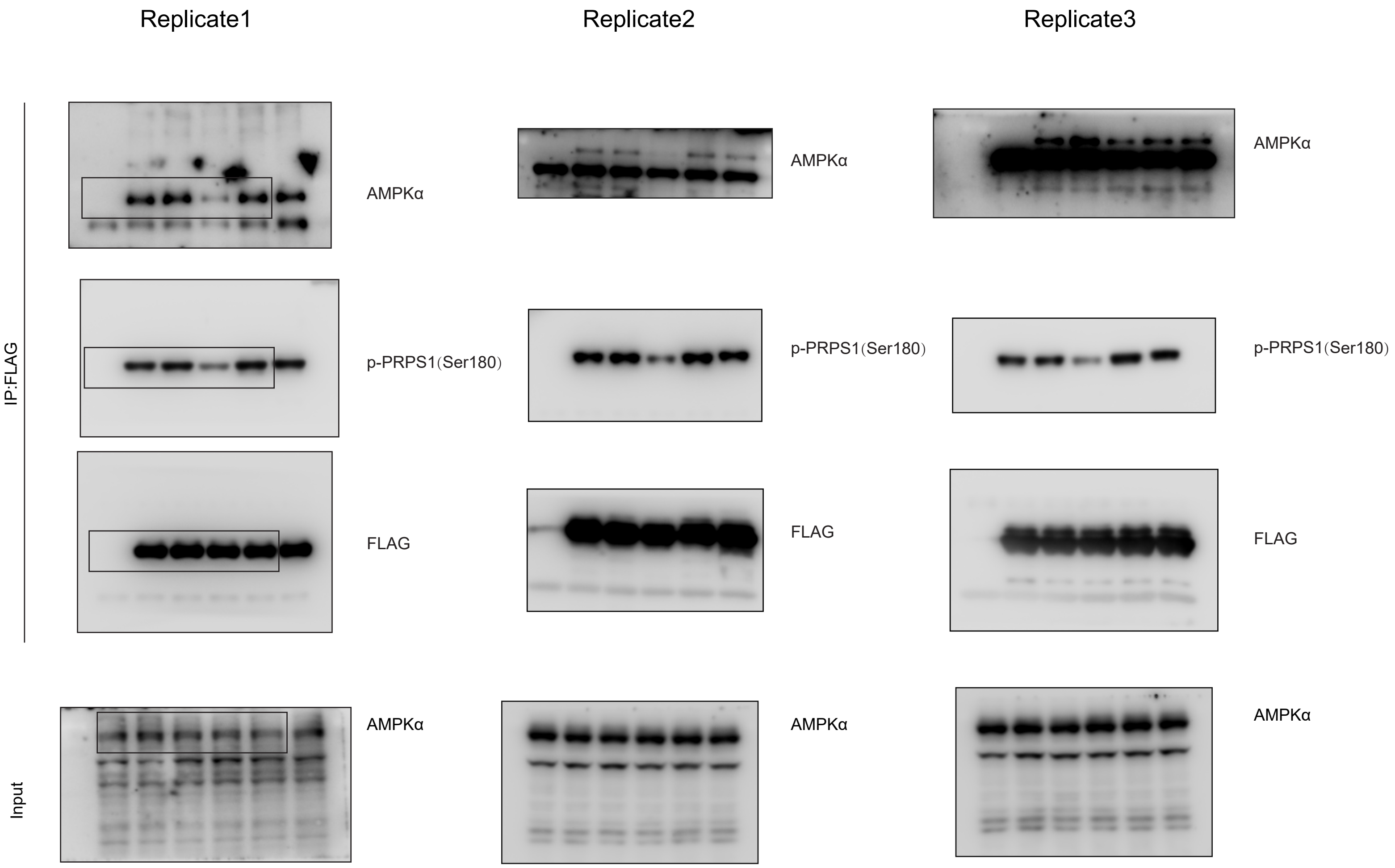

Supplement: Supplementary file 19 — Unprocessed western blots [file 41589_2023_1354_MOESM19_ESM.pdf]
